# Lineage-Specific Expansions of Retroviral Insertions within the Genomes of African Great Apes but Not Humans and Orangutans

**DOI:** 10.1371/journal.pbio.0030110

**Published:** 2005-03-01

**Authors:** Chris T Yohn, Zhaoshi Jiang, Sean D McGrath, Karen E Hayden, Philipp Khaitovich, Matthew E Johnson, Marla Y Eichler, John D McPherson, Shaying Zhao, Svante Pääbo, Evan E Eichler

**Affiliations:** **1**Department of Genetics, Case Western Reserve UniversityCleveland, OhioUnited States of America; **2**Department of Genome Sciences, University of Washington School of MedicineSeattle, WashingtonUnited States of America; **3**Max-Planck Institute for Evolutionary AnthropologyLeipzigGermany; **4**Department of Molecular and Human Genetics, Baylor College of MedicineHouston, TexasUnited States of America; **5**The Institute for Genome Research, BethesdaMarylandUnited States of America; Imperial CollegeUnited Kingdom

## Abstract

Retroviral infections of the germline have the potential to episodically alter gene function and genome structure during the course of evolution. Horizontal transmissions between species have been proposed, but little evidence exists for such events in the human/great ape lineage of evolution. Based on analysis of finished BAC chimpanzee genome sequence, we characterize a retroviral element (Pan troglodytes endogenous retrovirus 1 [PTERV1]) that has become integrated in the germline of African great ape and Old World monkey species but is absent from humans and Asian ape genomes. We unambiguously map 287 retroviral integration sites and determine that approximately 95.8% of the insertions occur at non-orthologous regions between closely related species. Phylogenetic analysis of the endogenous retrovirus reveals that the gorilla and chimpanzee elements share a monophyletic origin with a subset of the Old World monkey retroviral elements, but that the average sequence divergence exceeds neutral expectation for a strictly nuclear inherited DNA molecule. Within the chimpanzee, there is a significant integration bias against genes, with only 14 of these insertions mapping within intronic regions. Six out of ten of these genes, for which there are expression data, show significant differences in transcript expression between human and chimpanzee. Our data are consistent with a retroviral infection that bombarded the genomes of chimpanzees and gorillas independently and concurrently, 3–4 million years ago. We speculate on the potential impact of such recent events on the evolution of humans and great apes.

## Abstract 2

Comparison of human and other primate genomes provides evidence for a retroviral infection that bombarded the genomes of chimpanzees and gorillas 3-4 million years ago

## Introduction

Mammalian genomic sequence is littered with various classes of endogenous retroviruses that have populated genomes during the course of evolution [1,2]. In the case of humans, approximately 8.3% of the genome sequence consists of long terminal repeat (LTR) and endogenous retrovirus elements classified into more than 100 separate repeat families and subfamilies [3,4]. The bulk of human endogenous retrovirus elements are thought to have originated as a result of exogenous retrovirus integration events that occurred early during primate evolution. Based on comparative analyses of orthologous genomic sequence and sequence divergence of flanking LTR elements, the last major genomic infection of the human lineage is estimated to have occurred before the divergence of the Old World and New World monkey lineages (25–35 million years ago) [5,6,7,8]. Since the divergence of chimpanzee and human (5–7 million years ago), only one major family of human endogenous retroviruses (HERVK10) has remained active, and it has generated only three full-length copies with the open reading frame still intact [3]. While new insertions of endogenous retroviral sequences have been described [8,9], most of these are thought to have originated from other previously integrated retroelements [10] or longstanding associations with rare source virus [11]. This apparent wane in activity has led to the view that LTR retroposons have had a history of declining activity in the human lineage and are “teetering on the brink of extinction” [3].

Endogenous retroviruses may arise within genomes by at least two different mechanisms: retrotransposition from a pre-existing endogenous retrovirus (intraspecific transmission) or infection and integration via an exogenous source virus (horizontal transmission). Many cross-species transmissions have been documented and frequently manifest themselves as inconsistencies in the presumed phylogeny of closely related species. During the 1970s and 1980s, Benveniste and colleagues identified, by DNA hybridization and immunological cross-reactivity, several retroviral elements that could be found among more diverse primate/mammalian species but not necessarily among more closely related sister taxa [12,13,14]. Lieber and colleagues, for example, reported the isolation of a particular class of type C retroviruses from a woolly monkey (SSV-SSAV) and gibbon ape (GALV) but not the African great apes [13]. These viruses shared antigenic properties with previously described type C activated endogenous retroviruses of the Asian feral mouse *Mus caroli.* Cross-species infection from murines to primates was proposed as the likely origin of the retrovirus. A related endogenous retrovirus was subsequently identified in the koala, suggesting a zoonotic transmission from placentals to mammals [15]. Evidence of horizontal transmission for other families of retrovirus has been reported among classes of species as distantly related as avians and mammals [15].

Comparative analyses of closely related genomes have suggested that retroviral cross-species transmissions and genome integrations are a common occurrence during the recent evolutionary history of several species. Murine genomes, in particular, have been bombarded with relatively recent retroviral integrations [16]. In contrast to humans, there is ample evidence that exogenous retrovirus continues to bombard and fix within the genomes of Old World monkey species. Cross-species transmissions and genome integration of retroviruses as recent as 500,000 years ago have been reported between various simian species [17,18]. Differences in the distribution of endogenous retroviruses have even been noted between feral and domesticated mammalian species. The genomes of domestic cats, for example, harbor specific families of endogenous feline leukemia viruses that are not found in the genomes of wild cats [19]. Similarly, the PERV-C (porcine endogenous retrovirus type C) is restricted to domesticated pigs and has not been identified in the genomes of the wild boar from which domestication is thought to have occurred approximately 5,000 years ago [20].

From a functional perspective, the integration of retroviral sequence may have considerable impact. Endogenous retroviruses harbor cryptic mRNA splice sites, polyadenylation signals, and promoter and enhancer sequences. As such, their integration into the genome may significantly alter the expression patterns of nearby genes. Moreover, integrated retroviruses are often preferential sites of methylation and may promote rearrangement of DNA by way of non-allelic homologous recombination between elements. Consequently, these elements have been recognized as potent mutagens [2,21] that may significantly alter the phenotype [22,23]. The mechanism by which such elements originate and differentially spread among closely related species is, therefore, fundamental to our understanding of evolution.

## Results

### Distribution of Pan troglodytes Endogenous Retrovirus 1 among Primates

**Figure 1:**
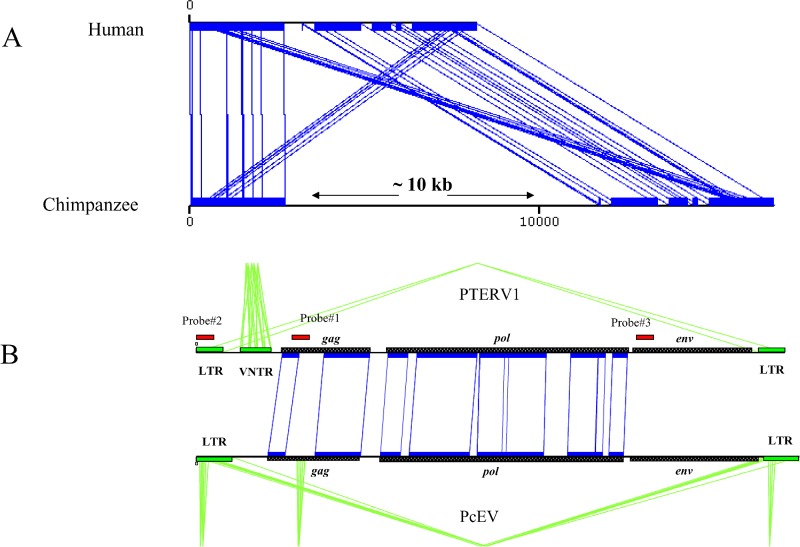
Identification and Sequence Analysis of PTERV1 (A) A graphical alignment of chimpanzee genomic sequence (AC097267) and an orthologous segment from human Chromosome 16 (Build 34) depicting an example of a PTERV1 (approximately 10 kb) insertion. Aligned sequences are shown in blue (miropeats) [47]. (B) The typical retroviral structure of the insert (*gag, pol, env,* and LTR) is compared to a baboon *(Papio cynocephalus)* endogenous retrovirus (PcEV). Regions of nucleotide homology are designated by black blocks and inter-sequence connecting lines. The location of probes (see Table S1) used in genomic library hybridizations, Southern blot analyses, and neighbor-joining tree analyses are shown (red).

During a comparison of human and chimpanzee BAC sequence, we identified several members of a full-length endogenous retrovirus family that were present in chimpanzee but absent in corresponding human genome sequence (Figure 1). Analysis of five full-length insertion sequences revealed that the endogenous retroviral elements (termed Pan troglodytes endogenous retrovirus 1 [PTERV1]) ranged in size from 5 to 8.8 kb in length (Materials and Methods). Translation of the sequence showed strong protein similarity to gammaretroviruses (53%–69%), in particular, murine leukemia virus, feline leukemia virus, porcine endogenous retrovirus type C, and baboon *(Papio cynocephalus)* endogenous retrovirus (Figure 1). Large deletions (1–2 kb) of the reverse transcriptase in some copies as well as the presence of multiple stop codons in all examined full-length copies indicate that this particular family of endogenous retrovirus is not replication competent.

**Figure 2:**
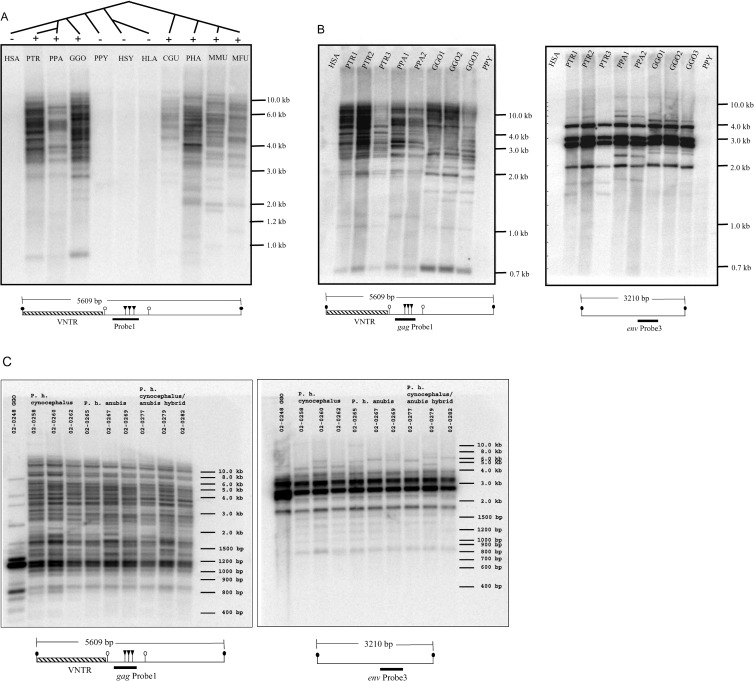
Southern Hybridization of PTERV1 among Primates Species represented include human (HSA), common chimpanzee (PTR), bonobo (PPA), gorilla (GGO), orangutan (PPY), siamang (HSY), white-handed gibbon (HLA), Abyssinian black-and-white colobus monkey (CGU), olive baboon (PHA), rhesus macaque (MMU), and Japanese macaque (MFU). Below each panel, a restriction map (chimpanzee sequence AC097267) is presented in relation to the hybridization probe: PstI (closed circles), PvuII (open circles), and HpaII/MspI (triangles) (see Figures S1 and S2 for additional details). (A) The absence of PTERV1 among Asian apes and humans is shown in contrast to a generally accepted catarrhine species phylogeny. Primate DNAs have been digested with PstI restriction enzyme, Southern-transferred to nylon membrane, and hybridized with PTERV1 *gag* probe number 1. (B) Multiple African great ape species are compared for both the *gag* probe number 1 and *env* probe number 3 (Figure 1). Proximity of probe number 1 to the VNTR, which is variable in length between copies (400 bp to 10 kb), reveals hundreds of insertion sites. (C) Multiple individuals from different subspecies of the olive baboon are compared for both *gag* probe number 1 and *env* probe number 3. The pattern of Southern hybridization shows limited intra-specific variation, indicative of either polymorphism in restriction enzyme sites or copy number variation.

We designed two probes to the *gag* and *env* portions of PTERV1 (Figure 1; Table S1) and assessed the distribution of PTERV1 among apes and Old World monkeys by Southern analysis. More than 100 copies of the endogenous retrovirus were detected in each African ape and Old World monkey species (Figures 2 and Figure S1). Comparison between DNA digested with methylation-sensitive and -resistant restriction enzymes indicated that most copies were extensively methylated in these species (Figure S2). In contrast, analysis of multiple Asian apes (siamang, gibbon, and orangutan) and a panel of human DNAs showed no hybridization signal. These findings were consistent with early DNA hybrid melting experiments [12] and DNA hybrid electron microscopic studies [14] that indicated that DNA from the African great apes harbored sequences homologous to both colobus monkey and baboon exogenous retroviruses while the genomes of man and Asian apes did not. These data were sometimes used as supporting evidence for an Asian origin of modern humans [12].

**Figure 3:**
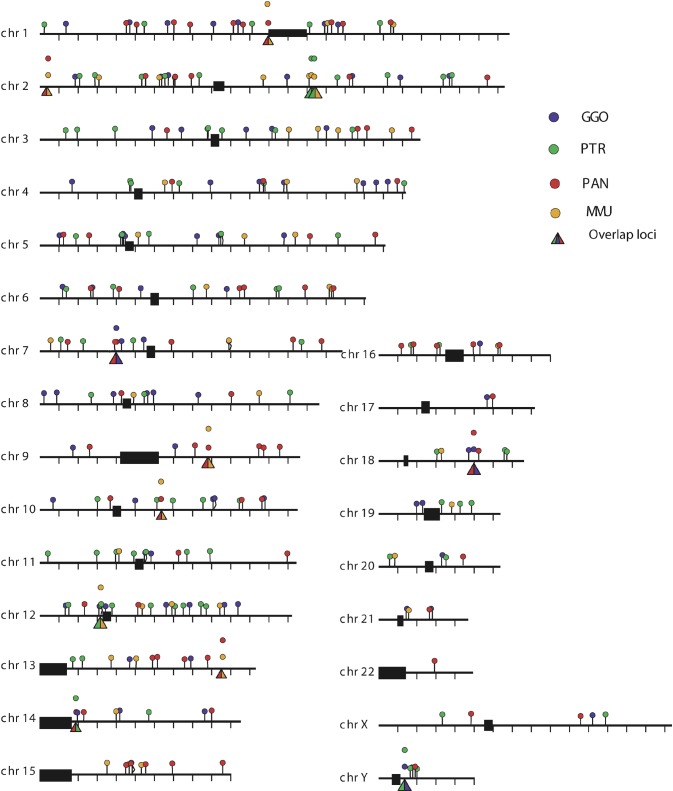
PTERV1 Insertion Sites Large-insert genomic clones that contained full-length endogenous retrovirus were identified by hybridization from four species: chimpanzee (PTR), gorilla (GGO), baboon (PAN), and rhesus macaque (MMU). End sequencing of large-insert clones (*n* = 1,467) and alignment against the human genome reference sequence identified 287 insertion sites (see Table S2). A total of 95.8% of these sites were non-orthologous when compared between species. chr, chromosome.

In order to further resolve the evolutionary relationship of these endogenous retroviruses, we compared the sites of retroviral integration in the genomes of chimpanzee, gorilla, macaque, and baboon. To this end, we screened large-insert genomic (BAC) libraries for each species using multiple probes from the PTERV1 reference sequence (Table S1). These data allowed us to estimate copy number in each species and to distinguish clones harboring full-length retroviral inserts versus solo LTR elements (Table 1). In addition, we used BAC end sequences from nonhuman primate clones harboring full-length retroviruses to map their locations back to the human genome. We then compared the locations (Figure 3; Table 2) between species to determine whether the sites were non-orthologous. Based on an analysis of 1,467 large-insert clones, we mapped 299 retroviral insertion sites among the four species (Figure 3; Table S2). A total of 275 of the insertion sites mapped unambiguously to non-orthologous locations (Table 2), indicating that the vast majority of elements were lineage-specific (i.e., they emerged after the divergence of gorilla/chimpanzee and macaque/baboon from their common ancestor).

**Table 1:**
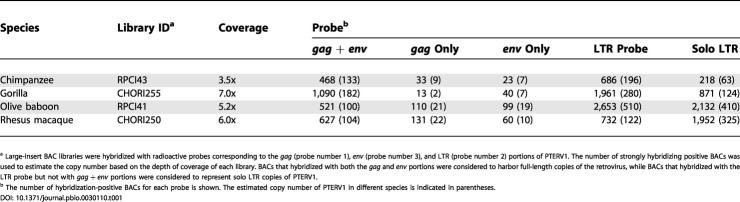
PTERV1 BAC Library Hybridizations: Number of BACs (Estimated Copy Number)

^a^ Large-insert BAC libraries were hybridized with radioactive probes corresponding to the *gag* (probe number 1), *env* (probe number 3), and LTR (probe number 2) portions of PTERV1. The number of strongly hybridizing positive BACs was used to estimate the copy number based on the depth of coverage of each library. BACs that hybridized with both the *gag* and *env* portions were considered to harbor full-length copies of the retrovirus, while BACs that hybridized with the LTR probe but not with *gag* + *env* portions were considered to represent solo LTR copies of PTERV1

^b^ The number of hybridization-positive BACs for each probe is shown. The estimated copy number of PTERV1 in different species is indicated in parentheses

**Table 2:**
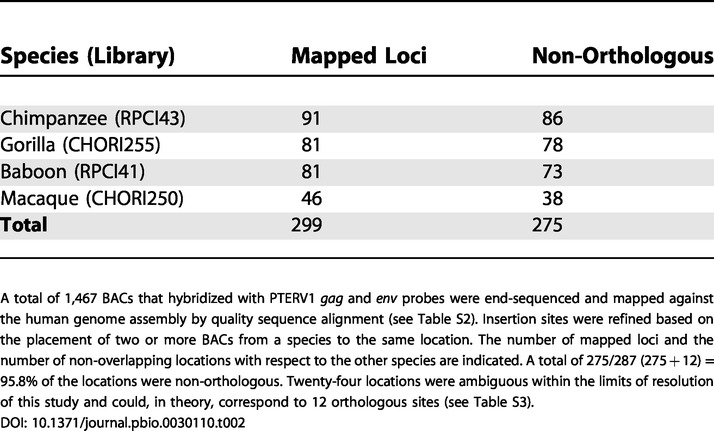
Cross-Species Retroviral Insertion Mapping

A total of 1,467 BACs that hybridized with PTERV1 *gag* and *env* probes were end-sequenced and mapped against the human genome assembly by quality sequence alignment (see Table S2). Insertion sites were refined based on the placement of two or more BACs from a species to the same location. The number of mapped loci and the number of non-overlapping locations with respect to the other species are indicated. A total of 275/287 (275 + 12) = 95.8% of the locations were non-orthologous. Twenty-four locations were ambiguous within the limits of resolution of this study and could, in theory, correspond to 12 orthologous sites (see Table S3)

Within the limits of this BAC-based end-sequencing mapping approach, 24 sites mapped to similar regions of the human reference genome (approximately 160 kb) and could not be definitively resolved as orthologous or non-orthologous (Table S3). We classified these as “ambiguous” overlap loci (Figure 3). If all 24 locations corresponded to insertions that were orthologous for each pair, this would correspond to a maximum of 12 orthologous loci. The number of non-orthologous loci was calculated as 275/287 (275 + 12) or 95.8%. This is almost certainly a lower-bound estimate owing to the limitation of our BAC-based mapping approach to refine the precise locations of the insertions.

We performed two analyses to determine whether these 12 shared map intervals might indeed be orthologous. First, we examined the distribution of shared sites between species (Table S3). We found that the distribution is inconsistent with the generally accepted phylogeny of catarrhine primates [5]. This is particularly relevant for the human/great ape lineage. For example, only one interval is shared by gorilla and chimpanzee; however, two intervals are shared by gorilla and baboon; while three intervals are apparently shared by macaque and chimpanzee. Our Southern analysis shows that human and orangutan completely lack PTERV1 sequence (see Figure 2A). If these sites were truly orthologous and, thus, ancestral in the human/ape ancestor, it would require that at least six of these sites were deleted in the human lineage. Moreover, the same exact six sites would also have had to have been deleted in the orangutan lineage if the generally accepted phylogeny is correct. Such a series of independent deletion events at the same precise locations in the genome is unlikely (Figure S3).

For the three intervals putatively shared between macaque and chimpanzee, we attempted to refine the precise position of the insertions by taking advantage of the available whole-genome shotgun sequences for these two genomes. For each of the three loci, we mapped the precise insertion site in the chimpanzee and then examined the corresponding site in macaque (http://www.ncbi.nlm.nih.gov). In one case, we were unable to refine the map interval owing to the presence of repetitive rich sequences within the interval. In two cases, we were able to refine the map location to single basepair resolution (Figures S4 and S5). Based on this analysis, we determined that the sites were not orthologous between chimpanzee and macaque. It is interesting to note that this level of refined mapping in chimpanzee revealed 4- to 5-bp AT-rich target site duplications in both cases. These findings are consistent with an exogenous retrovirus source since proviral integrations typically target AT-rich DNA ranging from 4 to 6 bp in length [24]. Although the status of the remaining overlapping sites is unknown, these data resolve four additional sites as independent insertion events and suggest that the remainder may similarly be non-orthologous. This apparent independent clustering of retroviral insertions at similar locations may be a consequence of preferential integration bias or the effect of selection pressure against gene regions, limiting the number of effective sites that are tolerated for fixation.

### Phylogenetic Analysis of PTERV1

**Figure 4:**
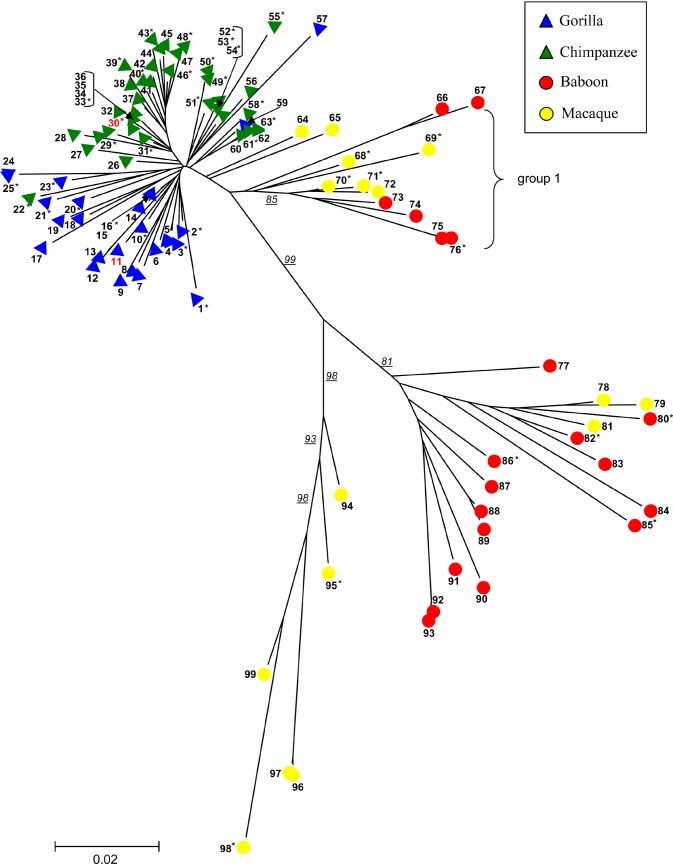
PTERV1 Phylogenetic Tree Portions of the *gag* and *env* genes (about 823 bp) were resequenced from 101 PTERV1 elements from common chimpanzee (*n* = 42), gorilla (*n* = 25), rhesus macaque (*n* = 14), and olive baboon (*n* = 20). A neighbor-joining phylogenetic tree shows a monophyletic origin for the gorilla and chimpanzee endogenous retroviruses but a polyphyletic origin among the Old World monkey species. Bootstrap support (*n* = 10,000 replicates) for individual branches are underlined. Although the retroviral insertions have occurred after speciation, retroviral sequences show greater divergence than expected for a non-coding nuclear DNA element (see Table S4). Table S8 provides a clone key for number designation. Phylogenetic trees showing the *gag, env,* and LTR segments separately are presented in Figure S6. Sequences 11 and 30 (red) are mapped to one of the 12 ambiguous overlapping loci described in the text (see Table S3). They do not cluster in this phylogenetic tree, which indicates that they are unlikely to be true orthologs.

We next examined the phylogenetic relationship of the retroviral elements by comparing portions of the *gag* and *env* regions. We chose a total of 103 BAC clones representing distinct loci from the four species and PCR-amplified and sequenced noncontiguous *gag* and *env* portions (823 bp) from each clone. Based on sequence analysis, each of the 101 BAC clones contained a single copy of the *gag* and *env* portions as determined by analysis of the sequence. These were deemed to be linked to the same endogenous retrovirus insert. Two clones showed “heterozygous” sequence signatures consistent with two or more copies clustered within the BAC clone and were excluded from further analysis. We constructed a phylogenetic tree based on the multiple sequence alignment (Figure 4) of the concatenated *gag* and *env* regions. (A similar tree topology with less bootstrap support is obtained if *env* and *gag* segments are considered separately [Figure S6A and S6B].) While it is clear that this particular class of endogenous retroviruses shares a common origin, the retroviral phylogeny is inconsistent with the generally accepted primate species tree based on molecular data [5]. The chimpanzee and gorilla PTERV1 elements are most closely related (3%–4% divergence) and belong to a single phylogenetic clade (Table S4). In contrast, macaque and baboon inserts show considerably greater sequence divergence (9%–11%) and a much more stratified phylogeny with little discrimination based on species. This tree topology suggests a polyphyletic origin with at least three groups of Old World virus being distinguished (Figure 4). Interestingly, one of these Old World groups (group 1) shows a possible monophyletic origin with respect to chimpanzee and gorilla.

Since *gag* and *env* regions of viruses may experience vastly different selection pressures, we repeated the analysis by examining 295 bp of LTR sequence from a subset of 55 loci (Table S4). Phylogenetic analysis of the LTR segment between species revealed a virtually identical tree topology (Figure S6C) to that of the concatenated *gag* and *env* segments. Macaque and baboon interproviral divergence (14%–24%), once again, was significantly greater than that for chimpanzee and gorilla (3%–4%). It should be noted that this degree of divergence is 2-fold greater than the average divergence for a neutral site between gorilla and chimpanzee (approximately 1.6%) [25] and approximately 10-fold greater than estimated neutral divergence rates between macaque and baboon (1.5%, E. E. E., unpublished data).

**Figure 5:**
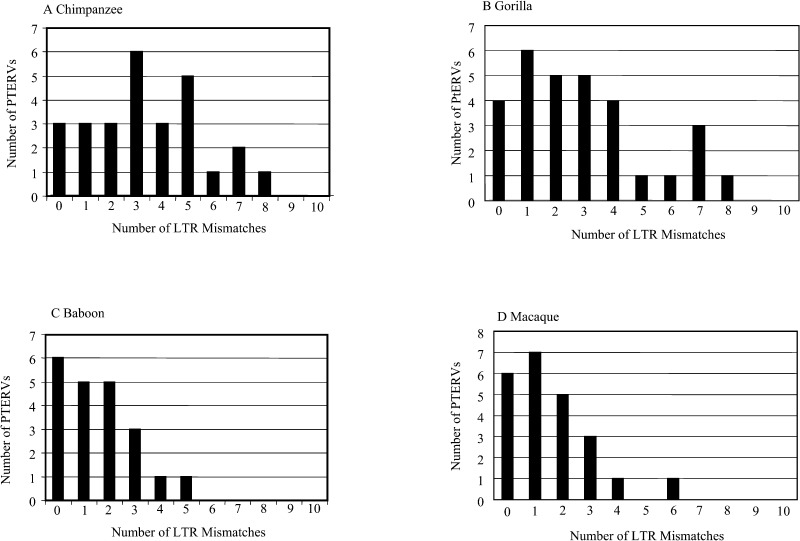
LTR Variation A total of 101 loci that contained full-length PTERV1 elements were examined for the number of mismatches between left and right LTR flanks (295 bp). Different distributions were obtained for Old World monkeys (baboon, mean = 1.6 ± 1.4; macaque, mean = 1.6 ± 1.5) and great ape species (chimpanzee, mean = 3.4 ± 2.2; gorilla, mean = 2.9 ± 2.3).

Identical copies of LTR sequences are created as part of the retrovirus life cycle [2,8]. Consequently, divergence of flanking LTR elements has been used extensively as a metric to estimate the evolutionary age of the source infection. We compared intraproviral LTR sequence divergence from 101 loci in the chimpanzee, gorilla, baboon, and macaque genomes (Figure 5). We designed oligonucleotides within conserved portions of the LTR sequence alignment, PCR-amplified the LTR flanks, sequenced both products from each clone, and compared them for mismatches. Gorilla and chimpanzee LTR elements showed a median divergence of 0.98% and 1.15%, respectively. Using neutral estimates of primate LTR divergence [8], we estimate that a contemporaneous infection occurred in these ancestral gorilla and chimpanzee lineages 3–4 million years ago (see Materials and Methods). LTR divergence among baboon and macaque was significantly less (0.051% and 0.058%, respectively; *p* < 0.007, one-tailed *t* test), corresponding to a much more recent origin (approximately 1.5 million years ago). These observations may be reconciled with differences in the phylogenetic tree topology if the Old World monkeys were infected by several diverged viruses while gorilla and chimpanzee were infected by a single closely related exogenous source. In this scenario, most of the genetic differences observed among macaque and baboon endogenous retroviruses are not nuclear in origin but arose as part of normal variation between viruses (see Materials and Methods).

We examined the distribution pattern of intraproviral LTR divergence to determine whether the observed pattern was consistent with a single or multiple infections. We modeled the probability of a mutation occurring within a LTR sequence using the Poisson distribution (Table S5). For each of the four species, the distribution of LTR mismatches did not differ significantly from normal statistical fluctuations (Poisson distribution, *p* > 0.1). While these results are consistent with a single burst of retroviral insertions within each lineage, we cannot exclude the possibility of multiple integrations over a shorter span of 1–2 million years generating a virtually identical pattern. Indeed, based on the presence and absence of hybridizing bands among individuals from the same species (see Figure 2B and 2C), we estimate that 5%–10% of the sites are polymorphic within the baboon and chimpanzee populations. This may be the result of deletion or more recent reinfections of the germline.

### PTERV1 Integration Bias and Gene Expression

Integration of retroviral sequence into genomes has long been recognized as a potent mutagen due to the fact that such proviruses frequently alter transcription, disrupt splicing, or become targets of hypermethylation. In this study, for example, the majority of full-length retroviral elements were heavily methylated (see Figure S2), suggesting that most copies had been transcriptionally silenced in transformed and peripheral blood lymphocytes. Studies of proviral integrations followed by inbreeding suggest that 5%–10% of novel insertions result in phenotypic change and/or alter gene expression [22,23]. We examined the distribution of full-length retroviruses within the chimpanzee genome and identified 107 sites of integration (Tables S6 and S7). We defined an integration site as genic if it mapped between the transcription start and stop site of a known annotated human gene. Only 13% (14/107) of retroviral integrations mapped within the introns of genes. Another 75 of the 107 insertion sites were located more than 50 kb upstream or downstream from a transcription start site. This is a significant departure (*p* < 0.001) from a random model of intron integration (29.2%) and shows a distinctly opposite trend to patterns of somatic retroviral insertion, for which gene-rich regions of the genome are strongly favored (34%–60% of murine leukemia virus and HIV infections) [26,27] (Figure S7). We propose that this 3- to 6-fold bias against gene-rich regions is the direct result of strong purifying selection pressure on the ancestral chimpanzee population.

In order to determine whether the small subset of genes (*n* = 14) that had been targets of insertions also showed significant changes in expression, we examined gene expression data for humans and chimpanzees in five tissues. This dataset was controlled for DNA sequence single-basepair differences between human and chimpanzee by excluding all probes that did not match perfectly between the two species (P. K and I. Hellman, unpublished data; [28]). Six of the ten genes detected in this dataset showed significant differences in expression levels between human and chimpanzee tissues (Table 3). In five of the six cases, the genes showed reduced levels of gene expression in the chimpanzee when compared to human. A simulation study based on the total number of differentially expressed (approximately 30%) genes revealed that this enrichment is weakly significant (*p* = 0.0489). The results suggest that retroviral insertion may have had an influence on expression difference between humans and chimps, but because of the small sample size (ten genes), it should be cautioned that the results are far from definitive. Additional studies, including RT-PCR and Northern analysis, with carefully controlled probes and matched tissue sources will be required to fully address this issue.

**Table 3:**
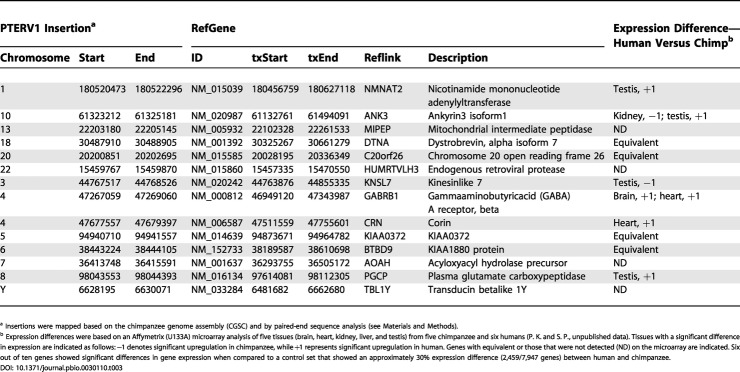
Retroviral Insertions That Map within Genes in the Chimpanzee Genome Assembly

^a^ Insertions were mapped based on the chimpanzee genome assembly (CGSC) and by paired-end sequence analysis (see Materials and Methods)

^b^ Expression differences were based on an Affymetrix (U133A) microarray analysis of five tissues (brain, heart, kidney, liver, and testis) from five chimpanzee and six humans (P. K. and S. P., unpublished data). Tissues with a significant difference in expression are indicated as follows: −1 denotes significant upregulation in chimpanzee, while +1 represents significant upregulation in human. Genes with equivalent or those that were not detected (ND) on the microarray are indicated. Six out of ten genes showed significant differences in gene expression when compared to a control set that showed an approximately 30% expression difference (2,459/7,947 genes) between human and chimpanzee

## Discussion

Most human endogenous retroviruses are thought to have emerged as a result of ancient infections more than 25 million years ago [7,8], followed by subsequent retrotransposition events. Several lines of evidence indicate that chimpanzee and gorilla PTERV1 copies arose from an exogenous source. First, there is virtually no overlap (less than 4%) between the location of insertions among chimpanzee, gorilla, macaque, and baboon, making it unlikely that endogenous copies existed in a common ancestor and then became subsequently deleted in the human lineage and orangutan lineage. Second, the PTERV1 phylogenetic tree is inconsistent with the generally accepted species tree for primates, suggesting a horizontal transmission as opposed to a vertical transmission from a common ape ancestor. An alternative explanation may be that the primate phylogeny is grossly incorrect, as has been proposed by a minority of anthropologists [29]. This seems unlikely in light of the extensive molecular evolutionary data that have been collected over the last few years [5,25] that clearly place orangutan as the outgroup species to the human–chimpanzee–gorilla clade and Old World monkeys as an outgroup to the human/ape lineage.

Third, the single nucleotide substitution rate for the viruses is significantly greater than what would be expected for neutral nuclear DNA. The extent of chimpanzee and gorilla substitution, for example, has been estimated at approximately 0.016 ± 0.008 substitutions per site [25], with approximately half of this variation occurring in each lineage. The endogenous retrovirus sequence that we examined showed significantly greater divergence (0.038 ± 0.003) (62 pairwise comparisons). A similar excess of divergence was observed if only intraspecific retroviral divergence was compared (0.028 ± 0.003 and 0.041 ± 0.003 for chimpanzee and gorilla, respectively) (see Table S4). Such an acceleration of neutral substitution would easily be explained if it were composed of a viral and nuclear component (see Materials and Methods). Fourth, if we partition synonymous and non-synonymous substitution sites (see Materials and Methods), we observe a deficiency of amino acid replacement sites (*K*_a_/*K*_s_ = 0.63). We observed a similar result (*K*_a_/*K*_s_ = 0.44) for one of the ambiguous overlap loci shared between gorilla and chimpanzee (see Table S3). This significant departure from neutrality would be an expected residuum if a portion of PTERV1 sequence variation accrued while being propagated as an infectious virus [11]. If it were solely derived from an ancestral endogenous element, a neutral pattern, as opposed to a relaxed pattern of purifying selection, would be expected. Finally, in the few examples where the insertion sites have been mapped precisely, both the length and the composition of the target site duplications are characteristic of the patterns of retroviral integrations [24].

While these multiple lines of indirect data indicate that PTERV1 likely emerged from an exogenous source, its source reservoir, if it still exists, is unknown. PTERV1 does not share high sequence identity to any known retrovirus. Translation of the protein-encoding portions shows sequence similarity (approximately 50%) to feline leukemia viruses, murine leukemia viruses, and the baboon endogenous retrovirus. Such sequences are known to transfer frequently between the soma of species and occasionally enter the germline [17,18,19,20]. It is interesting that one of the three main branches of Old World monkey PTERV1 may actually share a monophyletic origin with the gorilla and chimpanzee elements. One possible scenario may be that this retrovirus was introduced into the great ape lineages by horizontal transmission, perhaps from contact with an ancient Old World monkey species.

Our data support a model where ancestral chimpanzee and gorilla species were infected independently and contemporaneously by an exogenous source of gammaretrovirus 3–4 million years ago. While similar infections with a related retrovirus appear commonplace among the Old World monkeys, contemporary human and orangutan populations show no molecular vestiges of this infection (see Figure 2). The molecular basis for this historical difference is unclear. While geographic isolation of the African and Asian ape lineages during the Miocene [30,31] might account for part of this difference, the ancestral habitat of early hominids is generally thought to have overlapped, in part, with the African apes [32,33]. Furthermore, both Asian (macaque) and African (baboon) Old World monkeys show evidence of PTERV1 proviral integrations less than 2 million years ago, indicating that the exogenous source virus is either endemic to both continents or that ancestral populations frequented both continents.

Several speculative scenarios may be envisioned to explain the absence of retrovirus in both the orangutan and human lineages. It is possible that the African apes evolved a susceptibility, or humans and Asian apes developed resistance to infection, although in either scenario convergent evolution would have had to have occurred with respect to the viral infections. Studies of the retroviral infection of the Lake Casitas mouse population reveal that such susceptibility/resistance genes may emerge very quickly among closely related strains of mice [34]. Another scenario may be that the lineage that ultimately gave rise to humans did not occupy the same habitat as the ancestral chimpanzee and gorilla lineages. An excursion by early hominids to Eurasia during the time that PTERV1 infected African great apes and then a return to Africa would explain this phylogenetic inconsistency. It is also possible that this effect may have been created by dramatic differences in ancestral population structure. If, for example, the ancestral populations of humans and orangutans were substantially larger than those of the African great apes, the fixation of new insertions (1/2*N*) would occur much more rapidly within small inbred populations even if similar infection rates existed. A similar model has recently been proposed, albeit in the opposite direction, to explain an increase of “apparent” Alu Ya5 and Yb8 retroposition activity in the human lineage but not in chimpanzees and gorillas [35]. In this regard, it is interesting that documented differences in the patterns of endogenous retrovirus between domesticated and feral species have been attributed to inbreeding [19,20]. There is, however, no evidence to date that the ancestral populations of chimpanzees were smaller than that of humans. Recent studies suggest that ancestral chimpanzee populations, in fact, may have been two to four times larger [36,37] than the effective human population size (greater than 10,000). A dramatic population crash in ancestral gorilla and chimpanzee populations would be required to explain the effect we have observed. Further population genetic studies of contemporary great apes or paleoanthropological work may help to eliminate these and other possible scenarios.

Finally, it is not unreasonable to assume that these ancient infections reduced effective population size if fitness of ancestral populations were compromised by the infection. Recently, such an ancient retroviral infection was predicted to have occurred in chimpanzee based on a completely separate line of reasoning. De Groot and colleagues reported a dramatic reduction of genetic variability of intronic sequence from the major histocompatibility complex (MHC) I human leukocyte antigen loci (A, B, and C) among chimpanzee when compared to human populations [38]. This is a notable exception to other studies that demonstrate 2- to 4-fold greater diversity in chimpanzee populations than in humans [39]. Based on the evolutionary age of some of the new lineages and comparisons between chimpanzee and bonobo, de Groot and colleagues estimated the loss of MHC I diversity to have occurred before subspeciation, 2–3 million years ago. Due to the central role that pathogens play in eliciting immune response against viral infections and the fact that high-frequency MHC I haplotypes target conserved epitopes of the HIV-1 virus, the authors speculated that the unusual pattern of MHC I diversity might be the result of a pandemic retroviral infection that positively selected a small number of lineages to be swept through the population. Our findings are consistent with the timing of this loss of diversity and may represent the genomic vestiges of this retroviral pandemic.

Due to its integration into the germline, this retroviral infection, thus, may have had a double impact. At a genetic level, at least 5% of the retroviral insertions would have resulted in lethality when homozygosed [22]. The 3-fold integration bias against gene insertions may represent a strong signature of this selection. This distribution is in sharp contrast to patterns of somatic retroviral infection [26,27] as well as recent class II human endogenous retroviral elements that map near or within genes [9]. In a background of reduced survival and lowered fecundity, genetic bottlenecks may have been frequent occurrences among ancestral chimpanzee and gorilla populations after speciation [33]. During this time of retroviral crisis, small subsets of retrovirus-induced mutations may have been fixed at an increased frequency. The mutation and fixation of multiple weakly deleterious mutations could, in theory, promote further saltatory and irrevocable changes in phenotypic traits among these progenitor populations. Such episodic mutational events may have simultaneously propelled species differentiation and cemented reproductive barriers between humans and the African great apes. In such a scenario greater sequence divergence over these regions might be expected because of a lack of introgression upon secondary contact among incipient species. It will be interesting to compare patterns of divergence for these sites with those for other genomic regions in humans and African great apes when genome sequence of sufficient quality becomes available.

## Materials and Methods

### 

#### Chimpanzee genome analysis

A computational pipeline was established to identify insertions and deletions between contiguous BAC chimpanzee genome sequence and the human genome assembly (EEE, unpublished data). Using alignment visualization software (PARASIGHT), we detected an 8.45-kb segment present in chimpanzee (AC097267 from 91,434 to 99,886) but absent in human. The sequence was not classified as a repeat (RepeatMasker, version 4.0) but six-frame translation and protein BLAST sequence similarity searches showed significant homology to retroviral sequences including *gag, pol,* and *env* proteins (see Figure 1). This family of repeat elements, PTERV1, corresponds to the largest of three retroviral repeat families that were discovered in chimpanzee genome sequence but not in humans (Chimpanzee Genome Sequencing Consortium, unpublished data). Sequence analysis of the chimpanzee genome (November 2003) identified 107 putative full-length copies and 90 solo LTR elements (see Table S7). Since most full-length copies were incompletely assembled, map positions were confirmed by paired end-sequence analysis (see below) in addition to BLAST sequence similarity searches.

#### Genomic hybridization

Primate DNA was restriction-enzyme-digested, transferred to nylon membrane, and hybridized as described previously [40]. Species included human *(Homo sapiens),* common chimpanzee *(Pan troglodytes),* bonobo *(Pan paniscus),* gorilla *(Gorilla gorilla),* orangutan *(Pongo pygmaeus),* siamang *(Hylobates syndactylus),* white-handed gibbon *(Hylobates lar),* Abyssinian black-and-white colobus monkey *(Colobus guereza),* olive baboon *(Papio anubis),* rhesus macaque *(Macaca mulatta),* and Japanese macaque *(Macaca fuscata)* (see Figure 2). In addition, we analyzed multiple individuals (three or four) from each ape lineage and a diversity panel of 16 humans and nine baboons. PCR-amplified products (see Table S1 for PCR oligonucleotide sequence and conditions) corresponding to the *gag, env,* and LTR portions of PTERV1 were used as radioactive probes as described [41]. Large-insert genomic BAC libraries from chimpanzee (RPCI-43), gorilla (CHORI-255), the olive baboon (RPCI-41), and the rhesus macaque (CHORI-250) were also hybridized. A total of 2,706 BAC clones were obtained that hybridized with PTERV1 *gag*, *env,* and LTR probes, while 6,032 clones hybridized solely with the LTR probe (see Table 1). The former were classified as full-length insertions of PTERV1, while the latter was classified as solo LTR elements.

#### Sequencing.

A total of 1,467 BAC clones containing full-length insertions were end-sequenced and are publicly available from GenBank. In addition, a total of 101 genomic clones (42 chimp, 25 gorilla, 20 baboon, and 14 macaque) corresponding to distinct insertion loci were comparatively sequenced from *gag* and *env* portions of the endogenous retrovirus (approximately 823 bp each). Individual clones were also subjected to LTR sequencing, and sequencing variants were analyzed using PolyPhred software [42]. All PCR products (forward and reverse reactions) were directly sequenced, using a modified dye terminator sequencing protocol [41]. Fluorescent traces were analyzed using an Applied Biosystems PRISM 3100 DNA Sequencing System (Perkin-Elmer Applied Biosystems, Norwalk, Connecticut, United States), and the quality of the sequence data was assessed with Phred/Phrap/Consed software [43,44].

#### Expression analysis

Gene expression differences between human and chimpanzee were assessed as previously described [28] with the following exceptions: five tissues (heart, brain, liver, testis, and kidney) were compared among five chimpanzee and six human samples using Affymetrix (Santa Clara, California, United States) HG U133plus2 arrays. Only those oligonucleotide probe sets that showed a perfect match between human and chimpanzee genomic sequence DNA were considered in this analysis (17,617/54,377). Differentially expressed genes were defined as those that met the following criteria: (i) the gene had to be expressed in all individuals from at least one species (detection *p-*value of less than 0.065); (ii) the gene had to show a change in expression in the same direction (change *p*-value [two-tailed] of less than 0.5 or greater than 0.5) in all 30 pairwise comparisons; (iii) different probe sets from the same gene did not conflict. The full dataset that we analyzed consisted of a total of 17,617 probes corresponding to 7,947 genes that showed significant levels of expression in both chimpanzee and human. About 70% of the genes (5,488) showed equivalent levels of expression between both species, while approximately 30% (2,459) showed differential patterns of expression. We identified 14 genes in which retroviral insertions had occurred within the chimpanzee lineage. Ten of these were found in the full expression dataset: four genes showed equivalent expression levels, while six showed differential patterns (largely decreases) in expression. To determine whether this 2-fold increase might be significant, we performed a simulation study. We randomly sampled ten genes from the full dataset (7,947) and calculated the number of genes for which significant differences were observed for each replicate. We repeated this 10,000 times and determined that 9,511 of the replicates showed less than six differentially expressed genes, 374 showed precisely six differentially expressed genes, and 115 showed more than six. Based on this analysis of the control dataset, we determined that enrichment was weakly significant (*p* = 0.0489).

#### Evolutionary analyses

End sequences generated from BAC clone inserts (T7 and SP6) were used to position retroviral insertions with respect to the human genome reference (Build 34). We considered only optimal placements after sequence quality rescoring (Phred *Q* > 25) and masking of common repeats (at least 50 bp of unmasked sequence was required to place an end sequence). We required two independent BAC clones per locus, which allowed mapping intervals to be refined and eliminated potential false positives. A total of 275/287 locations mapped to independent locations. Twelve locations could not be distinguished based on the resolving power of this mapping approach (100–150 kb). Estimates of genetic distance (pairwise deletion) were calculated using Kimura's two-parameter model (where *s*/*v* was approximately 2.0) [45]. The elevated substitution rate for PTERV1 is consistent with the total number of substitutions (*K*_total_) being partitioned between a viral component (*K*_v_) and a nuclear component (*K*_n_). For protein-encoding portions of the retrovirus, the average number of synonymous (*K*_s_) and nonsynonymous (*K*_a_) substitutions per site was estimated using the modified Nei–Gojobori method [46]. We calculated evolutionary times of retroviral insertion based on the divergence of LTR flanks (0.13% divergence per million years and *r* = *K*/2*T*) [8]. Phylogenetic trees of multiple aligned sequences (ClustalW) were generated using neighbor-joining distance estimates (MEGA2). Only bootstrap values greater than 80% are indicated in the tree topology (see Figure 4). We modeled the genic distribution of retroviral insertions by random simulation of 107 full-length map positions within the human genome reference sequence. An insertion was classified as intronic if the insertion site mapped within the transcription start and transcription end of a Refseq gene annotation (920 Mb). By this measure, 29.7% (with gaps) and 32.1% (without gaps) of the genome is transcribed. Not once in 10,000 replicates was the measured gene distribution (14/107) observed. Similarly, we estimated the probability that six of the ten genes that showed significant expression differences between human and chimpanzee would have occurred by random chance.

## Supporting Information

Figure S1 Southern Analysis of PTERV 1 among Primates A panel of African great ape DNAs is compared for both the *gag* probe number 1 by PstI and PvuII restriction enzyme digest. Proximity of probe number 1 to the VNTR sequence and its variable copy number allows the detection of hundreds of insertion sites, showing limited intraspecific variation. Species represented include human (HSA), common chimpanzee (PTR), bonobo (PPA), gorilla (GGO), and orangutan (PPY). Below each panel, a restriction map (chimpanzee reference sequence AC097267) is presented in relation to the hybridization probe: PstI (closed circles), PvuII (open circles), and HpaII/MspI (triangles).
(475 KB PDF).

Figure S2 Methylation Status of PTERV1 Sequence Methylation status of the retroviral insertions is compared between peripheral blood DNA and transformed lymphoblast cell lines for baboon (PHA), chimpanzee (PTR), and gorilla (GGO). HpaII does not digest methylated restriction enzyme sites, while MspI is insensitive. The differential patterns suggest most PTERV1 insertions are methylated.
(317 KB PDF).

Figure S3 Ambiguous Overlap Loci and Primate Phylogeny The Distribution of Ambiguous Overlap Loci for Human and Great Apes Is Shown (Arcs) with Respect to a Generally Accepted Phylogeny of Catarrhine Primates
If these sites are orthologous, then at least six deletion events would have had to occur independently in orangutan and human at precisely the same positions in both genomes.
(10 KB PDF).

Figure S4 Resolution of Ambiguous Overlap Locus (RP43–114h16) (A) Multiple alignment of sequence flanking chimp insertion RP43–114h16 against chimpanzee whole-genome shotgun sequence reads. Precise site of retrovirus integration is indicated by the red arrow, and a 5-bp target site duplication is indicated in the blue box. Note that no chimpanzee sequence reads are contiguous across the region because of the 6-kb retrovirus insertion that extends from the insertion site (data not shown).
(B) Sequence similarity search of sequence flanking chimp insertion against macaque whole-genome shotgun sequence reads. The analysis shows that the chimpanzee insertion site is not shared with macaque. In contrast, human and macaque sequence are contiguous across this site. A macaque retroviral insertion maps to a different location within this 160-kb interval of the genome. This overlap locus is, therefore, resolved as non-orthologous.
(33 KB PDF).

Figure S5 Resolution of Ambiguous Overlap Locus (RP43–151j13) (A) Multiple alignment of sequence flanking chimp retroviral insertion RP43–151j13 against chimpanzee whole-genome shotgun sequence reads. Precise site of retrovirus integration is indicated by the red arrow, and a 4-bp target site duplication is indicated by the blue box. Note that no chimpanzee sequence reads are contiguous across the region because of the 6-kb retrovirus insertion that extends from the insertion site (data not shown).
(B) Sequence similarity search of same sequence flanking chimp insertion against macaque whole-genome shotgun sequence reads. The analysis shows that the chimpanzee insertion site is not shared with macaque. Human and macaque sequences are contiguous across the chimpanzee insertion site. A macaque retroviral insertion maps to a different location in this 160-kb region of the genome. This overlap locus is, therefore, resolved as non-orthologous.
(34 KB PDF).

Figure S6 PTERV1 Phylogenetic Trees Neighbor-joining phylogenetic trees were constructed for (A) *env,* (B) *gag,* and (C) LTR portions of the retrovirus independently, as described in Figure 4. Bootstrap support (*n* = 10,000 replicates) for individual branches is indicated in italics and is substantially lower owing to the limited number of basepairs compared within each tree (especially in Figure S6B). The general topology of each of the three trees is comparable and shows gorilla and chimpanzee retroviral insertions as a single monophyletic clade.
(47 KB PDF).

Figure S7 A Random Simulation of 107 Retroviral Insertions within the Human Genome Genic space was determined as the distance encompassed from transcription start to transcription end of Refseq gene annotations (920 Mb). By this measure, 29.7% (with gaps) and 32.1% (without gaps) of the genome is transcribed. Not once in 10,000 replicates was the measured gene distribution (14/107) observed.
(13 KB PDF).

Table S1 PCR Primers and Probes (11 KB PDF).

Table S2 Mapping of Full-Length Retroviral Insertion Sites on Human Genome (Build 34) (760 KB PDF).

Table S3 Retroviral Map Intervals That Potentially Overlap between Species (50 KB PDF).

Table S4 Inter- and Intra-Specific Genetic Distance Estimation among PTERV1 Elements (A) Genetic distance for *gag–env* portion.
(B) Genetic distance for LTR portion.
(13 KB PDF).

Table S5 Distribution of LTR Mismatches for Each Species (Observed) Compared with a Poisson Distribution (Expected) (24 KB PDF).

Table S6 Mapping of Solo LTR and Full-Length PTERV1 Elements on Human Genome (Build 34) (66 KB PDF).

Table S7 PTERV1 Distribution in Chimpanzee Genome (9 KB PDF).

Table S8 The Keys (Numbers) and Their Corresponding Clones for Figure 4
(24 KB PDF).

### Accession Numbers

The sequence data described in this paper have been submitted to GenBank (http://www.ncbi.nlm.nih.gov/Genbank/) under accession numbers AY758600–AY760022, AY760471–AY760631, AY760098–AY760470, and AY760640–AY761013.

The GenBank accession numbers for the sequences discussed in this paper are baboon endogenous retrovirus (AF142988), feline leukemia virus (AAC31801), murine leukemia virus (AAA79427), porcine endogenous retrovirus type C (CAC39617), and chimpanzee BAC basepair positions 91,434–99,886 (AC097267).

## Abbreviations

LTR: long terminal repeat
MHC: major histocompatibility complex
PTERV1: Pan troglodytes endogenous retrovirus 1
