# Supplementary material for: Lineage-Specific Expansions of Retroviral Insertions within the Genomes of African Great Apes but Not Humans and Orangutans
Source: PLoS Biol. 2005 Mar 1;3(4):e110. doi: 10.1371/journal.pbio.0030110 (PMC1054887; doi:10.1371/journal.pbio.0030110)
Supplement: Supplementary file 1 [file pbio.0030110.sg001.pdf]

Figure S1

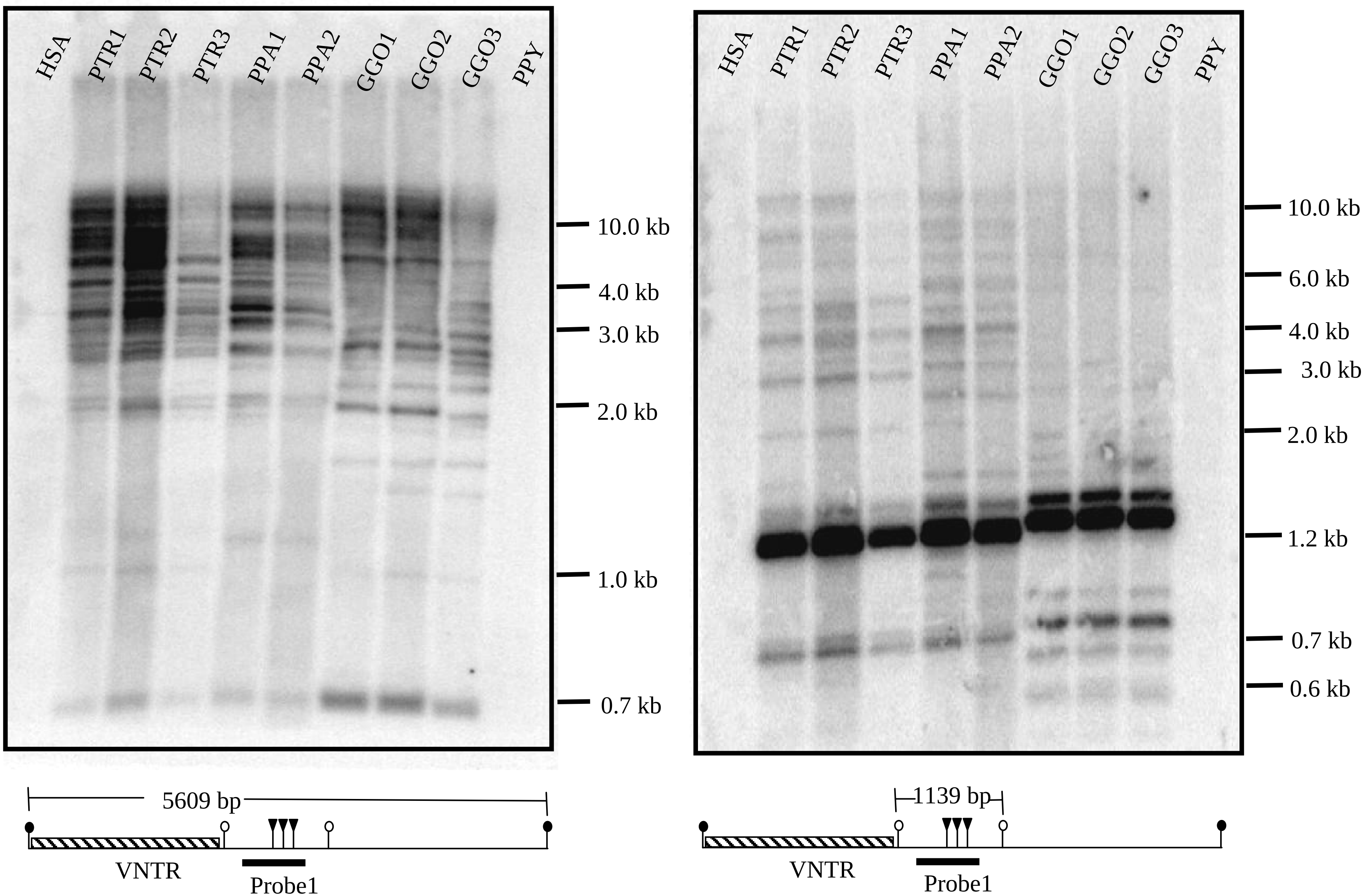
HSA
PTR1
PTR2
PTR3
PPA1
PPA2
GGO1
GGO2
GGO3
PPY
10.0 kb
4.0 kb
3.0 kb
2.0 kb
1.0 kb
0.7 kb
5609 bp
VNTR
Probe1
HSA
PTR1
PTR2
PTR3
PPA1
PPA2
GGO1
GGO2
GGO3
PPY
10.0 kb
6.0 kb
4.0 kb
3.0 kb
2.0 kb
1.2 kb
0.7 kb
0.6 kb
1139 bp
VNTR
Probe1
