# Supplementary material for: Lineage-Specific Expansions of Retroviral Insertions within the Genomes of African Great Apes but Not Humans and Orangutans
Source: PLoS Biol. 2005 Mar 1;3(4):e110. doi: 10.1371/journal.pbio.0030110 (PMC1054887; doi:10.1371/journal.pbio.0030110)
Supplement: Supplementary file 2 [file pbio.0030110.sg002.pdf]

Figure S2

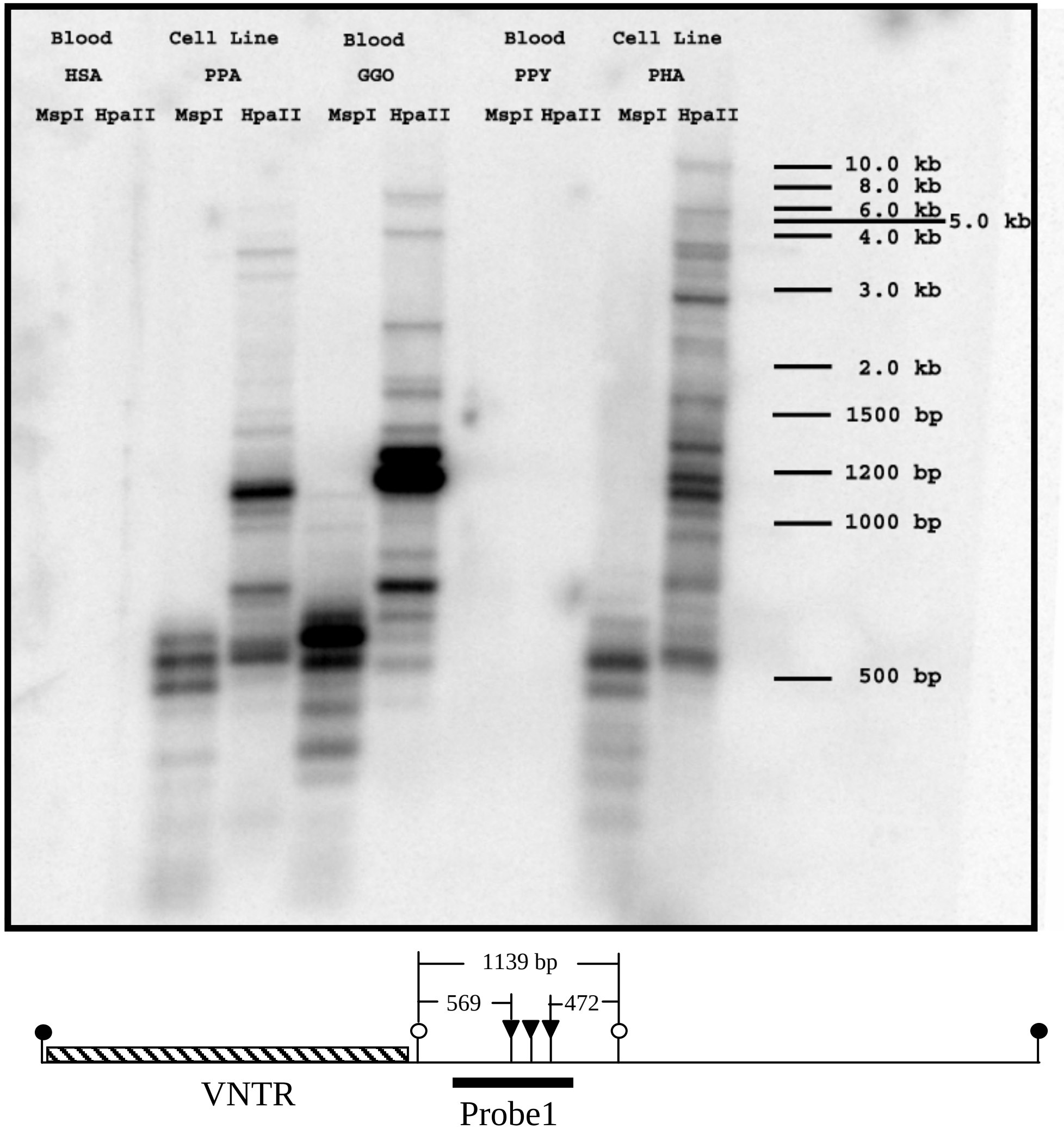
Blood
HSA
Cell Line
PPA
Blood
GGO
Blood
PPY
Cell Line
PHA
MspI HpaII MspI HpaII MspI HpaII MspI HpaII MspI HpaII
10.0 kb
8.0 kb
6.0 kb
5.0 kb
4.0 kb
3.0 kb
2.0 kb
1500 bp
1200 bp
1000 bp
500 bp
1139 bp
569
472
VNTR
Probe1
