# Supplementary material for: Lineage-Specific Expansions of Retroviral Insertions within the Genomes of African Great Apes but Not Humans and Orangutans
Source: PLoS Biol. 2005 Mar 1;3(4):e110. doi: 10.1371/journal.pbio.0030110 (PMC1054887; doi:10.1371/journal.pbio.0030110)
Supplement: Supplementary file 3 [file pbio.0030110.sg003.pdf]

Figure S3

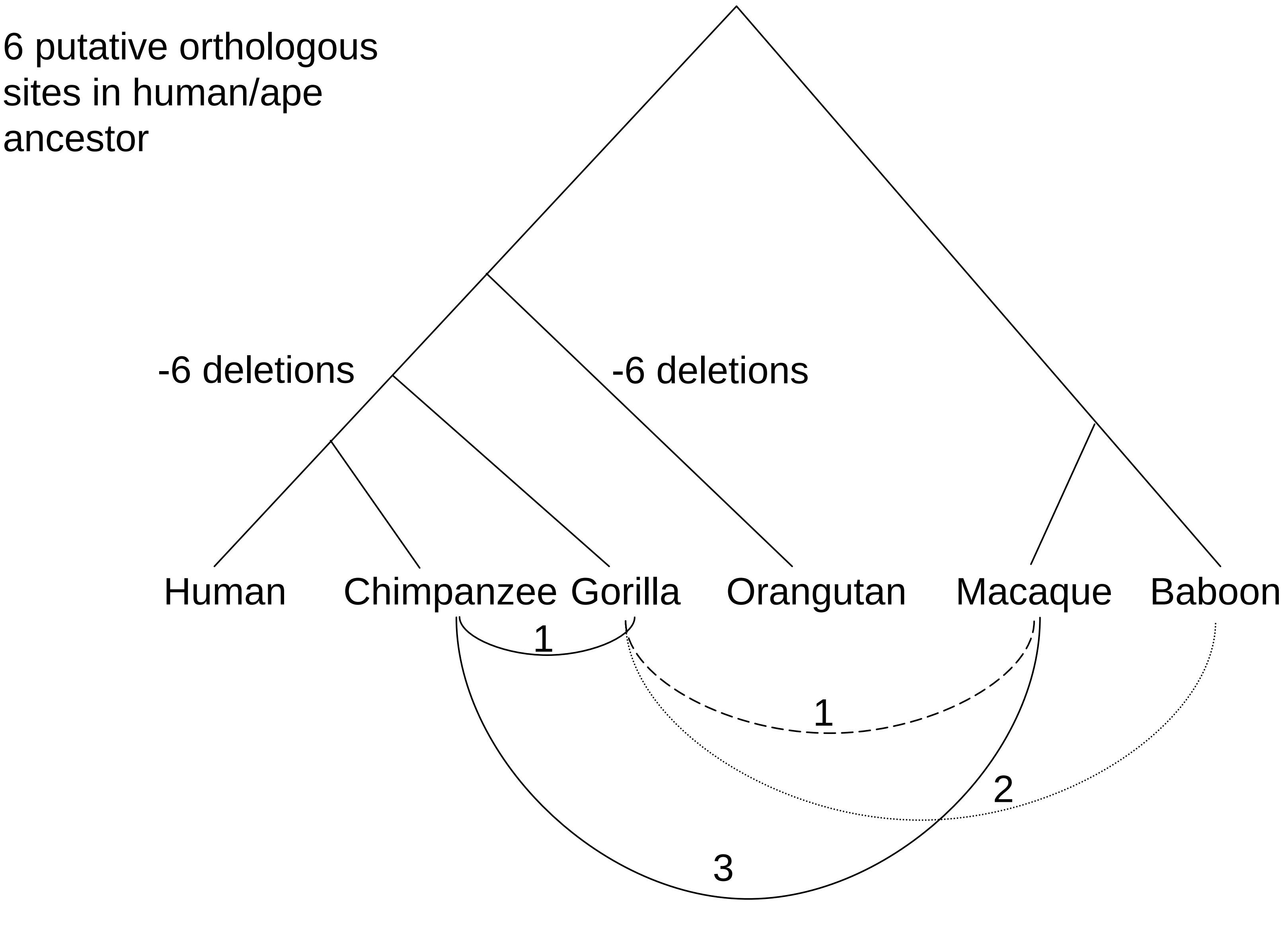
6 putative orthologous sites in human/ape ancestor
-6 deletions
-6 deletions
Human
Chimpanzee
Gorilla
Orangutan
Macaque
Baboon
1
1
2
3
