# Supplementary material for: Lineage-Specific Expansions of Retroviral Insertions within the Genomes of African Great Apes but Not Humans and Orangutans
Source: PLoS Biol. 2005 Mar 1;3(4):e110. doi: 10.1371/journal.pbio.0030110 (PMC1054887; doi:10.1371/journal.pbio.0030110)
Supplement: Supplementary file 4 [file pbio.0030110.sg004.pdf]

Figure S4 A

```
QUERY                   1921 tggtattgatagcgctcttcacagagttaggagaattacgtgaattgatacatgaataac 1980
G591P638132FH11.T0.888  480  .......................................a.................... 539
G591P619427FB10.T0.896  454  .......................................a.................... 513
utj19c10.g1.1109        483  .......................................a.................... 424
G591P637088FC7.T0.926   203  .......................................a.................... 262
G591P633383FH7.T0.867   176  .......................................a.................... 235
qxu66h04.g1.1270        168  ......................t................a.................... 227
qgn04f12.b1.739         85   .......................................a.................... 144

QUERY                   1981 acttagaacagaataagacctcactggacattaataggtgactccttggctttttgctgt 2040
G591P629550RC10.T0.906  857                ..................a............................ 812
G591P638132FH11.T0.888  540  ...................                                          558
G591P619427FB10.T0.896  514  ...................                                          532
utj19c10.g1.1109        423  ...................                                          405
G591P613127RG8.T0.932   323                ..............................................  368
qqg38d12.b1.917         538                ..............................................  583
uqz98h11.b1.1101        367                ..............................................  322
G591P637088FC7.T0.926   263  ...................                                          281
G591P633383FH7.T0.867   236  ...................                                          254
G591P604566FD3.T0.1042  250                ..............................................  205
qxu66h04.g1.1270        228  ...................                                          246
qgn04f12.b1.739         145  ...................                                          163

QUERY                   2041 ggctgttgtcactacatgcatgtctgcctcaccttctggcttcctctccaactgccgccc 2100
G591P629550RC10.T0.906  811  ......g.........................-....a....t................. 753
G591P613127RG8.T0.932   369  ............................................................ 428
qqg38d12.b1.917         584  ............................................................ 643
uqz98h11.b1.1101        321  ............................................................ 262
G591P604566FD3.T0.1042  204  ............................................................ 145
```

Figure S4 B

```
QUERY                     1923 gtattgatagcgctcttcacagagttaggagaattacgtgaattgatacatgaataacac 1982
LAWNP177769.R.1009        94   ..........t..........................a..................g... 153
LAWRP69854.R.1112         649  ..........t..........................a..................g... 708
LAWNP25141.F.922          512  ..........t..........................a..................g... 571
LAWEP977902.R.847         769                                         ........c.c......c... 749
MQAA-aes90g12.g1.660      520  ..........t..........................a..................g... 579
zge70g08.g1.873           607  ..........t..........................a..................g... 548

QUERY                     1983 ttagaacagaataagacctcactggacattaataggtgactccttggctttttgctgtgg 2042
LAWNP177769.R.1009        154  ..................................................c......... 213
LAWRP69854.R.1112         709  ..................................................c......... 768
LAWNQ1106737.R.1188       946                                                      ........ 939
LAWNP25141.F.922          572  ..................................................c......... 631
LAWEP977902.R.847         748  ..g..c.......g.c.............a..............g.....c....g.... 689
MQAA-aes90g12.g1.660      580  ..................................................c......... 639
zge70g08.g1.873           547  ..................................................c......... 488

QUERY                     2043 ctgttgtcactacatgcatgtctgcctcaccttctggcttcctctccaactgccgccccc 2102
LAWEP668057.F.1327        1068                                       .....t...........t.... 1047
LAWNP177769.R.1009        214  ...................................a...................t.... 273
LAWRP69854.R.1112         769  ...................................a...................t.... 828
LAWNQ1106737.R.1188       938  ....-.........................a....a...................t.... 880
LAWNP25141.F.922          632  ...................................a...................t.... 691
LAWEP977902.R.847         688  ........c..........................a...................t.... 629
MQAA-aes90g12.g1.660      640  .....................                                        660
zge70g08.g1.873           487  ...................................a...................t.... 428
```
