# Supplementary material for: Lineage-Specific Expansions of Retroviral Insertions within the Genomes of African Great Apes but Not Humans and Orangutans
Source: PLoS Biol. 2005 Mar 1;3(4):e110. doi: 10.1371/journal.pbio.0030110 (PMC1054887; doi:10.1371/journal.pbio.0030110)
Supplement: Supplementary file 5 [file pbio.0030110.sg005.pdf]

Figure S5 A

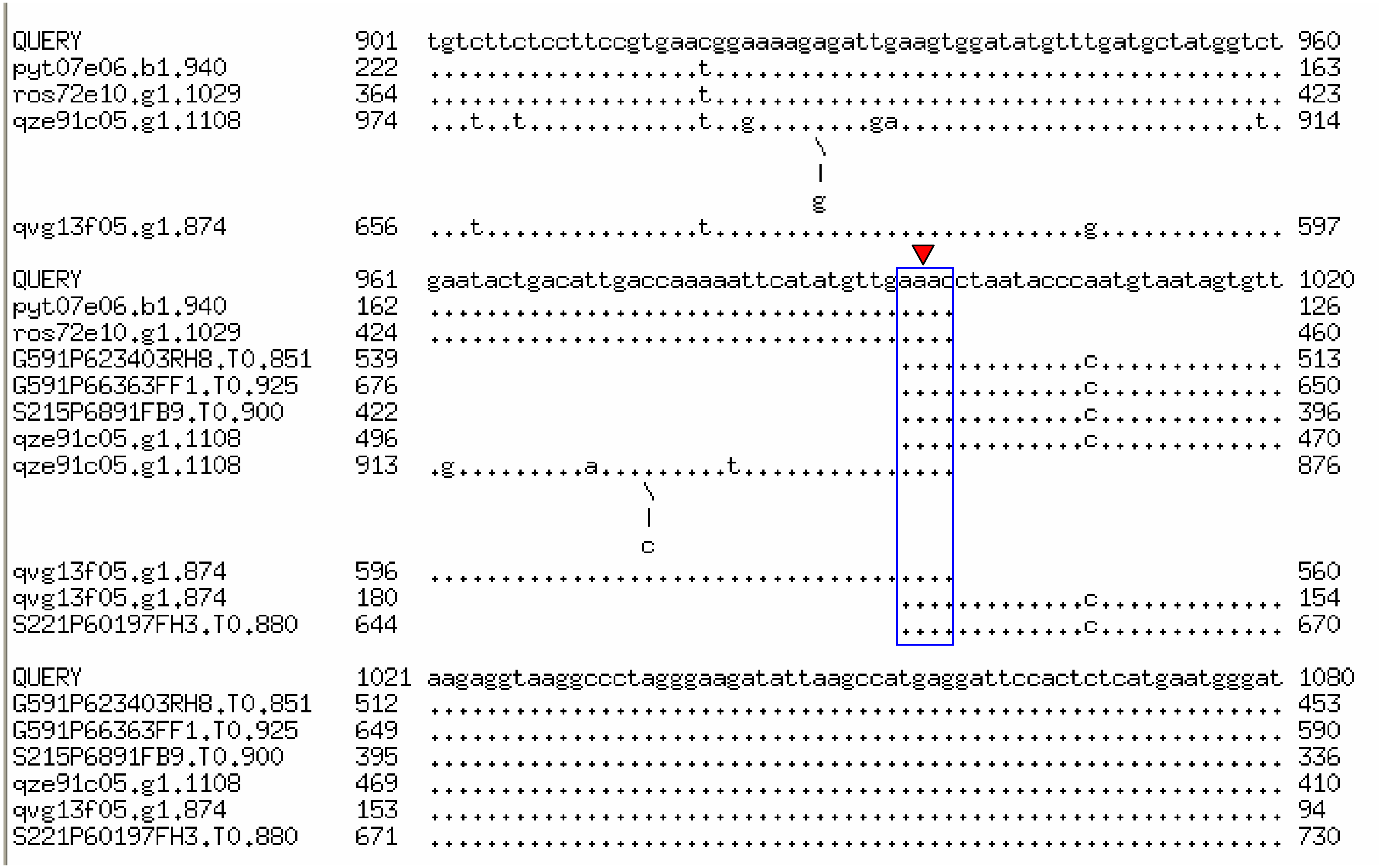
QUERY 901 tgtcttctccttccgtgaacggaaaagagattgaagtggatatgtttgatgctatggtct 960
pyt07e06.b1.940 222 ....t.. 163
ros72e10.g1.1029 364 ....t.. 423
qze91c05.g1.1108 974 ...t..t............t..g........ga.........................t. 914
\
|
g
qvg13f05.g1.874 656 ...t...............t..........................g............. 597
QUERY 961 gaatactgacattgaccaaaaattcatatgttgaaacctaatacccaatgtaatagtgtt 1020
pyt07e06.b1.940 162 126
ros72e10.g1.1029 424 460
G591P623403RH8.T0.851 539 ....c............. 513
G591P66363FF1.T0.925 676 ....c............. 650
S215P6891FB9.T0.900 422 ....c............. 396
qze91c05.g1.1108 496 ....c............. 470
qze91c05.g1.1108 913 .g.........a.........t............... 876
\
|
c
qvg13f05.g1.874 596 560
qvg13f05.g1.874 180 ....c............. 154
S221P60197FH3.T0.880 644 ....c............. 670
QUERY 1021 aagaggtaaggccctagggaagatattaagccatgaggattccactctcatgaatgggat 1080
G591P623403RH8.T0.851 512 453
G591P66363FF1.T0.925 649 590
S215P6891FB9.T0.900 395 336
qze91c05.g1.1108 469 410
qvg13f05.g1.874 153 94
S221P60197FH3.T0.880 671 730

Figure S5 B

```
QUERY                 901  tgtcttctccttccgtgaacggaaaagagattgaagtggatatgtttgatgctatggtct 960
RTCRL91TF.1076        558  .......c...g..a..g.t......t.........a....................... 499
LAWEP152726.R.1218    304  .......c...g..a..g.t......t.........a....................... 245
RHSKX29TR.1124        368  .......c...g..a..g.t......t.........a....................... 427
zad69g09.g1.1213      565  .......c...g..a..g.t......t.........a....................... 506
MQAA-aed14a06.b1.1213 117  .......c...g..a..g.t......t.........a.....a................. 58
wts56g12.g1.1502      400  .......c...g..a..g.t......t.........a....................... 459
wnc54c08.g1.858       375  .......c...g..a..g.t......t.........a....................... 434
LAWNP731990.R.879     217  .......c...g..a..g.t......t.........a....................... 276
LAWNQ1127079.R.1223   792  .......c...g..a..g.t......t.........a....................... 733
LAWNB1025.F.951       137  .......c...g..a..g.t......t.........a....................... 196
RHNIW77TF.909         893                      ......t.........a....................... 854
LAWNQ973601.R.1172    171  ..a....c...g..a..g.t...g..t........tac..a...........a....... 230

QUERY                 961  gaatactgacattgaccaaaaattcatatgttgaaacctaatacccaatgtaatagtgtt 1020
RTCRL91TF.1076        498  ........gt..............t..............................c.... 439
LAWEP152726.R.1218    244  ........gt..............t..............................c.... 185
RHSKX29TR.1124        428  ........gt..............t..............................c.... 487
zad69g09.g1.1213      505  ........gt..............t..............................c.... 446
MQAA-aed14a06.b1.1213 57   ........gt..............t......                              26
                                          \
                                          |
                                          c
wts56g12.g1.1502      460  ........gt..............t..............................c.... 519
wnc54c08.g1.858       435  ........gt..............t..............................c.... 494
LAWNP731990.R.879     277  ........gt..............t..............................c.... 336
LAWNQ1127079.R.1223   732  ........gt..............t...........t..................c.... 672
                                                                  \
                                                                  |
                                                                  g
LAWNB1025.F.951       197  ........gt..............t..............................c.... 256
RHNIW77TF.909         853  ........gt..............t..............................c.... 794
LAWNQ973601.R.1172    231  ........gt..............t..............................c.... 290

QUERY                 1021 aagaggtaaggccctagggaagatattaagccatgaggattccactctcatgaatgggat 1080
RTCRL91TF.1076        438  .....a.......t...................ca......................... 379
LAWEP152726.R.1218    184  .....a.......t...................ca......................... 125
RHSKX29TR.1124        488  .....a.......t...................ca......................... 547
zad69g09.g1.1213      445  .....a.......t...................ca......................... 386
```
