# Supplementary material for: Lineage-Specific Expansions of Retroviral Insertions within the Genomes of African Great Apes but Not Humans and Orangutans
Source: PLoS Biol. 2005 Mar 1;3(4):e110. doi: 10.1371/journal.pbio.0030110 (PMC1054887; doi:10.1371/journal.pbio.0030110)
Supplement: Supplementary file 6 [file pbio.0030110.sg006.pdf]

Figure S6 A

env

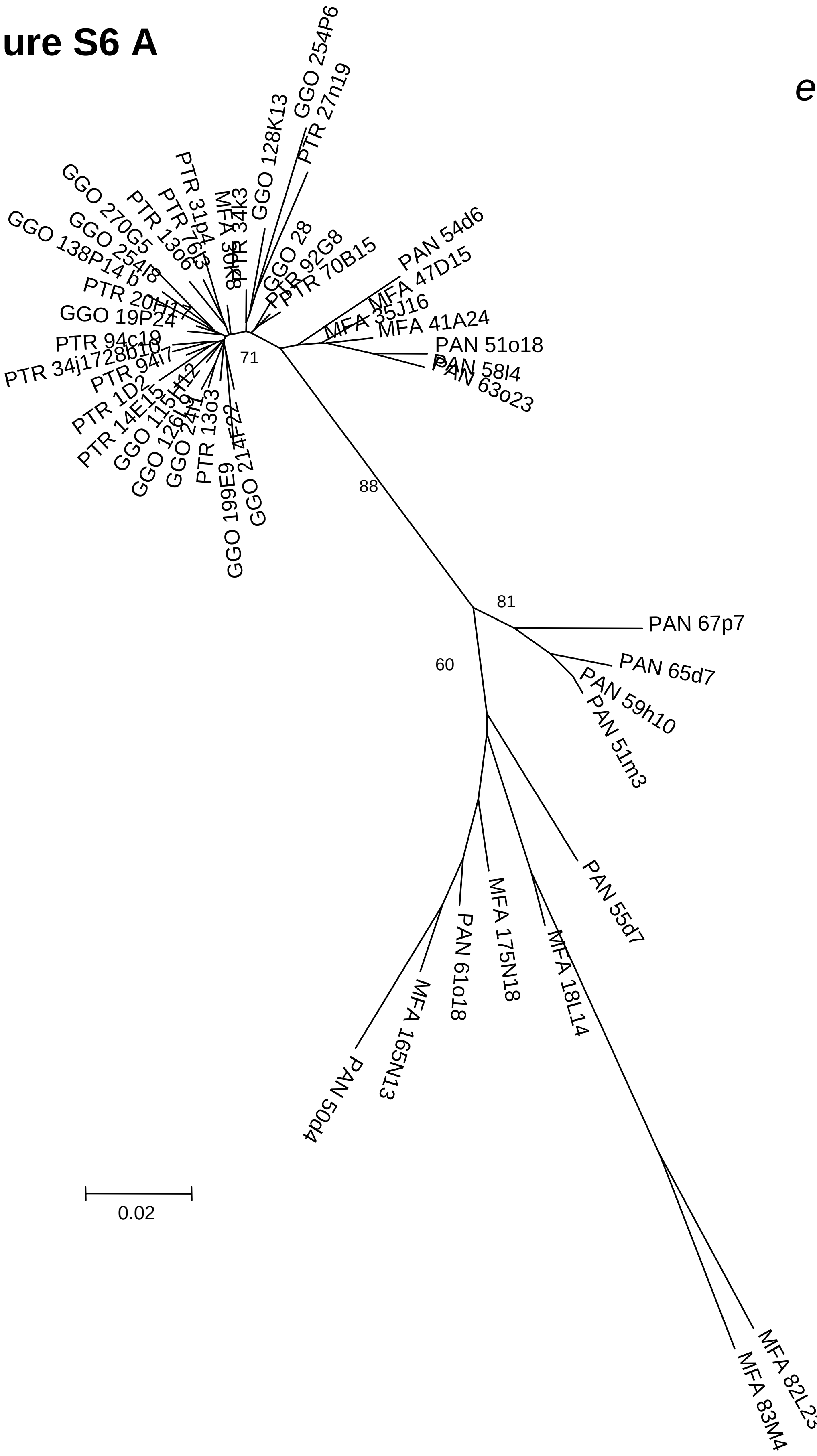

Figure S6 B

gag

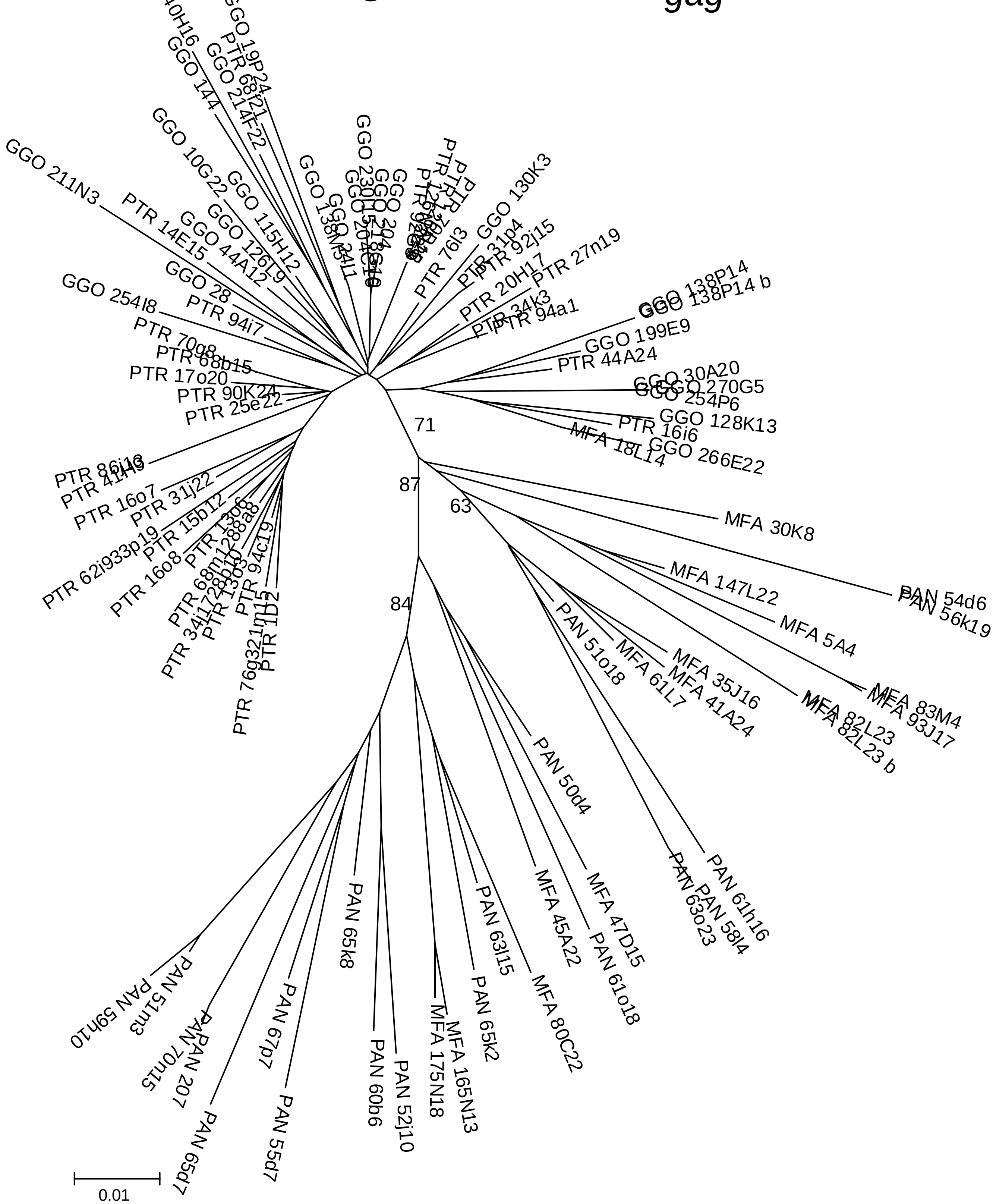

Figure S6 C

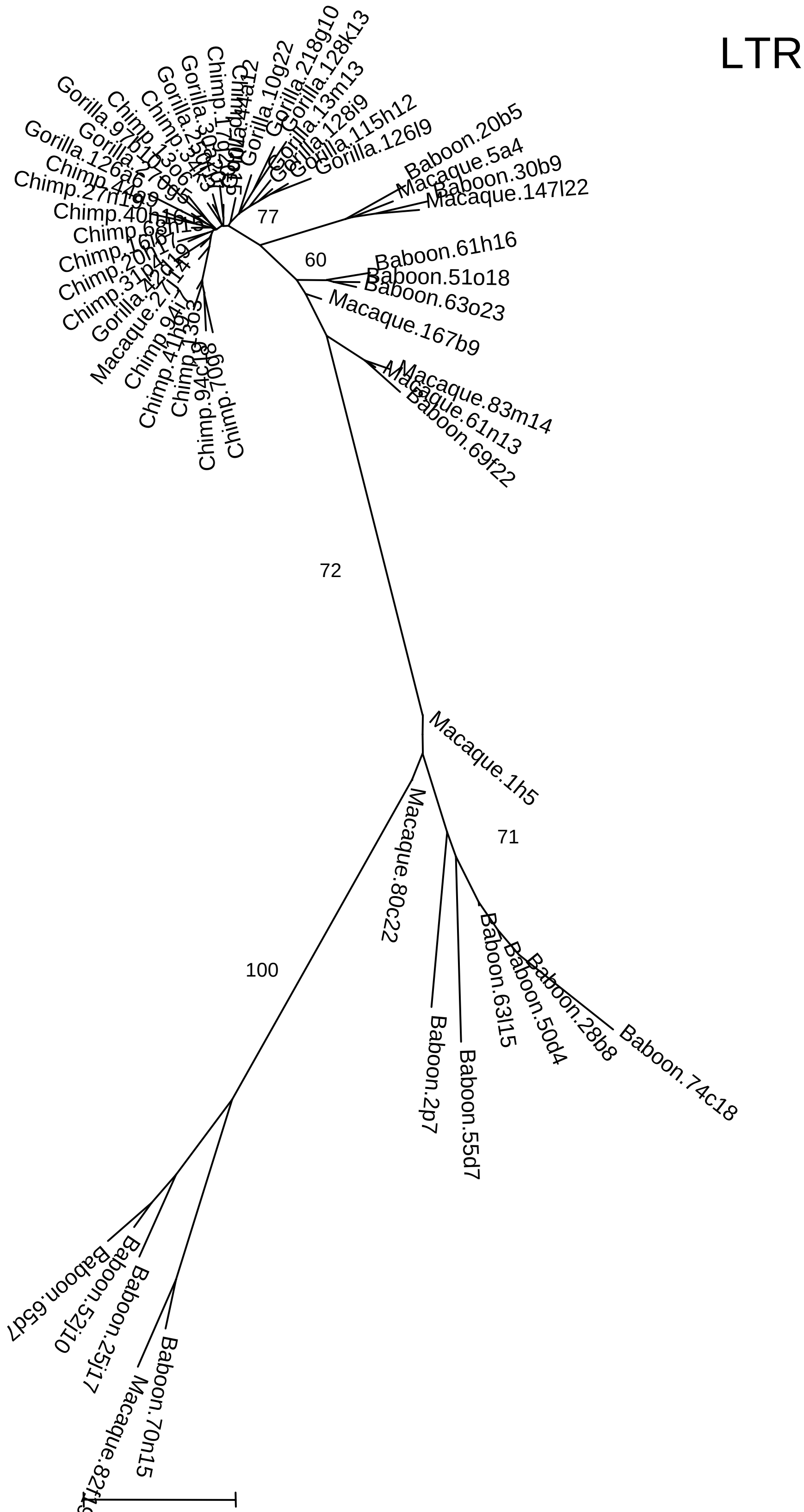
LTR
Gorilla.218g10
Gorilla.128k13
Gorilla.13m13
Gorilla.128i9
Gorilla.115h12
Gorilla.126l9
Gorilla.10g22
Chimp.27n19
Chimp.40h16
Baboon.20b5
Macaque.5a4
Baboon.30b9
Macaque.147l22
77
Baboon.61h16
60
Baboon.51o18
Baboon.63o23
Macaque.167b9
Macaque.83m14
Macaque.61n13
Baboon.69f22
Chimp.94c18
Chimp.70o8
72
Macaque.1h5
Macaque.80c22
71
Baboon.2p7
Baboon.55d7
Baboon.63l15
Baboon.50d4
Baboon.28b8
Baboon.74c18
100
Baboon.65d7
Baboon.52j10
Baboon.25j17
Baboon.70n15
Macaque.82f19
0.05
