# Supplementary material for: Lineage-Specific Expansions of Retroviral Insertions within the Genomes of African Great Apes but Not Humans and Orangutans
Source: PLoS Biol. 2005 Mar 1;3(4):e110. doi: 10.1371/journal.pbio.0030110 (PMC1054887; doi:10.1371/journal.pbio.0030110)
Supplement: Supplementary file 7 [file pbio.0030110.sg007.pdf]

# Figure S7

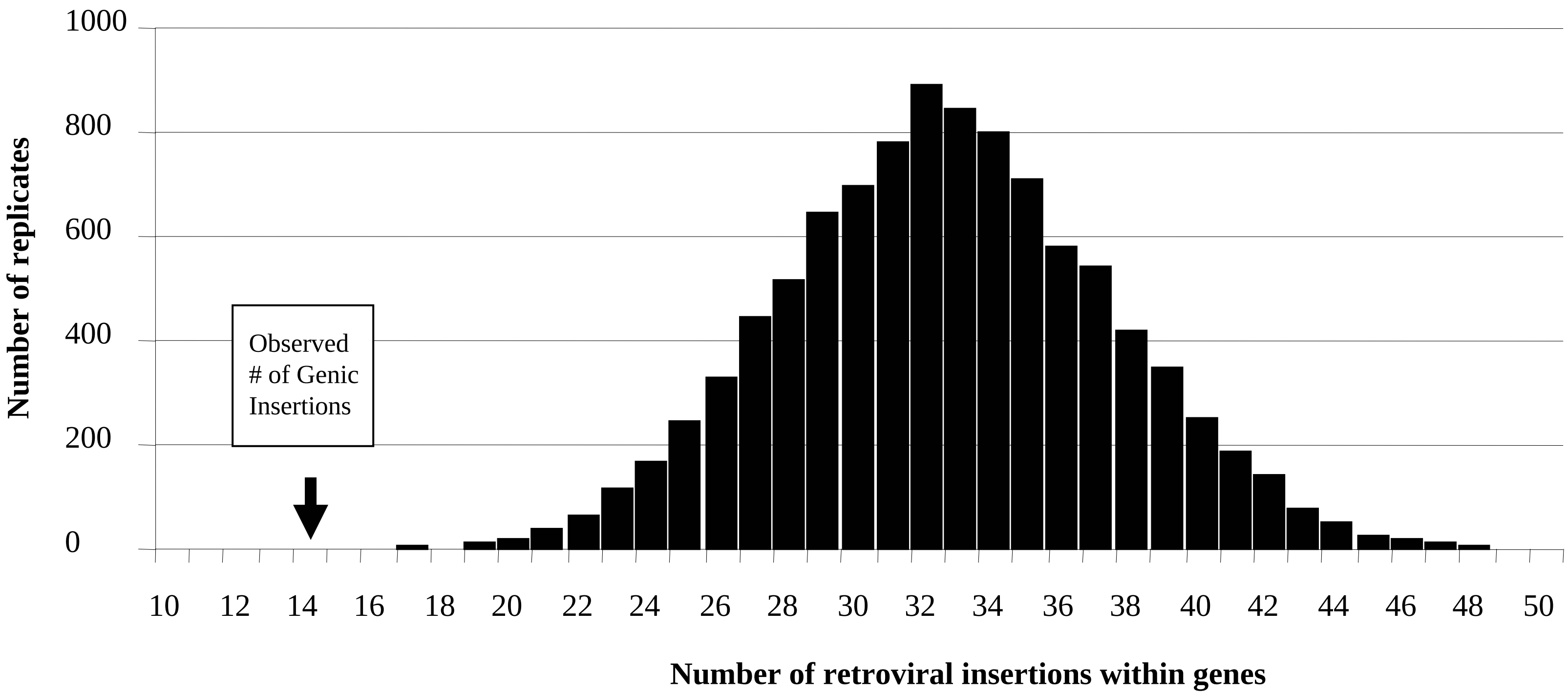
Number of replicates
1000
800
600
400
200
0
Observed
# of Genic
Insertions
10
12
14
16
18
20
22
24
26
28
30
32
34
36
38
40
42
44
46
48
50
Number of retroviral insertions within genes
