# Supplementary material for: Lineage-Specific Expansions of Retroviral Insertions within the Genomes of African Great Apes but Not Humans and Orangutans
Source: PLoS Biol. 2005 Mar 1;3(4):e110. doi: 10.1371/journal.pbio.0030110 (PMC1054887; doi:10.1371/journal.pbio.0030110)
Supplement: Supplementary file 8 [file pbio.0030110.st001.pdf]

Table S1 PCR primers and probes

Name	Oligonucleotide sequence
LTR1F	AACAAGCCCAGTACAAACACAT
LTR1R	GGAACTAACGGTCTGGGCATAACA
GAGF	TCGCTCTGAATGGCCTGCCTTTGA
GAGR	CGGGAAATAGGACCCCCAGATGAC
ENVF1	AGCCAGCTCCTAATCCCTTTGTCT
ENVF2	TAAGCCCGGGAAATTAGTGGCCAG
ENVR1	ATCCAGCGACAAGAAAACCGCCTG
ENVR2	AACTTGCCAACCTTACAGGACTTC
LTR2F	AACAAGCCCAGTACAAACACAT
LTR2R	GGAACTAACGGTCTGGGCATAACA

*Oligonucleotide sequence is based on reference sequence AC097267

**All oligonucleotides are 5' >> 3' orientation.

***Probe #1 is a 510 bp PCR amplicon generated by primers GAGF and GAGR, Probe #2 is a 343bp PCR amplicon generated by LTR1F and LTR1R, while Probe #3 is a 470 PCR amplicon generated by ENVF1 and ENFR2.
