# Supplementary material for: Lineage-Specific Expansions of Retroviral Insertions within the Genomes of African Great Apes but Not Humans and Orangutans
Source: PLoS Biol. 2005 Mar 1;3(4):e110. doi: 10.1371/journal.pbio.0030110 (PMC1054887; doi:10.1371/journal.pbio.0030110)
Supplement: Supplementary file 9 [file pbio.0030110.st002.pdf]

Table S2 Mapping of full length retroviral insertion sites on human genome (build34)

QNAME	QB	QE	QLEN	SNAME	SB	SE	OREINT	FractionBP
RP41_16i8TV.752	149	211	752	chr1	1371466	1371404	-	0.967213
RP43_0031I08.g1.658	83	348	658	chr1	2198861	2199126	+	0.984314
RP43_0031I08.b1.718	321	572	718	chr1	2371245	2370994	-	1
RP41_0074I19.b1.687	78	309	687	chr1	10269507	10269738	+	0.954545
CH255_132i21TV.645	1	124	645	chr1	13481845	13481968	+	0.988889
CH255_250l17TV.623	1	346	623	chr1	13497056	13497401	+	0.990826
CH255_97b10TJ.357	4	149	357	chr1	13620893	13621038	+	0.983051
CH255_250l17TJ.812	677	796	812	chr1	13658366	13658247	-	1
CH255_132i21TJ.842	383	802	842	chr1	13662925	13662506	-	0.981333
RP43_0148H12.g1.619	170	326	619	chr1	30177937	30177781	-	0.978873
RP41_8h9TJ.612	1	96	612	chr1	32457795	32457890	+	0.977273
RP41_2p7TV.842	697	749	842	chr1	45193610	45193662	+	0.954545
RP41_0057G22.b1.766	324	545	766	chr1	45264103	45263882	-	0.958333
RP41_20b5TJ.706	176	397	706	chr1	45264103	45263882	-	0.963964
RP41_2p7TJ.825	296	589	825	chr1	45338004	45337711	-	0.989796
RP41_36m15TJ.843	628	796	843	chr1	45348404	45348236	-	0.965986
CH255_218g10TJ.638	1	394	638	chr1	46784230	46784623	+	0.966767
CH255_218g10TV.531	7	530	531	chr1	46805250	46805773	+	0.987835
CH255_13m13TJ.218	1	214	218	chr1	46934606	46934819	+	0.990291
CH255_264k10TJ.844	572	844	844	chr1	46981395	46981123	-	0.990783
CH255_13m13TV.230	7	192	230	chr1	47212390	47212205	-	0.989899
RP41_19o9TV.857	208	349	857	chr1	50571884	50572025	+	0.985915
RP41_25j17TJ.779	169	415	779	chr1	50772046	50771800	-	0.96
RP43_0094M19.g1.601	205	512	601	chr1	54621418	54621725	+	0.980488
RP43_0094M19.b1.657	425	491	657	chr1	54808881	54808815	-	1
CH255_252k17TJ.770	1	600	770	chr1	66808015	66808614	+	0.991259
CH255_235p19TJ.634	315	403	634	chr1	66922122	66922034	-	1
RP41_0069F22.b1.729	125	191	729	chr1	70068097	70068163	+	0.969697
RP41_0078K11.b1.697	80	144	697	chr1	70171635	70171699	+	0.984615
RP41_0069F22.g1.694	65	148	694	chr1	70226095	70226012	-	1
RP41_0071M18.b1.726	513	575	726	chr1	78640618	78640680	+	0.9375
RP41_24k19TJ.850	405	632	850	chr1	78785325	78785098	-	0.938967
RP41_0071M18.g1.706	90	307	706	chr1	78826231	78826014	-	0.956522
CH255_218a4TV.763	1	50	763	chr1	82077018	82077067	+	1
CH255_19m21TJ.447	75	136	447	chr1	90790733	90790794	+	0.983051
CH255_10n18TV.187	19	187	187	chr1	90866383	90866551	+	0.991304
CH255_19m21TV.455	4	184	455	chr1	90984597	90984417	-	0.980892
CH255_10n18TJ.289	4	289	289	chr1	91087424	91087139	-	0.988439
CH255_235p10TJ.645	216	645	645	chr1	91128797	91128368	-	0.981073
CH250_18m4TJ.863	723	784	863	chr1	91324181	91324120	-	0.971429
CH255_204j16TV.297	1	222	297	chr1	91324728	91324949	+	0.993548
CH250_159g12TV.707	269	470	707	chr1	91343343	91343142	-	0.950495
CH250_27g9TV.414	45	269	414	chr1	91343848	91344072	+	0.969466
CH250_48i21TV.151	1	151	151	chr1	96430541	96430691	+	0.932584
RP41_0159H24.g1.651	375	465	651	chr1	101244028	101244118	+	1
RP41_0073C02.b1.746	552	644	746	chr1	101365741	101365833	+	1
RP41_0159H24.b1.740	188	314	740	chr1	101426629	101426503	-	0.944882
RP41_0073C02.g1.706	399	514	706	chr1	101533357	101533242	-	1
CH255_208i3TV.634	522	633	634	chr1	103716355	103716466	+	1

CH255_229l3TV.842	100	230	842	chr1	103922950	**103922820**	-	0.976744
CH255_114d2TJ.646	488	646	646	chr1	103941814	103941656	-	0.98
RP41_0155I15.b1.757	105	481	757	chr1	106349811	**106350187**	+	0.928302
RP41_0155I15.g1.750	385	470	750	chr1	106511667	**106511582**	-	0.944444
CH250_18m4TV.514	227	360	514	chr1	107865531	107865664	+	0.9875
RP43_0054C17.b1.700	61	345	700	chr1	111085105	**111085389**	+	0.972477
RP43_0054C17.g1.627	40	158	627	chr1	111253603	**111253485**	-	0.980769
RP43_0146F10.g1.624	4	509	624	chr1	113081765	113081260	-	0.987981
RP41_31a20TJ.836	753	814	836	chr1	119530260	**119530321**	+	0.972973
CH250_168c7TJ.825	192	276	825	chr1	119664263	**119664347**	+	0.964286
RP41_31a20TV.880	206	364	880	chr1	119690678	**119690520**	-	0.981132
RP41_0065D07.g1.705	396	509	705	chr1	119703847	119703734	-	1
CH250_168c7TV.892	52	565	892	chr1	119808674	**119808161**	-	0.973306
RP43_0078P13.b1.706	79	576	706	chr1	140709984	140709487	-	0.989362
RP43_121h2TV.678	318	404	678	chr1	141251782	**141251868**	+	0.988506
CH255_113g12TJ.606	130	302	606	chr1	141298487	141298315	-	0.959538
RP43_0150P09.g1.632	432	507	632	chr1	141349535	**141349460**	-	1
CH250_82l23TJ.305	68	305	305	chr1	142680877	142680640	-	0.941748
RP43_0086D24.g1.632	7	66	632	chr1	143036595	143036654	+	1
CH250_82l23TV.433	1	166	433	chr1	143692405	143692240	-	0.923611
CH250_157i3TV.834	607	690	834	chr1	144163994	144164077	+	0.987952
CH250_41a24TV.677	356	571	677	chr1	145503031	145502816	-	0.951087
RP43_0146F10.b1.698	421	498	698	chr1	146466476	146466399	-	1
CH255_112g4TJ.640	7	108	640	chr1	149047314	149047415	+	0.988636
CH255_196n5TJ.462	1	280	462	chr1	149177761	**149178040**	+	1
CH255_112g4TV.360	4	360	360	chr1	149261732	**149261376**	-	1
CH255_196n5TV.633	492	600	633	chr1	149393451	149393343	-	1
CH250_153a24TJ.772	639	769	772	chr1	150614117	150614247	+	0.962264
CH250_159f4TV.882	553	882	882	chr1	150656729	**150657058**	+	0.934066
CH250_159f4TJ.844	326	560	844	chr1	150817051	**150816817**	-	0.951327
RP41_0058M16.b1.757	285	516	757	chr1	152718327	**152718558**	+	0.971429
RP41_32a11TV.797	15	68	797	chr1	152852446	**152852393**	-	1
RP41_0058M16.g1.690	58	333	690	chr1	152903872	152903597	-	0.960396
CH250_31c14TV.754	115	222	754	chr1	154608239	154608346	+	0.962963
CH255_43a21TV.712	44	252	712	chr1	156110332	**156110540**	+	0.990244
CH255_43a21TJ.681	558	680	681	chr1	156281381	**156281259**	-	1
CH255_125l19TV.490	1	368	490	chr1	158044819	158045186	+	0.976027
CH255_218a4TJ.769	408	769	769	chr1	158053648	158054009	+	0.996047
CH255_235b12TJ.530	1	400	530	chr1	158063146	158063545	+	0.987952
RP41_0054J10.b1.732	82	279	732	chr1	158740635	**158740832**	+	0.969697
RP41_0054J10.g1.721	88	545	721	chr1	158884950	**158884493**	-	0.966759
CH250_160n11TV.873	486	551	873	chr1	168497127	168497192	+	0.969697
RP41_0068G06.b1.728	74	126	728	chr1	172027909	172027857	-	0.959184
CH250_162c6TV.837	34	201	837	chr1	176211987	176211820	-	0.973684
RP43_106d5TV.643	41	554	643	chr1	178140314	**178140827**	+	0.989733
RP43_106d5TJ.737	4	210	737	chr1	178304317	**178304111**	-	1
RP43_0039F01.b1.734	281	531	734	chr1	180662566	180662316	-	0.990476
RP41_0091F11.g1.736	322	443	736	chr1	183775618	**183775739**	+	0.957983
RP41_0091F11.b1.736	348	536	736	chr1	183926014	**183925826**	-	0.933884
CH250_86c12TV.878	335	500	878	chr1	185208177	**185208342**	+	0.932927
CH250_86c12TJ.845	431	577	845	chr1	185358908	**185358762**	-	0.986395
CH250_65m13TJ.860	295	768	860	chr1	185379480	185379007	-	0.970414

CH250_160n11TJ.862	608	741	862	chr1	192490900	192491033	+	0.954545
RP41_11j24TV.777	8	232	777	chr1	225622100	225622324	+	0.936275
CH250_1l23TJ.641	34	467	641	chr1	225674904	225675337	+	0.950131
CH255_143g16TV.845	7	227	845	chr1	239719020	239719240	+	0.994975
RP41_11i14TJ.840	11	108	840	chr1	243296279	243296376	+	0.956522
CH255_143g16TJ.775	1	149	775	chr1	245098587	245098735	+	0.992806
CH250_41a24TJ.847	386	785	847	chr1_rand	959469	959868	+	0.975741
CH250_153a24TV.672	15	162	672	chr1_rand	5015019	5014872	-	0.958678
RP41_42g16TV.893	622	681	893	chr10	577206	577147	-	0.945455
CH250_87e16TJ.755	454	512	755	chr10	2308397	2308455	+	0.953488
CH255_28j17TV.493	157	266	493	chr10	6633760	6633869	+	1
CH255_211g16TV.572	4	572	572	chr10	6716352	6716920	+	0.972028
CH255_211m15TV.369	31	361	369	chr10	6716394	6716724	+	0.951049
CH255_136b23TV.304	196	286	304	chr10	6728197	6728287	+	1
CH255_238b21TJ.166	1	166	166	chr10	6740446	6740611	+	1
CH255_118c16TV.810	4	810	810	chr10	6787185	6787991	+	0.983633
CH255_215c14TJ.642	5	414	642	chr10	6837335	**6837744**	+	0.994819
CH255_28j17TJ.324	97	324	324	chr10	6898388	**6898161**	-	0.976923
CH255_136b23TJ.772	650	772	772	chr10	6923669	6923547	-	1
CH255_211m15TJ.403	6	403	403	chr10	6924213	6923816	-	0.962025
CH255_211g16TJ.532	48	532	532	chr10	6924215	6923731	-	0.974522
CH255_31a20TJ.291	1	75	291	chr10	6936302	6936376	+	1
CH255_215c14TV.641	375	641	641	chr10	7028668	7028402	-	0.974359
CH255_226f3TV.696	6	182	696	chr10	7095996	7095820	-	0.99359
CH255_139m21TJ.831	2	461	831	chr10	11608744	11608285	-	0.985748
RP41_0087J05.b1.690	147	315	690	chr10	17629873	17630041	+	0.955128
RP43_0162P16.b1.684	94	644	684	chr10	30058338	**30058888**	+	0.985849
RP43_0162P16.g1.586	10	477	586	chr10	30193923	**30193456**	-	0.995
RP41_0094D08.g1.757	412	517	757	chr10	36775495	**36775600**	+	0.977273
RP41_0094D08.b1.755	37	499	755	chr10	36963527	**36963065**	-	0.928571
RP41_0079A06.b1.732	52	259	732	chr10	37066787	37066994	+	0.946078
CH250_163a22TJ.865	472	748	865	chr10	37584164	37584440	+	0.938182
RP43_132e19TV.565	513	565	565	chr10	43169530	43169478	-	1
RP41_0149C03.b1.742	330	407	742	chr10	45690747	45690670	-	0.95122
CH250_157l15TV.881	1	110	881	chr10	47779879	47779770	-	0.983333
CH255_214f21TV.562	5	562	562	chr10	49727645	49728202	+	0.982368
CH255_241i1TJ.825	1	105	825	chr10	49834828	**49834932**	+	1
CH255_230e13TJ.522	364	522	522	chr10	49952050	**49951892**	-	1
CH255_241i1TV.617	469	599	617	chr10	50087008	50086878	-	0.974576
CH255_18i8TV.360	41	360	360	chr10	50087726	50087407	-	0.995192
RP41_0155H22.b1.740	107	280	740	chr10	50257838	50258011	+	0.972222
CH250_157l15TJ.856	338	395	856	chr10	50833597	50833654	+	0.982759
RP41_0096B22.g1.753	219	527	753	chr10	55552799	55552491	-	0.947761
RP43_105d1TV.621	223	515	621	chr10	61199434	**61199726**	+	0.98913
RP43_105d1TJ.594	21	214	594	chr10	61374205	**61374012**	-	0.987879
CH250_1h5TJ.580	53	109	580	chr10	63318396	63318452	+	0.979592
RP41_0082H03.g1.753	380	531	753	chr10	63369779	**63369930**	+	0.964706
CH250_82f19TV.838	134	700	838	chr10	63402978	**63403544**	+	0.94484
CH250_61n13TV.711	216	407	711	chr10	63514427	**63514236**	-	0.953125
RP41_0082H03.b1.725	475	581	725	chr10	63523433	**63523327**	-	1
CH250_82f19TJ.795	713	762	795	chr10	63548296	63548247	-	1
RP43_0008F10.g1.628	155	308	628	chr10	69492390	**69492543**	+	0.992424

RP43_0008F10.b1.712	85	390	712	chr10	69708441	**69708136**	-	0.991968
CH250_145o15TV.865	801	853	865	chr10	75668660	75668712	+	1
RP41_0096B22.b1.770	386	445	770	chr10	81255128	81255069	-	1
RP43_0037D13.b1.756	79	693	756	chr10	85370292	**85370906**	+	0.978049
RP43_0037D13.g1.634	4	80	634	chr10	85559308	**85559232**	-	0.983333
RP43_0074F04.g1.632	445	505	632	chr10	85648368	85648308	-	1
RP41_18n20TJ.723	380	441	723	chr10	88780396	88780335	-	0.967742
CH255_110g1TJ.863	606	863	863	chr10	90599808	**90600065**	+	1
CH255_110g1TV.845	591	845	845	chr10	90788972	**90788718**	-	0.993631
CH255_142h23TV.787	379	740	787	chr10	90809123	90808762	-	0.992366
CH255_108l13TV.794	107	316	794	chr10	91329911	91330120	+	0.980952
CH255_10g22TJ.186	1	186	186	chr10	91384168	91384353	+	1
CH255_132m7TV.743	30	498	743	chr10	91423515	91423983	+	0.985037
CH255_4m10TJ.183	1	183	183	chr10	91483402	**91483584**	+	0.979167
CH255_108l13TJ.546	4	89	546	chr10	91560507	**91560422**	-	1
CH255_10g22TV.327	120	298	327	chr10	91591496	91591318	-	0.991597
CH255_132m7TJ.829	9	85	829	chr10	91610589	91610513	-	0.985507
CH255_4m10TV.170	13	168	170	chr10	91731393	91731238	-	0.982143
RP43_104n23TV.537	457	537	537	chr10	104615764	**104615844**	+	1
RP43_104n23TJ.348	210	269	348	chr10	104749589	**104749530**	-	1
RP41_0091N11.b1.775	127	206	775	chr10	105653429	**105653508**	+	0.95
RP41_0091N11.g1.734	308	502	734	chr10	105830173	**105829979**	-	0.921348
RP41_38o11TJ.817	533	771	817	chr10	116268071	**116268309**	+	0.990698
RP41_38o11TV.885	584	694	885	chr10	116438850	**116438740**	-	0.963303
CH255_25n20TJ.447	156	207	447	chr10	117195138	117195189	+	1
CH255_106i7TJ.635	3	511	635	chr10	117202555	117203063	+	0.991398
CH255_144b20TV.783	670	723	783	chr10	117203223	117203276	+	1
CH255_143o15TV.789	731	785	789	chr10	117203280	117203334	+	1
CH255_244o16TV.600	29	598	600	chr10	117309687	117310256	+	0.983209
CH255_241n11TJ.843	3	431	843	chr10	117319735	**117320163**	+	0.979328
CH255_25n20TV.497	44	497	497	chr10	117413595	**117413142**	-	0.970516
CH255_106i7TV.778	1	483	778	chr10	117413687	117413205	-	0.968182
CH255_143o15TJ.805	61	394	805	chr10	117464537	117464204	-	0.98722
CH255_144b20TJ.839	1	393	839	chr10	117464596	117464204	-	0.988235
CH255_244o16TJ.811	3	582	811	chr10	117467572	117466993	-	0.984762
CH255_107f11TV.835	87	267	835	chr10	117529879	117529699	-	1
CH255_133n10TJ.786	668	786	786	chr10	131455824	131455942	+	1
CH255_133n10TV.604	1	251	604	chr10	131880673	131880923	+	0.982906
CH255_30p2TJ.283	18	87	283	chr11	1583898	1583829	-	1
RP43_0010D20.b1.668	60	464	668	chr11	4095247	4095651	+	0.991124
RP43_0001O07.b1.709	396	538	709	chr11	4109376	**4109518**	+	1
RP43_107n8TJ.740	657	737	740	chr11	4200324	**4200244**	-	1
RP43_0001O07.g1.651	248	354	651	chr11	4328757	4328651	-	0.990291
RP43_0010D20.g1.661	255	361	661	chr11	4328757	4328651	-	0.980198
RP43_0042F24.b1.723	344	450	723	chr11	4328757	4328651	-	1
RP41_20e16TJ.861	4	238	861	chr11	21379086	21378852	-	0.990654
RP41_10j22TV.249	16	183	249	chr11	26126380	26126547	+	0.937984
RP41_0062J01.g1.689	110	420	689	chr11	26155713	26156023	+	0.938907
RP43_119e13TV.591	39	161	591	chr11	29663487	**29663609**	+	0.978947
RP43_119e13TJ.638	401	499	638	chr11	29833667	**29833569**	-	0.987805
RP41_0091E18.b1.702	58	281	702	chr11	33618216	33618439	+	0.962791
RP43_0090L07.b1.679	539	618	679	chr11	39963777	39963856	+	1

RP43_127f7TV.731	6	618	731	chr11	39993393	39994005	+	0.991319
RP43_0090L07.g1.626	385	581	626	chr11	40137802	40137606	-	0.97561
RP43_127f7TJ.851	19	244	851	chr11	40161257	40161032	-	0.981132
RP43_0092B22.b1.709	68	650	709	chr11	40189625	40189043	-	1
CH250_59a10TJ.592	1	307	592	chr11	41233350	41233656	+	0.959391
CH250_47d15TV.839	569	839	839	chr11	41287789	41288059	+	0.973684
CH250_39m18TJ.428	174	389	428	chr11	41331862	41332077	+	0.989796
CH250_167b9TJ.839	490	824	839	chr11	41345389	41345723	+	0.935018
CH250_47d15TJ.880	452	561	880	chr11	41440776	41440667	-	0.972727
CH250_39m18TV.688	276	356	688	chr11	41519091	41519011	-	0.950617
CH250_77e14TV.764	691	762	764	chr11	41520712	41520641	-	1
CH250_27j14TJ.790	413	464	790	chr11	41521006	41520955	-	0.980769
CH250_167b9TV.842	754	819	842	chr11	41524420	41524355	-	1
RP43_128n12TV.386	328	386	386	chr11	49107730	49107788	+	1
RP43_0031P04.g1.572	1	113	572	chr11	49108117	49108229	+	0.990476
RP43_0092J15.b1.713	44	644	713	chr11	49200624	49201224	+	0.993243
RP43_128n22TJ.886	1	709	886	chr11	49242155	49241447	-	0.992424
RP43_0092J15.g1.611	1	340	611	chr11	49245932	49245593	-	0.993976
RP43_0031P04.b1.733	468	573	733	chr11	49264873	49264768	-	1
CH255_229i9TJ.712	6	712	712	chr11	55034163	55034869	+	0.972713
CH255_24o3TV.364	5	364	364	chr11	55079936	55080295	+	0.972763
CH255_229i9TV.263	42	263	263	chr11	55193654	55193433	-	0.985714
RP43_0016G07.b1.703	60	337	703	chr11	55469659	55469936	+	0.980769
RP43_0159M23.g1.618	52	300	618	chr11	55689966	55689718	-	0.987854
RP41_43o15TJ.843	1	214	843	chr11	57070024	57070237	+	0.928571
CH255_124i12TJ.844	1	699	844	chr11	58034598	58035296	+	0.989614
CH255_134j11TV.708	28	103	708	chr11	58051344	58051419	+	1
CH255_116d19TV.618	5	618	618	chr11	58087850	58088463	+	0.985748
CH255_194m21TJ.770	1	133	770	chr11	58186624	58186756	+	0.990291
CH255_134j11TJ.798	22	126	798	chr11	58265667	58265563	-	0.973684
CH255_100m22TJ.522	5	56	522	chr11	58402405	58402354	-	1
CH255_194m21TV.481	393	481	481	chr11	58420556	58420468	-	0.9
CH255_198p6TJ.691	554	691	691	chr11	67556994	67556857	-	1
RP43_117p9TV.634	1	145	634	chr11	71330780	71330636	-	0.983607
RP43_116p21TV.763	3	154	763	chr11	71330787	71330636	-	0.984127
RP41_0063D10.g1.713	413	532	713	chr11	72527135	72527254	+	0.928571
RP41_0063D10.b1.782	315	594	782	chr11	72680990	72680711	-	0.983516
RP43_106d3TV.417	5	110	417	chr11	76893867	76893972	+	1
RP43_100k16TV.709	176	679	709	chr11	76954382	76954885	+	0.987539
RP43_106d3TJ.739	447	583	739	chr11	77048136	77048000	-	1
RP43_100k16TJ.729	19	89	729	chr11	77106276	77106206	-	1
RP43_132o6TV.709	139	709	709	chr11	77151393	77150823	-	0.995807
RP41_0153B21.b1.747	140	552	747	chr11	77810014	77810426	+	0.948339
RP41_0079A06.g1.783	370	437	783	chr11	80028857	80028924	+	0.96875
RP43_128n12TJ.275	5	275	275	chr11	89006125	89006395	+	0.987448
RP43_128n22TV.813	600	813	813	chr11	89134050	89133837	-	1
RP41_0063H12.g1.717	393	619	717	chr11	97099878	97100104	+	0.968504
RP41_0093N22.g1.761	78	424	761	chr11	107356144	107355798	-	0.953988
RP41_0064N05.b1.781	310	514	781	chr11	124982210	124982006	-	0.978022
CH255_230j16TV.545	162	231	545	chr11	126361041	126361110	+	1
RP41_0056P04.b1.773	471	570	773	chr11	129479319	129479418	+	1
RP41_0084L23.g1.750	140	499	750	chr11	129479319	129479678	+	0.978723

RP41_31c17TV.820	552	686	820	chr11	129601570	**129601704**	+	0.956897
RP41_0084L23.b1.745	61	257	745	chr11	129640772	**129640576**	-	0.930481
CH255_25n24TJ.168	1	105	168	chr12	5753402	5753298	-	1
RP43_117p9TJ.685	1	264	685	chr12	8259596	8259333	-	0.982222
RP43_0171M16.b1.714	48	311	714	chr12	8671341	8671078	-	0.991525
CH255_265i22TV.507	6	114	507	chr12	9536432	9536540	+	1
CH255_134e3TJ.726	411	625	726	chr12	13371269	13371483	+	1
CH255_223l24TV.829	2	598	829	chr12	13391292	13391888	+	0.98579
CH255_271k22TJ.633	2	92	633	chr12	13414475	**13414565**	+	0.985294
CH255_223l24TJ.859	128	399	859	chr12	13578365	**13578094**	-	0.988189
CH255_134e3TV.793	1	435	793	chr12	13617000	13616566	-	0.997579
CH255_271k22TV.604	327	450	604	chr12	13621177	13621054	-	0.99115
RP43_0092J08.b1.738	49	274	738	chr12	14743936	**14744161**	+	0.995283
RP43_0092J08.g1.640	5	315	640	chr12	14891692	**14891382**	-	0.996564
RP43_0084K05.g1.642	55	512	642	chr12	14931749	14931292	-	0.989189
RP43_0064A24.g1.631	140	287	631	chr12	20595772	20595625	-	1
RP41_0065L01.g1.714	100	329	714	chr12	23266353	**23266582**	+	0.960352
RP41_0065L01.b1.764	191	587	764	chr12	23444356	**23443960**	-	0.973154
RP41_0072L10.b1.722	46	115	722	chr12	29438345	29438276	-	1
CH255_265i22TJ.617	1	250	617	chr12	31018232	31018481	+	0.974138
CH255_218m11TV.362	117	362	362	chr12	31018823	31019068	+	0.981013
CH255_211d13TJ.425	6	185	425	chr12	31202306	31202485	+	1
CH255_TV.220	6	153	220	chr12	31202338	**31202485**	+	1
CH255_218m11TJ.738	53	151	738	chr12	31252824	**31252726**	-	0.989899
CH255_TJ.398	6	133	398	chr12	31394475	31394348	-	0.97479
RP43_0088A08.g1.634	235	518	634	chr12	31735776	31736059	+	1
RP43_0002J22.g1.634	41	272	634	chr12	31775357	**31775588**	+	0.986607
CH250_21m16TJ.537	379	472	537	chr12	31793783	**31793876**	+	0.945055
RP43_0088A08.b1.682	175	442	682	chr12	31889282	**31889015**	-	0.977477
CH250_92e23TV.880	56	283	880	chr12	31987590	**31987363**	-	0.961905
RP43_127h15TV.632	449	515	632	chr12	32008779	32008845	+	1
RP43_0011E17.g1.648	171	242	648	chr12	32056430	32056501	+	0.986111
RP43_127h15TJ.629	56	269	629	chr12	32161301	32161088	-	0.984211
RP43_0011E17.b1.726	39	634	726	chr12	32205862	32205267	-	0.995485
RP41_0060B06.b1.743	397	499	743	chr12	34202192	34202294	+	1
CH255_106m4TJ.535	250	535	535	chr12	34262656	34262941	+	0.972028
CH255_107e12TJ.562	206	562	562	chr12	34262656	34263012	+	0.977011
CH255_26k20TV.338	4	113	338	chr12	34291908	34292017	+	0.988372
CH255_266f19TV.434	3	104	434	chr12	34295869	**34295970**	+	1
CH255_114b1TV.672	235	672	672	chr12	34450800	**34450363**	-	0.985836
RP43_0161G01.b1.696	58	221	696	chr12	37692890	37693053	+	1
RP43_125h4TJ.852	17	781	852	chr12	37737657	**37738421**	+	0.988235
RP43_0161G01.g1.632	53	441	632	chr12	37885502	**37885114**	-	0.988539
RP43_125h4TV.871	6	202	871	chr12	37933261	37933065	-	1
RP43_0156N09.b1.720	76	464	720	chr12	40317063	40317451	+	0.986945
RP41_18h11TJ.796	2	261	796	chr12	51522427	**51522686**	+	0.944915
RP41_18h11TV.829	644	770	829	chr12	51689292	**51689166**	-	0.958763
CH250_151m15TJ.319	87	293	319	chr12	53504049	**53504255**	+	0.911765
CH250_151m15TV.569	163	244	569	chr12	53683992	**53683911**	-	1
RP43_136b17TJ.780	230	328	780	chr12	57816324	57816422	+	1
RP43_0168B11.b1.712	524	593	712	chr12	57989536	**57989605**	+	1
RP43_0168B11.g1.617	6	110	617	chr12	58145855	**58145751**	-	0.989362

CH255_28i21TV.407	34	407	407	chr12	64295322	64295695	+	0.996587
CH255_226i21TJ.775	720	775	775	chr12	65957107	65957162	+	1
CH255_140n7TV.799	495	758	799	chr12	66062205	**66062468**	+	0.995851
CH255_226i21TV.819	4	60	819	chr12	66153708	**66153652**	-	1
CH255_140n7TJ.695	109	427	695	chr12	66258679	66258361	-	0.984277
CH250_75n16TJ.546	448	533	546	chr12	69012728	**69012813**	+	0.986842
CH250_75n16TV.891	248	496	891	chr12	69193848	**69193600**	-	0.971888
CH250_68j17TV.790	4	110	790	chr12	69325337	69325231	-	0.978495
RP43_0078O15.b1.674	174	640	674	chr12	70727957	**70728423**	+	0.982699
RP43_0078O15.g1.637	486	597	637	chr12	70906185	**70906074**	-	1
RP43_0046M12.b1.715	62	386	715	chr12	75032735	**75033059**	+	0.996633
RP43_0046M12.g1.628	437	487	628	chr12	75223870	**75223820**	-	1
CH255_266i12TJ.541	30	515	541	chr12	78218096	78218581	+	0.975
CH255_240b24TJ.783	75	783	783	chr12	78223697	78224405	+	0.991453
CH255_128o22TJ.858	20	686	858	chr12	78247785	78248451	+	0.991961
CH255_2h7TJ.190	1	188	190	chr12	78315792	78315979	+	0.983871
CH255_46f4TV.508	232	505	508	chr12	78323387	78323660	+	0.978261
CH255_48f6TJ.391	48	391	391	chr12	78406514	**78406857**	+	0.994118
CH255_266i12TV.429	1	373	429	chr12	78422094	**78421722**	-	0.991031
CH255_240b24TV.614	4	614	614	chr12	78427807	78427197	-	0.988038
CH255_128o22TV.813	1	481	813	chr12	78453458	78452978	-	0.993392
CH255_46f4TJ.741	319	462	741	chr12	78549727	78549584	-	0.992908
CH255_48f6TV.544	193	248	544	chr12	78628930	78628875	-	0.979167
CH255_42n19TV.323	1	63	323	chr12	78685544	78685482	-	1
RP43_103n21TV.469	1	253	469	chr12	84266417	84266669	+	0.995708
RP43_0064I13.b1.653	73	424	653	chr12	84284765	**84285116**	+	0.993399
RP43_103n21TJ.292	45	292	292	chr12	84427923	**84427676**	-	0.990291
RP43_0064I13.g1.623	2	195	623	chr12	84476436	84476243	-	1
RP43_114d9TV.459	233	293	459	chr12	87030761	**87030821**	+	1
RP43_114d9TJ.730	7	277	730	chr12	87207506	**87207236**	-	0.984211
RP43_0092M12.b1.698	89	585	698	chr12	89180911	89181407	+	0.984043
CH250_68h14TV.626	269	355	626	chr12	94191068	**94191154**	+	0.965517
CH250_68h14TJ.863	1	127	863	chr12	94362372	**94362246**	-	0.979381
CH250_50j9TJ.520	229	515	520	chr12	94443727	94443441	-	0.973913
CH250_164j3TV.726	1	262	726	chr12	94491636	94491375	-	0.972603
CH255_127k22TJ.834	256	834	834	chr12	96505645	96506223	+	0.982792
CH255_230m2TJ.835	4	833	835	chr12	96576737	**96577566**	+	0.986919
CH255_230m2TV.844	216	270	844	chr12	96746067	**96746013**	-	1
CH255_127k22TV.628	268	601	628	chr12	96747572	96747239	-	0.995745
RP41_0072K13.g1.702	81	410	702	chr12	103427036	103427365	+	0.961905
CH255_106d12TV.855	422	855	855	chr12	103687689	**103688122**	+	0.984277
CH255_106d12TJ.751	4	276	751	chr12	103919781	**103919509**	-	0.992248
CH255_248e5TJ.806	636	694	806	chr12	126004028	126004086	+	0.945455
RP43_107p14TV.609	511	608	609	chr13	17077370	17077467	+	0.945946
RP43_0048A10.b1.744	403	521	744	chr13	17098478	**17098596**	+	0.985714
RP43_126l13TJ.762	138	762	762	chr13	17233754	**17233130**	-	0.992063
RP43_0152N19.g1.593	53	275	593	chr13	22103337	22103559	+	0.986425
RP43_0032H03.g1.606	212	375	606	chr13	22150087	22150250	+	1
RP43_107i4TJ.573	45	266	573	chr13	22181268	**22181489**	+	0.986301
RP43_0152N19.b1.686	75	130	686	chr13	22286237	**22286182**	-	0.981481
RP43_107i4TV.566	13	86	566	chr13	22349485	22349412	-	0.969697
RP43_0032H03.b1.701	388	467	701	chr13	22354137	22354058	-	1

RP41_24k19TV.846	694	789	846	chr13	23051303	23051208	-	0.983333
RP41_20l3TV.247	4	166	247	chr13	23051582	23051420	-	0.954545
RP41_20l19TV.806	116	212	806	chr13	23051826	23051730	-	1
RP43_126n22TJ.355	121	270	355	chr13	25831700	25831849	+	0.94
RP43_129j10TJ.326	138	287	326	chr13	25831700	25831849	+	0.916031
RP43_0147E05.g1.529	93	225	529	chr13	25831717	25831849	+	0.934426
RP43_101p2TV.160	6	125	160	chr13	25831732	25831851	+	0.93617
CH250_83i3TV.809	235	323	809	chr13	29470100	29470012	-	0.966292
CH250_147l22TJ.849	293	656	849	chr13	37175697	37176060	+	0.963788
CH250_3g18TJ.854	302	665	854	chr13	37175697	37176060	+	0.963889
CH255_7d21TJ.103	6	103	103	chr13	37223746	37223649	-	0.976744
CH250_160a2TV.741	124	195	741	chr13	37230733	37230804	+	0.957143
CH250_24e14TV.669	324	669	669	chr13	37253445	37253790	+	0.971429
CH250_24c18TV.738	331	734	738	chr13	37253445	37253848	+	0.971519
CH250_75h1TJ.838	371	816	838	chr13	37253445	37253890	+	0.957711
CH250_57m15TJ.634	510	570	634	chr13	37283230	**37283290**	+	1
CH250_147l22TV.897	602	668	897	chr13	37394975	**37394909**	-	1
CH250_160a2TJ.717	504	717	717	chr13	37417461	37417248	-	0.978022
CH255_217b23TV.563	4	265	563	chr13	39558571	39558310	-	0.987013
CH255_12n9TJ.227	18	134	227	chr13	46743807	46743923	+	1
CH255_115h12TV.685	13	304	685	chr13	46867839	46868130	+	0.996516
CH255_37k1TV.168	43	98	168	chr13	46964895	**46964950**	+	1
CH255_115h12TJ.609	526	609	609	chr13	47081425	**47081342**	-	1
CH255_144n9TJ.465	189	281	465	chr13	47098174	47098082	-	0.98
CH255_12k21TJ.228	1	67	228	chr13	47210757	47210691	-	1
CH255_37k1TJ.730	5	564	730	chr13	47214610	47214051	-	0.982906
CH250_159o1TJ.865	46	286	865	chr13	50371438	**50371678**	+	0.964758
RP43_0068H15.g1.643	387	497	643	chr13	50386979	50387089	+	1
CH250_159o1TV.871	40	503	871	chr13	50520801	**50520338**	-	0.965278
RP41_30b9TJ.800	615	686	800	chr13	58809080	58809151	+	0.972222
RP41_0052J10.g1.702	99	626	702	chr13	58897734	**58898261**	+	0.957295
RP41_0052J10.b1.797	220	273	797	chr13	59081555	**59081502**	-	0.962963
RP41_8g22TV.884	26	748	884	chr13	60897059	60897781	+	0.925
RP41_0079L23.g1.691	306	405	691	chr13	61515146	**61515245**	+	0.972222
RP41_0051O18.g1.726	502	555	726	chr13	61570391	**61570338**	-	1
RP41_0079L23.b1.725	274	550	725	chr13	61662469	61662193	-	0.937008
RP41_0075I08.g1.745	302	512	745	chr13	75735800	**75736010**	+	0.949153
RP41_0075I08.b1.734	175	280	734	chr13	75890954	**75890849**	-	0.933962
CH255_127i6TJ.829	1	815	829	chr13	78899285	**78900099**	+	0.988281
CH255_217b22TV.822	454	822	822	chr13	79162572	**79162204**	-	0.994595
CH255_234k20TV.550	320	550	550	chr13	79241459	79241229	-	1
RP41_8p20TJ.835	546	639	835	chr13	86427597	86427690	+	0.978261
RP41_0165D12.g1.722	268	322	722	chr13	87813254	**87813308**	+	0.963636
RP41_0165D12.b1.744	187	523	744	chr13	87961051	**87960715**	-	0.925926
RP41_8p20TV.864	744	837	864	chr13	90551635	90551728	+	0.926829
RP41_0095M03.g1.708	160	262	708	chr13	91126248	91126146	-	0.961165
CH250_172c10TJ.846	1	234	846	chr13	95686016	**95686249**	+	0.975369
RP41_0050D04.g1.751	257	530	751	chr13	95732167	95732440	+	0.945312
RP41_0068D04.b1.774	512	637	774	chr13	95754130	**95754255**	+	1
CH250_23a7TJ.466	312	466	466	chr13	95821229	**95821075**	-	1
CH250_172c10TV.888	712	786	888	chr13	95843921	95843847	-	0.971831
RP41_0050D04.b1.776	64	117	776	chr13	95888828	**95888775**	-	0.954545

RP41_0068D04.g1.688	103	203	688	chr13	95923724	95923624	-	0.976471
RP41_0072B21.b1.745	339	591	745	chr14	18937590	18937842	+	0.95302
RP43_0161K06.g1.620	3	432	620	chr14	18946695	**18947124**	+	0.977143
RP41_10g5TJ.750	396	750	750	chr14	19098959	**19099313**	+	0.948718
RP41_0072B21.g1.699	102	194	699	chr14	19110956	**19110864**	-	0.964706
RP43_0161K06.b1.717	67	578	717	chr14	19116461	**19115950**	-	0.985037
CH255_138p14TV.701	244	588	701	chr14	19141023	**19141367**	+	0.978873
CH250_23o20TV.689	426	688	689	chr14	19199998	19199736	-	0.937888
RP41_10g5TV.821	345	428	821	chr14	19229243	19229160	-	0.952381
CH255_138p14TJ.811	196	255	811	chr14	19318404	**19318345**	-	1
RP41_42o1TJ.824	305	389	824	chr14	22817437	22817521	+	0.951807
RP41_0083J15.g1.727	184	530	727	chr14	22836586	**22836932**	+	0.969466
RP41_0083J15.b1.739	113	197	739	chr14	23070298	**23070214**	-	0.976471
RP41_0159G06.b1.770	65	136	770	chr14	32757627	32757698	+	0.951613
RP41_0159C06.b1.805	537	641	805	chr14	32758104	32758208	+	1
RP41_0051N02.g1.725	340	548	725	chr14	33069025	33068817	-	0.977401
CH250_65b3TV.821	423	656	821	chr14	39846093	39846326	+	0.931818
CH250_80c22TV.596	197	465	596	chr14	39849955	**39850223**	+	0.943089
CH250_65b3TJ.848	559	650	848	chr14	40003285	**40003194**	-	0.973684
CH250_80c22TJ.573	252	573	573	chr14	40022331	40022010	-	0.950226
CH255_24d21TV.664	4	77	664	chr14	41756820	41756893	+	1
CH255_216c9TJ.534	188	311	534	chr14	41793623	41793746	+	1
CH255_28o3TJ.290	239	290	290	chr14	41813542	**41813593**	+	1
CH255_121a5TV.616	1	155	616	chr14	41872045	**41871891**	-	0.992593
CH255_216c9TV.453	7	267	453	chr14	41986027	41985767	-	0.97479
CH255_24d21TJ.357	2	181	357	chr14	42007378	42007199	-	0.99359
CH255_250e1TJ.839	730	797	839	chr14	42012961	42012894	-	1
CH255_28o3TV.442	39	149	442	chr14	42020553	42020443	-	0.990991
RP43_0008B11.g1.631	310	445	631	chr14	43532010	43532145	+	0.987952
CH250_24l7TJ.800	525	646	800	chr14	54786663	54786784	+	0.961538
RP43_0161M12.g1.628	133	495	628	chr14	56969202	56969564	+	1
RP43_0170G04.g1.587	323	550	587	chr14	57012160	**57012387**	+	0.985507
RP43_0161M12.b1.713	280	582	713	chr14	57126230	**57125928**	-	0.995283
RP43_0170G04.b1.681	409	564	681	chr14	57176116	57175961	-	0.972973
RP41_0085C08.g1.712	52	446	712	chr14	63871429	63871035	-	0.95053
RP41_28b8TJ.442	1	390	442	chr14	63933355	63932966	-	0.877193
RP41_0063L15.b1.764	145	232	764	chr14	85582164	85582077	-	0.943182
CH255_216g5TJ.725	498	725	725	chr14	86279907	**86280134**	+	1
CH255_7d13TJ.211	1	209	211	chr14	86384483	**86384275**	-	0.989691
CH255_262b17TJ.675	226	675	675	chr14	86478962	86478513	-	0.984326
RP41_0049I20.b1.785	44	322	785	chr14	89865058	89865336	+	0.976
RP41_35p19TJ.825	764	825	825	chr14	89877302	**89877363**	+	1
RP41_35p19TV.883	524	735	883	chr14	90075946	**90075735**	-	0.97619
RP41_0054H02.g1.716	333	433	716	chr14	90083718	90083618	-	0.980198
CH250_58l16TJ.855	396	713	855	chr15	35091917	**35092234**	+	0.968553
CH250_58l16TV.824	1	432	824	chr15	35254829	**35254398**	-	0.968825
CH255_112j3TJ.761	7	587	761	chr15	41154339	41153759	-	0.989228
RP43_0048A10.g1.662	456	548	662	chr15	44255403	44255311	-	1
RP41_0074C18.g1.690	167	230	690	chr15	45143272	45143335	+	0.949153
RP41_0074M02.g1.719	169	232	719	chr15	45143272	45143335	+	0.953125
RP41_0088G23.g1.752	318	404	752	chr15	45190335	**45190421**	+	0.980392
RP41_0088G23.b1.749	428	621	749	chr15	45223405	**45223212**	-	1

RP41_0067H14.g1.699	372	449	699	chr15	45362304	45362227	-	0.96
RP43_0068J13.b1.686	483	602	686	chr15	46316931	**46317050**	+	1
RP43_0068J13.g1.640	354	498	640	chr15	46495781	**46495637**	-	1
RP41_0095N07.g1.739	74	467	739	chr15	47616116	**47616509**	+	0.95203
RP41_0095N07.b1.757	144	361	757	chr15	47776055	**47775838**	-	0.943925
RP43_122i7TV.662	213	662	662	chr15	47816748	**47817197**	+	0.98893
RP43_122i7TJ.893	25	865	893	chr15	47926522	**47925682**	-	0.980597
CH250_43m21TV.734	557	610	734	chr15	53039577	53039630	+	0.967742
CH250_35e23TV.765	147	201	765	chr15	53087021	**53087075**	+	1
CH250_161h5TJ.854	626	730	854	chr15	53151400	**53151296**	-	0.961905
CH250_43m21TJ.507	105	459	507	chr15	53207709	53207355	-	0.950739
RP41_0050B19.g1.731	480	544	731	chr15	55282880	**55282944**	+	1
RP41_0050B19.b1.788	64	433	788	chr15	55447388	**55447019**	-	0.976959
RP41_0072A21.b1.748	278	361	748	chr15	55508508	55508425	-	0.940476
CH255_126h15TV.603	400	511	603	chr15	66253400	66253289	-	0.977778
RP41_0088L09.g1.753	51	274	753	chr15	69228033	69228256	+	0.948718
RP41_0068A16.b1.737	52	131	737	chr15	69268180	**69268259**	+	1
RP41_0088L09.b1.675	176	248	675	chr15	69419827	**69419755**	-	0.958904
RP41_0068A16.g1.723	358	533	723	chr15	69433720	69433545	-	0.96
RP41_0090L21.b1.773	191	323	773	chr15	95694506	**95694638**	+	0.932331
RP41_0090L21.g1.764	68	471	764	chr15	95842589	**95842186**	-	0.953488
RP43_0011J24.g1.630	142	265	630	chr16	10314690	10314813	+	1
RP43_0090O17.b1.682	152	212	682	chr16	10625559	10625499	-	1
RP43_0076H05.g1.626	88	460	626	chr16	10720715	10720343	-	0.993485
RP41_0059H10.b1.755	207	327	755	chr16	11775775	**11775895**	+	1
RP41_0059H10.g1.704	213	510	704	chr16	11929214	**11928917**	-	0.941176
RP41_43e12TV.807	152	262	807	chr16	16545494	16545604	+	0.972477
RP43_104e11TV.412	128	267	412	chr16	16855638	**16855777**	+	1
RP43_104e11TJ.730	631	730	730	chr16	17032576	**17032477**	-	1
RP41_0080K03.g1.734	298	511	734	chr16	18096599	18096812	+	0.973684
RP41_43e12TJ.808	89	182	808	chr16	18111601	**18111694**	+	0.978495
RP41_0080K03.b1.721	113	200	721	chr16	18263812	**18263725**	-	0.933333
RP41_0075J05.g1.740	60	336	740	chr16	22837597	22837873	+	0.942529
RP41_0065K08.b1.768	343	543	768	chr16	30447134	**30447334**	+	0.969231
RP41_0065K08.g1.737	232	380	737	chr16	30619960	**30619812**	-	0.964286
RP41_36k13TJ.745	66	149	745	chr16	30677905	30677822	-	0.943662
RP43_99b9TV.797	54	108	797	chr16	31559064	31559118	+	1
RP43_0094I07.g1.637	141	554	637	chr16	31583959	**31584372**	+	0.97037
RP43_99b9TJ.908	4	907	908	chr16	31649387	**31648484**	-	0.985222
RP43_0165G14.b1.720	78	146	720	chr16	32433021	32432953	-	0.982759
RP43_0148E12.g1.618	56	540	618	chr16	35625482	35624998	-	1
RP41_0085C03.g1.750	62	264	750	chr16	49484271	**49484473**	+	0.954286
RP41_0085C03.b1.701	394	487	701	chr16	49659441	**49659348**	-	1
CH255_102g1TJ.703	12	64	703	chr16	52949237	**52949289**	+	1
CH255_102g1TV.581	3	83	581	chr16	53179276	**53179196**	-	1
CH250_30k8TV.139	39	98	139	chr16	62433141	62433200	+	0.939394
CH250_42m9TJ.846	76	158	846	chr16	62442138	**62442220**	+	0.987179
CH250_30k8TJ.462	14	455	462	chr16	62631268	**62630827**	-	0.893701
RP41_0078D02.g1.716	74	497	716	chr16	63231247	**63231670**	+	0.94822
RP41_0078D02.b1.723	389	576	723	chr16	63416165	**63415978**	-	0.955882
RP43_0020J02.g1.644	34	259	644	chr16	77136752	77136977	+	0.981395
RP41_0051L12.g1.689	57	246	689	chr17	21276446	21276257	-	0.961111

CH255_112a3TJ.935	687	875	935	chr17	21496240	21496428	+	0.972477
CH255_30l3TV.489	424	489	489	chr17	21559176	21559241	+	1
CH255_140m7TJ.828	449	534	828	chr17	51172209	51172294	+	1
CH255_131m10TV.500	171	500	500	chr17	56704303	56704632	+	0.990066
CH255_131m10TJ.426	138	410	426	chr17	56881107	56880835	-	0.996241
RP41_0095G10.g1.730	318	418	730	chr17	59652035	59652135	+	0.952381
RP41_0095G10.b1.781	358	475	781	chr17	59852491	59852374	-	0.982456
RP41_46h15TJ.305	235	302	305	chr17	74939896	74939829	-	1
RP41_0051O18.b1.775	136	185	775	chr17_ran	1737734	1737783	+	0.979592
CH255_202d17TJ.652	1	57	652	chr18	1872957	1873013	+	1
CH255_12e19TV.281	179	281	281	chr18	1938393	1938495	+	1
CH255_11k17TJ.422	3	422	422	chr18	4181134	4181553	+	0.991968
RP43_126l13TV.710	4	431	710	chr18	14337889	14337462	-	0.994975
RP43_121h2TJ.771	605	771	771	chr18	14373954	14373788	-	1
RP41_10m12TV.869	7	214	869	chr18	28665361	28665568	+	0.939759
RP43_0151H01.g1.599	7	388	599	chr18	30461128	30461509	+	0.985915
RP43_0151H01.b1.706	263	312	706	chr18	30608641	30608592	-	0.98
RP43_0015D12.g1.628	388	514	628	chr18	31557744	31557870	+	1
RP43_0146E13.g1.622	287	429	622	chr18	31639168	31639310	+	0.984252
RP43_0042H03.b1.747	260	386	747	chr18	31648648	31648774	+	0.976378
CH250_69o23TV.713	600	668	713	chr18	32745365	32745433	+	1
CH250_69o23TJ.884	23	105	884	chr18	32953250	32953168	-	0.983607
CH255_234g7TJ.677	234	677	677	chr18	47159685	47160128	+	0.997268
CH255_34k4TV.719	350	719	719	chr18	47285867	47285498	-	0.971264
CH255_234g7TV.503	212	491	503	chr18	47370071	47369792	-	0.983784
CH255_12i11TV.399	5	399	399	chr18	49704814	49705208	+	0.989529
RP41_0061O18.b1.788	384	559	788	chr18	49789665	49789840	+	0.955357
CH255_12i11TJ.302	32	302	302	chr18	49884393	49884123	-	0.986842
RP41_18k21TJ.852	799	852	852	chr18	49965079	49965026	-	1
RP41_0147G19.g1.745	73	292	745	chr18	52283997	52284216	+	0.971014
RP41_0073O15.g1.706	316	455	706	chr18	52327085	52327224	+	0.931507
RP41_0147G19.b1.771	69	205	771	chr18	52455940	52455804	-	0.916667
RP41_0073O15.b1.753	62	173	753	chr18	52496937	52496826	-	0.962963
RP43_114j3TJ.567	4	231	567	chr18	66059896	66060123	+	0.986425
RP43_114j3TV.282	69	282	282	chr18	66247280	66247067	-	0.994975
RP43_0164H03.b1.706	458	572	706	chr18	67118836	67118950	+	1
RP43_0164H03.g1.608	41	211	608	chr18	67278404	67278234	-	0.992593
CH255_254m13TV.576	322	576	576	chr19	19746410	19746664	+	0.987805
CH255_268o1TV.490	289	365	490	chr19	19937632	19937556	-	1
CH255_254m13TJ.745	1	724	745	chr19	19977943	19977220	-	0.982759
CH255_268o1TJ.609	3	77	609	chr19	20178718	20178644	-	0.972222
CH255_230g7TJ.636	6	636	636	chr19	20180405	20179775	-	0.982111
CH250_24k15TV.773	707	759	773	chr19	20781248	20781300	+	1
CH255_128a9TJ.527	5	61	527	chr19	20924092	20924148	+	1
RP41_42d22TV.885	629	718	885	chr19	21488852	21488941	+	0.955556
RP41_42d22TJ.606	1	371	606	chr19	21612649	21613019	+	0.955357
CH255_130p24TJ.383	75	381	383	chr19	21781410	21781104	-	0.986348
CH255_140m10TV.822	302	379	822	chr19	21842290	21842213	-	0.987179
CH255_234k18TJ.627	524	615	627	chr19	22238876	22238967	+	1
CH255_4i8TV.295	4	295	295	chr19	22832805	22833096	+	1
CH255_32m16TV.439	1	439	439	chr19	23120663	23120225	-	0.991892
CH250_27m2TJ.343	1	133	343	chr19	23206894	23206762	-	0.970874

RP41_0056J07.g1.737	108	168	737	chr19	32796639	32796699	+	0.95082
RP43_98j20TV.877	793	877	877	chr19	32898238	32898322	+	0.979592
RP43_0070G08.b1.657	111	367	657	chr19	32962045	**32962301**	+	0.987013
RP43_98j20TJ.730	3	356	730	chr19	33035374	**33035021**	-	0.987692
RP43_0068B15.g1.616	46	512	616	chr19	33095658	33095192	-	0.991501
RP43_0167O05.g1.610	12	487	610	chr19	33095694	33095219	-	0.990074
RP43_0070G08.g1.645	4	160	645	chr19	33135712	33135556	-	0.990909
CH250_53p2TV.755	129	364	755	chr19	38194135	**38194370**	+	0.965957
CH250_53p2TJ.844	710	832	844	chr19	38360707	**38360585**	-	0.964286
RP43_0076L03.b1.634	102	333	634	chr19	42572297	42572528	+	1
RP43_126o24TV.290	1	113	290	chr19	42572533	**42572645**	+	1
RP43_126o24TJ.743	330	743	743	chr19	42747304	**42746891**	-	0.992248
RP43_102k22TJ.772	64	772	772	chr19	42795509	42794801	-	1
RP43_123l17TJ.650	588	650	650	chr19	47964525	47964463	-	1
RP43_123l17TV.762	649	762	762	chr19	48203850	48203963	+	0.983607
RP43_0040N21.g1.616	42	449	616	chr19	48602357	**48602764**	+	0.975684
RP43_0004B20.b1.682	119	453	682	chr19	48728922	**48728588**	-	0.987069
RP43_0040N21.b1.701	42	544	701	chr19	48782761	48782259	-	0.995169
CH250_148j3TV.691	75	310	691	chr2	4209530	**4209765**	+	0.946429
RP41_0161O24.g1.688	52	130	688	chr2	4247027	**4247105**	+	0.945946
CH250_148j3TJ.728	11	243	728	chr2	4367524	**4367292**	-	0.982456
RP41_0053H13.g1.693	58	550	693	chr2	4378320	**4377828**	-	0.954407
CH255_206b7TV.557	47	557	557	chr2	18355218	**18355728**	+	0.974886
CH255_206b7TJ.753	136	188	753	chr2	18532974	**18532922**	-	1
RP43_129l3TV.602	1	99	602	chr2	20673540	**20673638**	+	1
RP43_129l3TJ.615	14	613	615	chr2	20821915	**20821316**	-	0.985891
RP43_0153D12.b1.702	63	352	702	chr2	28708309	**28708598**	+	0.992754
RP43_0153D12.g1.616	302	509	616	chr2	28900464	**28900257**	-	0.984615
CH250_23m17TJ.506	245	328	506	chr2	30762936	30763019	+	0.910714
CH250_5a4TJ.359	255	338	359	chr2	30762936	30763019	+	0.927536
CH250_166h20TJ.650	1	123	650	chr2	30804793	30804915	+	0.9125
CH250_33m22TV.405	286	368	405	chr2	30840988	**30841070**	+	1
CH250_33m22TJ.847	434	631	847	chr2	30992601	**30992404**	-	0.93299
CH250_166h20TV.792	218	291	792	chr2	31001600	31001527	-	0.958904
RP43_0043D10.b1.726	377	427	726	chr2	31490824	31490874	+	1
RP43_0157F05.b1.701	188	626	701	chr2	33796948	33797386	+	0.983553
RP41_0078M08.b1.665	378	428	665	chr2	47653364	47653314	-	1
RP43_0034M03.b1.684	69	351	684	chr2	53259821	**53260103**	+	0.985663
RP43_0034M03.g1.622	2	386	622	chr2	53387427	**53387043**	-	0.997167
RP41_6d15TV.848	332	460	848	chr2	55233256	**55233384**	+	0.953488
RP41_0085G22.g1.734	54	175	734	chr2	55435713	**55435592**	-	0.9
CH250_93j17TV.895	365	529	895	chr2	62483372	62483536	+	0.939394
CH250_35m9TJ.555	164	263	555	chr2	62517355	62517454	+	0.895833
CH250_57n22TV.694	465	575	694	chr2	62529197	**62529307**	+	0.963636
CH250_37n10TJ.334	188	323	334	chr2	62626297	**62626162**	-	0.980392
CH250_93j17TJ.814	201	329	814	chr2	62626297	62626169	-	0.976744
CH250_35m9TV.778	692	778	778	chr2	62662944	62662858	-	1
CH250_145b11TJ.828	1	61	828	chr2	62694440	62694380	-	1
CH255_258n23TV.815	766	815	815	chr2	63205844	63205893	+	1
CH255_138m5TJ.722	613	722	722	chr2	63245934	63246043	+	1
CH255_128i9TV.820	636	724	820	chr2	63363182	**63363270**	+	1
CH255_258n23TJ.823	605	727	823	chr2	63408900	**63408778**	-	0.991736

CH255_138m5TV.592	1	133	592	chr2	63492552	63492420	-	0.990099
CH255_128i9TJ.822	83	253	822	chr2	63586154	63585984	-	0.987578
RP43_0025E22.g1.623	389	464	623	chr2	65443300	**65443375**	+	1
RP43_0170D21.g1.617	52	477	617	chr2	65559212	**65558787**	-	0.985755
RP43_0025E22.b1.712	166	640	712	chr2	65576087	65575613	-	0.996979
CH255_26c21TV.438	7	99	438	chr2	67099826	67099918	+	1
CH255_204c16TJ.740	518	740	740	chr2	67114091	67114313	+	0.990476
CH255_233l21TJ.781	34	222	781	chr2	67218835	**67219023**	+	0.988024
CH255_200b16TV.718	649	718	718	chr2	67307201	**67307132**	-	1
CH255_233l21TV.208	6	203	208	chr2	67330161	67329964	-	0.967742
CH255_204c16TV.799	1	93	799	chr2	67337566	67337474	-	1
RP43_0094A01.g1.629	267	459	629	chr2	70494809	70495001	+	1
RP43_101i7TV.581	301	413	581	chr2	70550884	70550996	+	1
RP43_0034K03.b1.713	60	363	713	chr2	70617671	**70617974**	+	0.989474
RP43_0094A01.b1.707	486	590	707	chr2	70676489	**70676385**	-	1
RP43_101i7TJ.717	328	492	717	chr2	70685496	70685332	-	1
RP43_0034K03.g1.626	43	469	626	chr2	70798367	70797941	-	0.993976
RP41_0093J11.b1.756	239	475	756	chr2	70834595	**70834831**	+	0.95
RP41_0093J11.g1.731	43	310	731	chr2	70883947	**70883680**	-	0.960474
CH255_46f24TV.556	342	556	556	chr2	71176157	71176371	+	0.967213
CH255_46f24TJ.678	4	678	678	chr2	71404767	71404093	-	0.994129
RP41_0161L17.b1.734	176	528	734	chr2	78022075	78022427	+	0.951768
RP41_0079A12.g1.759	67	311	759	chr2	79220254	**79220498**	+	0.952174
RP41_44n2TJ.826	589	782	826	chr2	79442437	**79442244**	-	0.954802
RP41_0054P08.b1.771	61	377	771	chr2	79506585	79506269	-	0.974603
RP43_0002C21.g1.651	37	501	651	chr2	83418088	**83418552**	+	0.985423
RP43_0002C21.b1.708	72	595	708	chr2	83580715	**83580192**	-	0.989583
RP43_107c1TV.686	634	686	686	chr2	87463429	87463481	+	1
RP41_35f19TV.862	295	383	862	chr2	95118841	95118929	+	0.966292
RP41_37d4TV.903	552	612	903	chr2	100814565	100814625	+	1
RP43_107c1TJ.576	43	576	576	chr2	112282928	112283461	+	0.991489
CH250_150n19TJ.809	258	496	809	chr2	116963756	**116963994**	+	0.896104
CH250_150n19TV.603	1	101	603	chr2	117148102	**117148002**	-	0.984375
CH255_124g9TV.538	2	66	538	chr2	129358954	129359018	+	1
CH255_265d6TV.615	1	519	615	chr2	129399256	129399774	+	0.964953
CH255_250l4TJ.265	3	265	265	chr2	129458416	129458678	+	0.990826
CH255_126a6TJ.845	32	147	845	chr2	129473881	**129473996**	+	0.978495
CH255_265d6TJ.624	423	624	624	chr2	129590362	**129590161**	-	1
CH255_250l4TV.390	3	388	390	chr2	129673610	129673225	-	0.980769
CH255_126a6TV.785	481	696	785	chr2	129689866	129689651	-	0.985612
RP43_98a16TJ.210	55	172	210	chr2	133247673	133247790	+	1
CH250_77g4TV.737	444	505	737	chr2	140972595	140972656	+	1
CH250_165n13TJ.830	111	160	830	chr2	140975400	**140975449**	+	1
CH250_165n13TV.891	516	604	891	chr2	141111378	**141111290**	-	0.988764
CH250_162f21TJ.846	563	677	846	chr2	142146083	142146197	+	0.964912
CH250_147d12TV.877	797	877	877	chr2	142167568	142167648	+	0.911765
CH250_9j16TJ.742	545	634	742	chr2	142195969	142196058	+	0.984375
CH250_12d8TJ.810	545	682	810	chr2	142195969	**142196106**	+	0.983871
RP43_0151J13.b1.721	32	317	721	chr2	142199448	**142199733**	+	0.981413
CH250_86e17TJ.850	452	556	850	chr2	142337324	**142337220**	-	1
CH250_63j16TJ.736	51	143	736	chr2	142348857	142348765	-	0.964706
CH250_9j16TV.534	3	95	534	chr2	142348857	142348765	-	0.953846

RP43_0070B15.g1.633	13	521	633	chr2	142355976	**142355468**	-	0.975962
CH250_172e2TJ.844	142	275	844	chr2	143601590	143601723	+	0.984496
CH250_92j13TJ.838	95	160	838	chr2	143662193	**143662258**	+	0.983871
RP43_114h16TJ.777	55	777	777	chr2	143684971	143685693	+	0.996593
CH250_172e2TV.874	113	190	874	chr2	143762549	**143762472**	-	1
CH250_92j13TV.809	431	513	809	chr2	143811041	143810959	-	0.948052
RP43_114h16TV.760	1	61	760	chr2	143880427	143880367	-	0.975
RP43_0028D10.g1.623	4	460	623	chr2	155766265	155766721	+	0.98977
RP43_104f13TV.892	13	427	892	chr2	155831042	155831456	+	0.994505
RP43_126o23TV.877	1	414	877	chr2	155831043	155831456	+	0.991935
RP43_104h21TV.533	5	333	533	chr2	155831128	**155831456**	+	0.996689
RP43_104h21TJ.834	54	222	834	chr2	155953467	**155953299**	-	0.993421
RP43_104f13TJ.910	5	223	910	chr2	155953517	155953299	-	0.994624
CH250_58d2TV.778	117	341	778	chr2	157252907	157253131	+	0.941964
CH255_34l22TV.785	6	708	785	chr2	158998386	158997684	-	0.987013
RP41_43e14TV.872	590	746	872	chr2	162135396	**162135552**	+	0.986928
RP41_43e14TJ.636	496	612	636	chr2	162300375	**162300259**	-	0.974359
CH255_113g20TJ.552	6	552	552	chr2	163557697	**163558243**	+	0.990741
CH255_113g20TV.764	257	764	764	chr2	163770904	**163770397**	-	0.986413
RP41_0092A05.g1.693	266	461	693	chr2	167445212	167445017	-	0.9125
RP43_0086H24.g1.568	4	297	568	chr2	182090556	**182090849**	+	0.991071
RP43_0086H24.b1.660	73	576	660	chr2	182268421	**182267918**	-	0.992063
CH255_230i15TJ.476	128	190	476	chr2	188680547	188680609	+	1
CH255_241k9TV.536	55	252	536	chr2	188691649	188691846	+	0.994318
CH255_37o13TJ.477	96	200	477	chr2	188692607	188692711	+	1
CH255_38j19TJ.921	664	921	921	chr2	188756059	**188756316**	+	1
CH255_230i15TV.261	2	235	261	chr2	188894614	**188894381**	-	0.992481
CH255_37o13TV.538	4	146	538	chr2	188932530	188932388	-	1
CH255_38j19TV.357	4	63	357	chr2	188942305	188942246	-	0.980769
CH255_31p19TJ.119	55	119	119	chr2	194378436	194378500	+	1
CH255_240d2TV.754	376	454	754	chr2	194495309	194495387	+	1
CH255_242a20TV.764	5	160	764	chr2	200338533	200338378	-	1
RP43_0052B04.g1.666	5	580	666	chr2	209388653	209388078	-	0.994536
RP43_0017G20.b1.731	78	589	731	chr2	209388663	209388152	-	0.995098
RP43_107p6TJ.883	68	883	883	chr2	211271103	**211271918**	+	0.995816
RP43_107p6TV.667	46	667	667	chr2	211453092	**211452471**	-	0.985663
CH255_264l9TJ.261	2	261	261	chr2	214486152	214486411	+	0.987013
CH255_235j12TV.582	393	581	582	chr2	214495836	214496024	+	0.979798
CH255_130a11TJ.800	520	588	800	chr2	214504465	**214504533**	+	1
CH250_89m17TJ.850	455	748	850	chr2	214618761	214618468	-	0.963504
CH255_264l9TV.844	725	819	844	chr2	214677141	**214677047**	-	1
CH255_130a11TV.577	2	334	577	chr2	214760434	214760102	-	0.993289
RP43_132e3TJ.797	452	797	797	chr2	215774885	215775230	+	1
RP43_0019M14.g1.634	6	212	634	chr2	215826187	**215826393**	+	0.98995
RP43_132e3TV.789	375	789	789	chr2	215954325	**215953911**	-	0.995169
RP43_0019M14.b1.705	77	522	705	chr2	216015813	216015368	-	0.993266
CH255_197a16TJ.612	1	612	612	chr2	217187048	217187659	+	0.987654
RP41_0058L04.b1.732	31	136	732	chr2	232323423	232323318	-	0.978261
RP41_0167B03.b1.740	301	424	740	chr2	234433376	**234433499**	+	0.960526
RP41_0052O01.g1.698	417	488	698	chr2	234547177	**234547106**	-	1
RP41_0081F24.b1.766	418	494	766	chr2	234615221	234615145	-	0.971429
RP41_0167B03.g1.705	377	483	705	chr2	234615523	234615417	-	1

RP41_0155I17.b1.795	263	354	795	chr2	239262590	239262499	-	0.967391
RP43_0005M23.g1.634	43	359	634	chr20	5825841	**5826157**	+	0.990536
RP43_0005M23.b1.691	36	287	691	chr20	6012147	**6011896**	-	0.986784
CH250_7a3TJ.657	1	69	657	chr20	8343321	8343389	+	0.981818
CH250_164i18TV.888	631	771	888	chr20	8405727	**8405867**	+	0.976562
CH250_7a3TV.376	47	325	376	chr20	8522426	**8522148**	-	0.917603
CH250_164i18TJ.867	6	675	867	chr20	8584501	8583832	-	0.96124
CH255_130p24TV.417	101	157	417	chr20	30759046	30758990	-	1
CH255_270g5TV.283	1	216	283	chr20	33257385	33257600	+	0.988701
RP43_127k10TJ.423	1	423	423	chr20	33307731	33308153	+	0.996785
CH255_140a21TJ.827	373	430	827	chr20	33340504	**33340561**	+	1
RP43_0092G08.g1.613	3	485	613	chr20	33341457	33341939	+	1
CH255_270g5TJ.245	159	245	245	chr20	33481423	**33481337**	-	1
CH255_140a21TV.799	232	799	799	chr20	33493876	33493309	-	0.991131
RP43_0019B06.b1.650	100	351	650	chr20	35194944	**35195195**	+	0.985507
RP43_0019B06.g1.642	1	95	642	chr20	35349366	**35349272**	-	1
RP41_0072B10.g1.708	95	569	708	chr20	44129922	**44130396**	+	0.949405
RP41_0072B10.b1.715	72	160	715	chr20	44331571	**44331483**	-	0.988372
CH250_175o11TV.874	38	825	874	chr21	10043346	10042559	-	0.957447
CH250_53a14TJ.859	681	788	859	chr21	13694562	13694455	-	0.97
RP43_104l13TJ.503	286	490	503	chr21	14163082	14162878	-	0.970297
RP43_107f14TV.653	549	652	653	chr21	14266889	14266786	-	1
RP43_119e10TJ.443	50	142	443	chr21	14294315	14294223	-	0.916667
CH255_114e13TV.678	541	612	678	chr21	14398067	14398138	+	0.982143
CH255_119e17TV.776	568	629	776	chr21	14728869	14728930	+	0.981818
CH255_223j11TJ.646	560	646	646	chr21	14753089	14753175	+	1
CH255_126l9TJ.838	564	815	838	chr21	14796449	**14796700**	+	1
CH255_119e17TJ.716	4	714	716	chr21	14912801	**14912091**	-	0.987868
CH255_236o1TJ.822	771	822	822	chr21	14936834	14936783	-	1
CH255_223j11TV.816	5	816	816	chr21	14950051	14949240	-	0.987382
CH255_126l9TV.784	5	403	784	chr21	15022168	15021770	-	0.98939
CH250_162b3TJ.777	172	643	777	chr21	15639458	**15639929**	+	0.942308
CH250_162b3TV.855	237	855	855	chr21	15802932	**15802314**	-	0.936535
CH255_102d21TJ.825	4	57	825	chr21	23393882	23393935	+	1
CH255_102d21TV.901	1	901	901	chr21	23620677	23619777	-	0.983226
RP43_0084L24.b1.698	139	206	698	chr21	26150015	26149948	-	1
RP41_0072P21.b1.761	69	368	761	chr21	26500879	**26501178**	+	0.951049
RP41_0072P21.g1.726	392	487	726	chr21	26717222	**26717127**	-	0.951613
CH255_256f22TV.836	7	449	836	chr21	27389208	**27389650**	+	0.985258
CH255_235h7TJ.306	228	295	306	chr21	27528589	**27528522**	-	0.967742
CH255_4b20TJ.206	7	114	206	chr21	27582842	27582735	-	0.984848
CH255_256f22TJ.841	588	841	841	chr21	27584866	27584613	-	0.990783
CH255_266f13TJ.771	1	761	771	chr21	27586673	27585913	-	0.989164
CH255_100g8TV.225	5	102	225	chr21	35445505	35445602	+	1
CH255_223m20TV.624	190	624	624	chr21	35622261	35622695	+	0.989362
CH255_223m20TJ.628	460	628	628	chr21	35848198	35848030	-	1
RP43_116e9TV.741	642	711	741	chr21	36180734	36180665	-	1
CH250_24i18TJ.751	45	192	751	chr22	14684818	14684671	-	0.954198
RP43_0173I23.b1.696	424	633	696	chr22	15714746	15714537	-	0.984848
RP41_12e9TJ.732	2	106	732	chr22	15768694	15768590	-	0.946667
RP41_0051M03.b1.769	76	513	769	chr22	29055078	29055515	+	0.970027
RP41_0167D14.b1.759	70	560	759	chr22	29118350	29118840	+	0.969492

RP41_0149P02.b1.763	43	273	763	chr22	29220775	**29221005**	+	0.926108
RP41_0167D14.g1.738	307	484	738	chr22	29312015	**29311838**	-	0.967391
RP41_20g16TV.845	4	82	845	chr3	6494798	6494720	-	1
CH250_27g9TJ.502	355	409	502	chr3	9543268	9543322	+	1
RP41_22f21TJ.484	3	131	484	chr3	12891296	12891168	-	0.962963
RP43_0054I17.b1.727	81	585	727	chr3	13413391	**13413895**	+	0.992386
RP43_0054I17.g1.626	316	569	626	chr3	13581979	**13581726**	-	1
RP43_127c4TV.766	464	766	766	chr3	19460731	**19461033**	+	0.994253
RP43_127c4TJ.862	3	862	862	chr3	19603992	**19603133**	-	0.991525
RP43_0080I06.g1.653	4	574	653	chr3	19659345	19658775	-	0.982843
RP43_133h13TV.245	1	242	245	chr3	39284395	**39284636**	+	1
RP43_133h13TJ.746	647	700	746	chr3	39452344	**39452291**	-	1
RP41_0068C09.g1.690	57	146	690	chr3	51535003	51535092	+	1
RP41_0075M07.b1.686	52	186	686	chr3	51858504	51858370	-	0.980583
CH255_222k23TJ.812	48	175	812	chr3	58769898	**58770025**	+	1
CH255_222k23TV.852	3	392	852	chr3	58971338	**58970949**	-	0.994269
RP41_0078M07.g1.749	321	485	749	chr3	66464319	**66464483**	+	0.979381
RP41_0078M07.b1.659	1	384	659	chr3	66644875	**66644492**	-	0.928205
CH255_246p15TJ.849	168	846	849	chr3	74029427	74030105	+	0.98
CH255_143l23TJ.853	62	853	853	chr3	74041330	74042121	+	0.986684
CH255_206k3TV.676	588	676	676	chr3	74059181	74059269	+	1
CH255_214f22TJ.534	168	534	534	chr3	74068726	**74069092**	+	0.985019
CH255_246p15TV.640	292	640	640	chr3	74214996	**74214648**	-	0.986928
CH255_143l23TV.809	1	808	809	chr3	74230704	74229897	-	0.987887
CH255_206k3TJ.749	20	749	749	chr3	74237089	74236360	-	0.98928
CH255_214f22TV.417	353	417	417	chr3	74302426	74302362	-	1
CH255_112e2TV.570	2	570	570	chr3	75171690	75172258	+	0.983516
RP43_103d7TV.593	291	391	593	chr3	75362065	75361965	-	0.989899
CH255_198p6TV.476	160	233	476	chr3	75400670	75400743	+	1
RP43_0019L04.b1.722	28	193	722	chr3	87680192	87680357	+	0.978571
RP43_0164D05.b1.729	77	243	729	chr3	87694765	**87694931**	+	0.981818
RP43_0019L04.g1.660	259	478	660	chr3	87858753	**87858534**	-	0.987654
RP43_0164D05.g1.628	5	268	628	chr3	87865193	87864930	-	0.988281
RP43_109d22TV.648	6	648	648	chr3	88065318	88065960	+	0.991753
RP43_0014E15.b1.710	59	519	710	chr3	88115666	**88116126**	+	0.992683
RP43_0157E06.g1.615	52	526	615	chr3	88227512	**88227038**	-	0.98645
RP43_0014E15.g1.635	451	511	635	chr3	88276756	88276696	-	1
RP43_0012H11.g1.647	1	493	647	chr3	95338659	**95339151**	+	0.995157
RP43_0012H11.b1.700	373	545	700	chr3	95522895	**95522723**	-	0.983471
CH255_268j11TV.815	637	741	815	chr3	96167351	96167455	+	0.984615
CH250_158j15TJ.860	123	297	860	chr3	104195145	104195319	+	0.940828
RP41_6f20TJ.786	512	785	786	chr3	104287292	104287019	-	0.955975
CH255_37a8TV.614	22	200	614	chr3	107868411	107868589	+	0.987879
CH255_134l15TV.152	1	152	152	chr3	107891268	107891419	+	1
CH255_265c11TJ.569	1	197	569	chr3	107934782	107934978	+	0.989848
CH255_223i5TJ.739	450	513	739	chr3	108004055	108004118	+	1
CH255_16m13TJ.276	217	276	276	chr3	108027333	108027392	+	1
CH255_222m6TJ.696	543	694	696	chr3	108072497	**108072648**	+	0.981481
CH255_37a8TJ.648	3	648	648	chr3	108092019	**108091374**	-	0.988571
CH255_115m16TJ.786	111	210	786	chr3	108124764	108124665	-	0.99
CH255_134l15TJ.770	1	58	770	chr3	108149396	108149339	-	1
CH255_229n7TV.804	332	804	804	chr3	108166656	108166184	-	0.982609

CH255_265c11TV.555	149	555	555	chr3	108169557	108169151	-	0.985119
CH255_223i5TV.860	1	692	860	chr3	108223257	108222566	-	0.986641
CH255_16m13TV.277	8	276	277	chr3	108270717	108270449	-	0.979021
CH255_134p19TV.513	1	493	513	chr3	108276099	108275607	-	0.984556
CH255_222m6TV.694	1	694	694	chr3	108276101	108275408	-	0.984155
CH255_259h24TV.753	25	138	753	chr3	121474571	121474684	+	1
CH255_40e7TV.268	100	268	268	chr3	121552299	**121552467**	+	0.992806
CH255_222b1TV.715	261	347	715	chr3	121672801	**121672715**	-	0.988506
CH255_259h24TJ.837	725	837	837	chr3	121682881	121682769	-	1
CH255_253a6TJ.364	1	173	364	chr3	121734626	121734454	-	1
CH255_40e7TJ.448	44	178	448	chr3	121767196	121767062	-	0.984
RP43_0030P05.b1.712	74	543	712	chr3	123377660	**123378129**	+	0.992
RP43_0030P05.g1.613	48	164	613	chr3	123413539	**123413423**	-	0.977528
CH250_146n12TJ.835	327	777	835	chr3	130337767	130338217	+	0.967517
CH250_76e22TV.856	340	775	856	chr3	130433762	**130434197**	+	0.955399
CH250_76e22TJ.761	191	555	761	chr3	130587535	**130587171**	-	0.950413
RP43_135e8TV.601	536	601	601	chr3	131033097	131033162	+	1
RP41_0149I04.g1.705	88	141	705	chr3	141286604	141286657	+	0.981481
CH250_159p20TV.823	175	277	823	chr3	145673869	**145673971**	+	0.941748
CH250_159p20TJ.856	628	785	856	chr3	145804401	**145804244**	-	0.965517
CH250_71h12TJ.813	32	199	813	chr3	145959638	145959471	-	0.96732
CH255_16l5TV.214	1	53	214	chr3	147315214	147315162	-	1
RP41_0092E09.b1.758	122	584	758	chr3	147416412	147416874	+	0.946429
CH255_104d1TV.690	40	690	690	chr3	147472868	147472218	-	0.993243
CH255_12l18TV.302	5	302	302	chr3	150060820	**150061117**	+	0.995238
CH255_12l18TJ.246	37	246	246	chr3	150291864	**150291655**	-	0.97561
CH255_234l7TV.610	471	610	610	chr3	150354193	150354054	-	0.987805
CH250_35j16TV.735	1	106	735	chr3	151947609	151947504	-	0.975
CH250_80e20TV.805	88	260	805	chr3	151994280	151994108	-	0.952381
CH255_131p18TV.622	225	448	622	chr3	152362973	152363196	+	0.986301
CH255_131j12TV.611	2	435	611	chr3	152418578	152419011	+	0.992443
CH255_142n2TV.796	4	180	796	chr3	152498466	152498642	+	0.986928
CH255_131j12TJ.176	4	151	176	chr3	152635134	152634987	-	0.978261
CH250_156o15TV.851	27	445	851	chr3	156211009	**156211427**	+	0.974619
CH250_156o15TJ.845	67	131	845	chr3	156353601	**156353537**	-	0.966667
CH250_146d6TJ.865	611	786	865	chr3	156412771	156412596	-	0.927835
CH250_49l14TJ.840	605	801	840	chr3	156476220	156476024	-	0.952381
RP41_0070D05.b1.760	52	130	760	chr3	162404418	162404340	-	0.985075
RP43_0016I06.b1.719	71	349	719	chr3	163668651	163668929	+	0.974545
RP43_101l22TV.831	142	831	831	chr3	163735917	**163736606**	+	0.991379
RP43_0016I06.g1.638	41	434	638	chr3	163848196	**163847803**	-	0.994413
RP43_101l22TJ.916	1	916	916	chr3	163884896	163883981	-	0.993065
RP41_0147H17.g1.737	72	326	737	chr3	164111360	164111614	+	0.963855
RP41_5f12TV.806	108	302	806	chr3	166402164	**166402358**	+	0.941799
RP41_5f12TJ.531	457	528	531	chr3	166573735	**166573664**	-	1
RP41_31k22TJ.861	645	721	861	chr3	166604691	166604615	-	0.935065
RP41_0092B01.b1.774	133	539	774	chr3	167615616	167615210	-	0.94306
RP41_0072O21.b1.734	368	462	734	chr3	171311118	**171311212**	+	0.975904
RP41_0072O21.g1.712	310	548	712	chr3	171487517	**171487279**	-	0.981366
CH250_35i8TV.589	462	589	589	chr3	184329634	**184329761**	+	0.96875
CH250_35i8TJ.784	189	304	784	chr3	184485473	**184485358**	-	0.974138
CH250_171d9TV.861	711	861	861	chr3	184663076	184662926	-	0.969388

RP41_0095D21.g1.741	216	478	741	chr3	194755745	**194756007**	+	0.954887
RP41_0095D21.b1.760	66	586	760	chr3	194956351	**194955831**	-	0.978852
CH250_167l16TV.766	135	257	766	chr3	199022450	199022572	+	0.966942
CH255_140m10TJ.852	467	529	852	chr4	357377	357439	+	0.984127
RP41_0149N24.g1.730	45	223	730	chr4	8104509	8104687	+	0.911765
RP43_0168N15.b1.716	395	591	716	chr4	9369950	9369754	-	0.987179
CH255_12j19TV.136	1	136	136	chr4	16640503	16640638	+	1
CH255_142l19TV.182	4	182	182	chr4	16728935	**16729113**	+	0.992701
CH255_12j19TJ.111	1	111	111	chr4	16902323	**16902213**	-	0.916667
CH255_142l19TJ.350	9	350	350	chr4	16975297	16974956	-	0.97619
RP43_102o20TV.603	554	603	603	chr4	47261616	**47261665**	+	1
RP43_0084I05.b1.700	64	518	700	chr4	47347087	**47346633**	-	0.995169
RP43_102o20TJ.650	4	266	650	chr4	47384409	47384147	-	0.995671
RP43_0005I06.b1.618	108	232	618	chr4	47398889	47398765	-	0.982301
RP43_125g1TV.761	70	277	761	chr4	47547105	47547312	+	0.975962
RP43_0150O18.b1.725	319	553	725	chr4	47586710	47586944	+	0.985294
RP43_134o19TV.825	370	823	825	chr4	47671567	**47672020**	+	0.994737
RP43_125g1TJ.565	45	565	565	chr4	47706012	**47705492**	-	0.991404
RP43_0150O18.g1.631	492	552	631	chr4	47711364	47711304	-	1
RP43_0068F21.g1.608	254	454	608	chr4	47755053	47754853	-	1
RP43_134o19TJ.708	1	708	708	chr4	47825736	47825029	-	0.992793
RP41_0082E19.b1.723	107	516	723	chr4	55225856	55226265	+	0.954545
CH250_163p8TV.889	742	822	889	chr4	57464924	57465004	+	0.973333
CH250_45a22TJ.615	1	263	615	chr4	65396667	**65396929**	+	0.901288
CH250_17h22TJ.868	36	316	868	chr4	65451492	**65451212**	-	0.946058
CH250_45a22TV.835	304	388	835	chr4	65547959	65547875	-	0.952941
RP41_0055B22.b1.775	247	369	775	chr4	69109781	69109903	+	0.95935
RP41_46n6TJ.839	509	731	839	chr4	69118120	69118342	+	0.957944
RP41_0058L04.g1.738	340	625	738	chr4	69142310	**69142595**	+	0.958904
RP41_46n6TV.858	686	747	858	chr4	69284846	**69284785**	-	0.965517
RP41_0063O23.b1.740	92	166	740	chr4	69766811	69766737	-	0.973333
RP43_101f11TJ.684	269	495	684	chr4	72831043	72831269	+	1
RP43_0164L02.g1.606	249	473	606	chr4	72944918	**72945142**	+	0.98895
RP43_107e14TJ.841	21	196	841	chr4	73097454	**73097279**	-	0.992593
RP43_0164L02.b1.696	383	520	696	chr4	73109699	73109562	-	1
CH255_102c12TV.944	707	794	944	chr4	89108759	89108846	+	1
CH255_204g22TJ.682	182	278	682	chr4	89142965	89143061	+	1
CH255_144o12TJ.336	4	215	336	chr4	89306643	**89306854**	+	0.981132
CH255_204g22TV.720	1	321	720	chr4	89327927	**89327607**	-	0.992481
CH255_22d12TJ.386	325	386	386	chr4	89342222	89342161	-	1
CH255_102c12TJ.429	1	93	429	chr4	89343063	89342971	-	1
CH255_262m24TJ.526	19	107	526	chr4	89392017	89391929	-	0.986111
RP43_0166F19.b1.685	21	134	685	chr4	102607783	102607896	+	1
CH250_83i12TJ.755	452	507	755	chr4	105183806	105183861	+	0.982143
CH250_5f18TJ.674	568	658	674	chr4	105268047	105268137	+	0.95122
CH255_262i13TV.697	513	624	697	chr4	108731530	108731641	+	1
CH255_254p6TV.812	4	78	812	chr4	108803959	108804033	+	1
CH255_254p6TJ.832	741	797	832	chr4	109001444	109001388	-	1
CH255_130a16TJ.613	1	609	613	chr4	114939241	114939849	+	0.979021
CH255_32i15TJ.534	5	77	534	chr4	114963234	**114963306**	+	1
CH255_130a16TV.363	43	363	363	chr4	115142529	**115142209**	-	0.985019
CH255_32i15TV.268	5	268	268	chr4	115230041	115229778	-	0.994845

RP41_0072L13.g1.713	73	305	713	chr4	117015531	**117015763**	+	0.914027
RP41_0072L13.b1.729	370	470	729	chr4	117176081	**117175981**	-	0.967742
RP43_0084E08.b1.717	69	578	717	chr4	117269781	117270290	+	0.993039
RP43_0001I21.g1.664	38	327	664	chr4	117341952	**117342241**	+	0.989362
RP43_0084E08.g1.643	4	151	643	chr4	117419818	**117419671**	-	0.986111
RP43_0001I21.b1.711	77	425	711	chr4	117539050	117538702	-	0.972308
RP41_2j22TV.880	347	639	880	chr4	120133911	120134203	+	0.976109
RP43_0086O05.b1.649	76	185	649	chr4	120799106	120798997	-	0.990654
CH255_46g6TJ.807	6	273	807	chr4	127399530	127399797	+	1
CH255_8o4TV.539	46	539	539	chr4	127479789	**127480282**	+	0.977041
CH255_46g6TV.745	238	745	745	chr4	127624447	**127623940**	-	0.978339
CH255_8o4TJ.428	4	428	428	chr4	127662777	127662353	-	0.980707
CH255_227d16TJ.845	2	843	845	chr4	127754384	127755225	+	0.982484
CH255_196h23TJ.859	4	186	859	chr4	127834657	127834839	+	0.993377
CH255_198b5TV.305	4	131	305	chr4	127834712	**127834839**	+	1
CH255_227d16TV.740	664	740	740	chr4	127911790	**127911714**	-	1
CH255_198b5TJ.762	1	217	762	chr4	128055917	128055701	-	0.972678
CH250_158h15TJ.800	2	84	800	chr4	129306674	**129306756**	+	0.979592
RP41_0055D07.b1.769	406	496	769	chr4	129449914	129449824	-	1
CH250_158h15TV.887	402	500	887	chr4	129449922	**129449824**	-	0.968421
RP41_35I12TJ.873	519	606	873	chr4	129549058	129548971	-	0.953488
RP41_0052L15.b1.745	223	280	745	chr4	129558106	129558049	-	0.982759
CH250_15g22TJ.690	459	671	690	chr4	142210600	142210812	+	0.986111
CH255_241h10TV.725	351	400	725	chr4	155306974	155307023	+	1
CH255_234e3TJ.539	325	539	539	chr4	155385270	155385484	+	1
CH250_68e18TJ.829	743	798	829	chr4	160730292	160730347	+	1
CH250_147k1TJ.825	348	401	825	chr4	160773291	160773344	+	0.981481
CH250_47n1TJ.657	312	364	657	chr4	165936634	165936686	+	0.953488
CH250_67b12TV.725	501	581	725	chr4	165967540	165967620	+	0.961039
CH250_163j13TJ.840	241	666	840	chr4	165983702	165984127	+	0.969048
CH250_92c19TV.869	472	609	869	chr4	166001022	**166001159**	+	0.963768
CH250_163j13TV.860	228	312	860	chr4	166177156	**166177072**	-	0.929412
CH250_92c19TJ.836	277	388	836	chr4	166212489	166212378	-	0.991071
CH255_25k4TV.491	41	491	491	chr4	169285952	169286402	+	0.98916
CH255_109o18TJ.878	340	878	878	chr4	169333325	169333863	+	0.995565
CH255_114l7TJ.898	250	299	898	chr4	169440187	169440236	+	1
CH255_10j19TJ.645	5	465	645	chr4	169441899	169442359	+	0.983607
CH255_48f14TJ.814	764	814	814	chr4	169456101	**169456151**	+	1
CH255_25k4TJ.346	220	346	346	chr4	169516305	**169516179**	-	1
CH255_109o18TV.888	531	853	888	chr4	169518274	169517952	-	0.992727
CH255_10j19TV.398	4	187	398	chr4	169633268	169633085	-	0.993865
CH255_2l2TJ.525	2	172	525	chr4	176041556	176041726	+	0.99359
CH255_24b3TJ.142	1	50	142	chr4	176090527	176090576	+	1
CH255_24c22TJ.237	6	237	237	chr4	176100638	176100869	+	0.975
CH255_240h19TJ.842	4	464	842	chr4	176130053	176130513	+	0.993197
CH255_266a13TJ.841	732	841	841	chr4	176180119	**176180228**	+	1
CH255_2l2TV.450	384	450	450	chr4	176251446	**176251380**	-	1
CH255_240h19TV.520	419	520	520	chr4	176339067	176338966	-	0.984127
CH255_24c22TV.529	3	529	529	chr4	176355947	176355421	-	0.979943
CH255_228j23TV.834	1	64	834	chr4	176446260	176446197	-	1
CH250_50j21TV.794	149	264	794	chr4	179618647	179618762	+	0.982301
CH250_59m2TJ.188	20	188	188	chr4	179673256	179673424	+	0.928571

CH250_61l7TJ.580	4	375	580	chr4	179673256	179673627	+	0.926154
CH250_172e6TJ.824	221	300	824	chr4	179700210	179700289	+	0.9625
CH250_53h10TV.786	255	600	786	chr4	179725696	179726041	+	0.964912
CH250_53h10TJ.814	162	276	814	chr4	179906198	179906312	+	0.964912
CH250_172e6TV.835	332	443	835	chr4	179949427	179949538	+	0.955357
CH250_59m2TV.483	1	122	483	chr4	179964900	179965021	+	0.964706
CH250_61l7TV.146	39	146	146	chr4	179965023	179965130	+	0.984127
CH250_92b9TV.887	615	726	887	chr4	179968570	179968681	+	0.942308
CH255_102d9TJ.692	383	692	692	chr4	182150030	182150339	+	0.964467
CH255_206g6TV.627	6	619	627	chr4	182177152	182177765	+	0.968487
CH255_115b10TJ.815	4	524	815	chr4	182190349	182190869	+	0.988095
CH255_259o14TV.692	1	493	692	chr4	182278413	182278905	+	0.984979
CH255_194o14TJ.868	5	352	868	chr4	182330279	182329932	-	0.987013
CH255_206g6TJ.372	290	372	372	chr4	182347046	182346964	-	1
CH255_132i3TJ.638	1	547	638	chr4	182563843	182563297	-	0.992141
RP41_0072K10.g1.723	80	350	723	chr4	187167544	187167814	+	0.958491
RP41_0072K10.b1.750	33	134	750	chr4	187354523	187354422	-	0.974026
RP43_0078P13.g1.653	140	357	653	chr4	190891219	190891436	+	0.869159
RP43_0027N19.b1.743	286	365	743	chr4	190896588	190896509	-	0.9375
CH255_120o20TV.672	352	420	672	chr5	10123996	10124064	+	1
CH255_120o20TJ.442	1	442	442	chr5	10314571	10314130	-	0.978378
RP41_0086E09.b1.727	102	225	727	chr5	12194004	12194127	+	0.95935
RP41_0055G19.g1.747	567	664	747	chr5	12284160	12284257	+	1
RP41_0086E09.g1.735	283	384	735	chr5	12364015	12363914	-	0.961538
CH255_204i10TJ.675	589	675	675	chr5	13003001	13002915	-	1
RP43_0031C19.b1.718	79	332	718	chr5	18734478	18734731	+	0.995984
RP43_0076E11.g1.615	3	252	615	chr5	18761767	18762016	+	0.982456
RP43_128m9TV.774	1	50	774	chr5	18774615	18774664	+	1
RP43_0076E11.b1.663	107	459	663	chr5	18900040	18899688	-	0.997101
RP43_0031C19.g1.636	44	248	636	chr5	18923646	18923442	-	0.985507
RP43_128m9TJ.802	20	300	802	chr5	18938396	18938116	-	0.980237
RP43_0020O07.b1.706	55	230	706	chr5	18979990	18979815	-	0.988304
RP41_0095N02.g1.723	430	532	723	chr5	25652138	25652240	+	0.969697
RP41_0095N02.b1.758	44	243	758	chr5	25836698	25836499	-	0.956989
CH255_12h7TJ.371	1	189	371	chr5	42412606	42412794	+	0.99375
CH255_197a16TV.610	1	610	610	chr5	42537381	42537990	+	0.989717
CH255_204k20TV.708	1	197	708	chr5	42578104	42578300	+	0.993865
CH255_223a22TV.710	4	134	710	chr5	42613705	42613835	+	1
CH255_223a22TJ.880	548	781	880	chr5	42812931	42812698	-	0.990338
RP43_0013O03.g1.627	193	253	627	chr5	42924921	42924981	+	1
RP43_0013O03.b1.731	41	615	731	chr5	43096259	43095685	-	0.997669
CH255_140f9TJ.832	372	457	832	chr5	43432975	43433060	+	1
CH255_140f9TV.808	612	756	808	chr5	43629568	43629424	-	0.980769
CH255_199e9TV.745	694	745	745	chr5	44433373	44433424	+	1
CH255_199e21TV.802	741	802	802	chr5	44433373	44433434	+	1
CH255_198b8TV.778	1	480	778	chr5	44477177	44477656	+	0.983333
CH255_226n23TV.904	5	904	904	chr5	44488522	44489421	+	0.980882
CH255_37g5TV.775	711	775	775	chr5	44589862	44589926	+	1
CH255_26c11TV.138	1	88	138	chr5	44598018	44598105	+	0.956522
CH255_199e21TJ.716	554	716	716	chr5	44676720	44676558	-	1
CH255_199e9TJ.617	510	617	617	chr5	44676720	44676613	-	1
CH255_198b8TJ.865	299	403	865	chr5	44677037	44676933	-	1

CH255_37g5TJ.608	1	608	608	chr5	44816742	44816135	-	0.9869
CH255_26c11TJ.360	7	360	360	chr5	44915475	44915122	-	0.996753
CH250_48j18TV.802	708	802	802	chr5	51396860	**51396954**	+	1
CH250_48j18TJ.617	214	354	617	chr5	51537551	**51537411**	-	0.945736
RP43_0164J10.b1.692	76	259	692	chr5	57018261	**57018444**	+	1
CH255_18e9TJ.210	67	210	210	chr5	57205352	57205209	-	0.984848
RP43_0164J10.g1.591	9	468	591	chr5	57207723	**57207264**	-	0.981752
RP43_100g24TV.648	485	648	648	chr5	57270151	57269988	-	1
CH250_36f16TJ.402	69	186	402	chr5	74323811	74323928	+	0.95
CH250_9a24TJ.650	190	486	650	chr5	74343872	74344168	+	0.94
RP43_120o12TV.373	227	373	373	chr5	74809073	74808927	-	0.983871
RP43_0173K20.b1.703	411	595	703	chr5	76487386	76487202	-	0.976471
CH255_38c19TV.323	1	323	323	chr5	82230752	**82231074**	+	0.98773
CH255_38c19TJ.316	10	316	316	chr5	82441978	**82441672**	-	0.987261
CH255_248c9TJ.838	13	838	838	chr5	93600893	93601718	+	0.993421
CH255_143f15TJ.850	3	849	850	chr5	93640228	**93641074**	+	0.991117
CH255_143f15TV.796	537	762	796	chr5	93864364	**93864139**	-	0.994792
CH255_128b10TJ.854	333	830	854	chr5	94626422	94626919	+	0.988739
CH255_214g4TV.722	3	722	722	chr5	94630223	94630942	+	0.990775
CH255_19p24TV.531	54	531	531	chr5	94767988	94768465	+	0.98538
CH255_97k3TV.139	41	139	139	chr5	94778527	**94778625**	+	0.985075
CH255_121k17TJ.694	1	669	694	chr5	94805910	**94805242**	-	0.989983
RP43_0047P05.g1.650	298	572	650	chr5	94823212	94823486	+	1
CH255_128b10TV.504	395	504	504	chr5	94834876	94834767	-	0.983871
RP43_0044A24.g1.619	170	356	619	chr5	94846920	94847106	+	0.994624
CH255_130k10TJ.837	186	292	837	chr5	94873782	94873676	-	1
RP43_0147L10.g1.612	180	318	612	chr5	94909320	**94909458**	+	0.992806
CH255_228g16TJ.855	625	855	855	chr5	94940164	94939934	-	0.986301
CH255_19p24TJ.419	169	419	419	chr5	94981158	94980908	-	1
RP43_0044A24.b1.721	113	533	721	chr5	94981278	**94980858**	-	0.994382
RP43_0047P05.b1.732	62	205	732	chr5	94988373	94988230	-	0.954887
CH255_132c14TV.686	591	683	686	chr5	95034810	95034718	-	1
RP43_0147L10.b1.677	80	574	677	chr5	95062053	95061559	-	0.995272
CH250_175n18TJ.708	621	707	708	chr5	107085363	**107085449**	+	0.96875
CH250_175n18TV.850	575	850	850	chr5	107237929	**107237654**	-	0.988281
CH250_29j6TJ.850	3	427	850	chr5	107264614	107264190	-	0.949761
RP43_104n5TJ.699	1	294	699	chr5	108086947	108086654	-	0.99095
CH255_37j1TJ.601	544	601	601	chr5	127842807	**127842864**	+	1
CH255_37j1TV.625	243	625	625	chr5	128027808	**128027426**	-	0.984733
CH255_268p21TJ.814	1	311	814	chr5	130594166	130593856	-	0.996241
CH250_147g13TV.798	4	245	798	chr5	133686851	133687092	+	0.95098
CH250_74n21TV.886	321	384	886	chr5	133699066	**133699129**	+	0.984375
CH250_74n21TJ.814	358	814	814	chr5	133860911	**133860455**	-	0.961111
CH250_147g13TJ.752	43	133	752	chr5	133869030	133868940	-	0.970149
RP41_29b12TJ.827	635	776	827	chr5	141439057	**141439198**	+	0.961832
RP41_29b12TV.857	494	627	857	chr5	141610014	**141609881**	-	0.992537
CH250_148n10TV.700	522	661	700	chr5	151719782	151719921	+	1
CH255_258i10TJ.826	721	816	826	chr5	153466653	153466748	+	0.987952
RP43_116e9TJ.591	411	591	591	chr5	156088389	156088569	+	1
RP43_0013O06.g1.647	392	476	647	chr5	156120174	156120258	+	0.947368
RP43_0072F17.g1.645	401	485	645	chr5	156120174	**156120258**	+	0.965517
RP43_0013O06.b1.715	358	577	715	chr5	156291351	**156291132**	-	0.975

RP43_0072F17.b1.694	190	673	694	chr5	156300627	156300144	-	0.995595
RP41_24e19TV.896	33	128	896	chr5	162580725	162580820	+	0.959459
RP41_0056K19.b1.777	257	353	777	chr5	162667730	162667826	+	0.979381
RP41_0056K19.g1.729	464	597	729	chr5	162819075	162818942	-	0.8
RP41_0071F07.g1.712	120	264	712	chr5	175648091	**175648235**	+	0.943262
RP41_18d21TJ.799	1	175	799	chr5	175822171	**175821997**	-	0.926667
RP41_0071F07.b1.744	81	380	744	chr5	175823812	175823513	-	0.952703
CH255_2i7TJ.469	4	228	469	chr6	11553348	11553572	+	0.975758
CH255_124e9TV.804	724	804	804	chr6	11639504	11639584	+	1
CH255_218a16TV.672	603	672	672	chr6	11641750	11641819	+	1
CH255_194c15TJ.255	1	255	255	chr6	11676792	11677046	+	0.990909
CH255_206i24TV.395	313	388	395	chr6	11687661	11687736	+	1
CH255_265f11TJ.579	118	306	579	chr6	11700150	11700338	+	0.983784
CH255_198n17TJ.411	10	411	411	chr6	11702455	11702856	+	0.978261
CH255_1o2TV.396	241	290	396	chr6	11719337	11719386	+	0.98
CH255_218l19TJ.822	45	764	822	chr6	11758714	11759433	+	0.992151
CH255_236j3TJ.384	289	384	384	chr6	11847301	**11847396**	+	1
CH255_124e9TJ.728	659	728	728	chr6	11855148	**11855079**	-	1
CH255_218a16TJ.620	41	620	620	chr6	11879446	11878867	-	0.989775
CH255_140d2TV.353	3	345	353	chr6	11883028	11883370	+	0.986842
CH255_206i24TJ.478	36	478	478	chr6	11937452	11937010	-	0.982935
CH255_218l19TV.791	2	791	791	chr6	11941212	11940423	-	0.984043
CH255_1o2TJ.264	1	264	264	chr6	11956741	11956478	-	0.985294
CH255_140d2TJ.745	156	270	745	chr6	12112243	12112129	-	0.991304
RP43_0044H15.g1.646	128	276	646	chr6	13692044	**13692192**	+	0.986577
RP43_0044H15.b1.726	85	199	726	chr6	13873416	**13873302**	-	1
RP41_0072L10.g1.699	128	233	699	chr6	26514759	26514864	+	0.933962
RP41_0056P10.b1.767	226	540	767	chr6	26522443	26522757	+	0.935018
RP41_35k10TJ.862	215	529	862	chr6	26522443	**26522757**	+	0.938907
RP41_7h17TV.774	383	572	774	chr6	26703064	**26702875**	-	0.952632
RP41_0056P10.g1.700	86	236	700	chr6	26710440	26710290	-	0.944828
RP41_35k10TV.869	353	628	869	chr6	26728284	26728009	-	0.941818
CH255_120p20TJ.646	1	646	646	chr6	27400436	**27401081**	+	0.994141
CH255_4f14TV.193	1	190	193	chr6	27527731	**27527542**	-	1
CH255_244a19TJ.746	22	746	746	chr6	27528495	27527771	-	1
CH255_120p20TV.491	1	187	491	chr6	27620516	27620330	-	0.984733
CH255_244l12TJ.427	139	240	427	chr6	27659214	27659113	-	0.980198
RP43_0022F16.b1.696	72	259	696	chr6	38304912	**38305099**	+	0.994595
RP43_0022F16.g1.607	47	449	607	chr6	38491644	**38491242**	-	1
RP43_0036M08.b1.717	76	546	717	chr6	38509003	38508533	-	0.997602
RP41_0086A23.g1.702	292	369	702	chr6	41367478	**41367555**	+	0.968254
RP41_0086A23.b1.737	60	199	737	chr6	41549458	**41549319**	-	0.968504
RP43_0016N01.b1.713	487	593	713	chr6	46865545	46865439	-	1
CH255_7l16TV.207	112	207	207	chr6	47109406	47109501	+	1
CH255_240j4TV.680	140	304	680	chr6	52623291	52623455	+	1
CH255_250l20TV.632	82	632	632	chr6	52786491	**52787041**	+	0.982301
CH255_240j4TJ.757	253	591	757	chr6	52842810	**52842472**	-	0.972561
CH255_250l20TJ.706	17	270	706	chr6	52968653	52968400	-	0.986667
RP43_0008F11.g1.632	39	478	632	chr6	80197221	**80197660**	+	0.99422
RP43_0008F11.b1.649	34	279	649	chr6	80395840	**80395595**	-	0.995327
CH250_33h20TJ.463	257	341	463	chr6	87149029	**87149113**	+	0.985294
CH250_33h20TV.702	139	446	702	chr6	87336390	**87336083**	-	0.927393

CH255_200j10TV.749	531	749	749	chr6	97818152	**97818370**	+	1
CH255_200j10TJ.767	4	79	767	chr6	98048059	**98047984**	-	1
RP41_43o10TV.821	95	365	821	chr6	104530624	104530894	+	0.943396
RP41_0065K02.b1.735	76	468	735	chr6	104564621	**104565013**	+	0.960733
RP41_43o10TJ.163	1	139	163	chr6	104701624	**104701486**	-	0.966667
RP41_0065K02.g1.721	194	272	721	chr6	104725155	104725077	-	0.962025
RP41_14c14TJ.835	242	426	835	chr6	106868361	**106868545**	+	0.961326
RP41_14c14TV.532	33	492	532	chr6	107047185	**107046726**	-	0.958716
RP43_0150P07.b1.703	73	608	703	chr6	123957441	**123957976**	+	0.993007
RP43_0150P07.g1.617	4	493	617	chr6	124137282	**124136793**	-	0.995146
RP43_0019G06.g1.628	330	449	628	chr6	125231395	**125231514**	+	1
RP43_0019G06.b1.704	388	577	704	chr6	125284267	**125284078**	-	1
RP41_0075G02.b1.682	132	364	682	chr6	138878856	**138879088**	+	0.973913
RP41_0075G02.g1.693	281	523	693	chr6	139053032	**139052790**	-	0.97006
CH250_162l15TJ.775	11	155	775	chr6	141562839	141562695	-	0.894309
CH250_77i14TV.510	39	510	510	chr6	144590457	144589986	-	0.957447
CH250_39f11TJ.228	151	201	228	chr6	145432735	145432685	-	1
CH250_147i7TV.376	9	67	376	chr6	151635871	**151635929**	+	1
CH250_147i7TJ.708	259	341	708	chr6	151847502	**151847420**	-	0.982456
CH250_152e1TJ.713	641	713	713	chr6	151861688	151861616	-	1
CH250_43o17TV.580	435	571	580	chr6	152973878	**152974014**	+	0.962687
CH250_43o17TJ.837	535	710	837	chr6	153152973	**153152798**	-	0.95
CH250_174p9TV.670	374	552	670	chr6	153227388	153227210	-	1
RP41_44d4TJ.863	1	103	863	chr6	153702165	**153702267**	+	0.954023
RP41_0076C08.b1.720	205	381	720	chr6	153889576	**153889400**	-	0.932203
RP41_0076E06.b1.634	191	324	634	chr6	153889576	153889443	-	0.918605
RP41_0074B05.b1.750	209	336	750	chr6	153889576	153889449	-	0.9375
RP43_111f4TV.127	34	117	127	chr6	161193964	161194047	+	1
CH250_3l15TV.612	437	568	612	chr7	5476685	**5476816**	+	0.949153
CH250_3l15TJ.391	4	59	391	chr7	5669960	**5669905**	-	0.90625
CH255_214l3TV.236	78	151	236	chr7	5721750	5721677	-	1
RP41_0155D05.g1.731	44	262	731	chr7	7457133	7456915	-	0.931579
RP43_0078B18.g1.642	48	143	642	chr7	9236304	9236399	+	0.988372
RP43_0042A22.b1.715	213	463	715	chr7	10845701	10845951	+	0.991304
RP43_131n10TJ.848	360	848	848	chr7	10866333	**10866821**	+	0.980907
RP43_0042A22.g1.637	4	472	637	chr7	10996820	**10996352**	-	0.982097
RP43_131n10TV.862	1	50	862	chr7	11022073	11022024	-	1
RP41_0087H20.g1.745	76	261	745	chr7	14424311	**14424496**	+	0.945055
RP41_0087H20.b1.694	116	256	694	chr7	14587967	**14587827**	-	0.943262
CH255_108d8TJ.643	1	82	643	chr7	14601980	14601899	-	1
RP43_0030K12.b1.629	45	398	629	chr7	22529458	22529811	+	0.996564
RP43_0088K21.b1.703	38	314	703	chr7	22573555	22573831	+	0.991304
RP43_0023F10.g1.643	4	488	643	chr7	22594014	22594498	+	0.997326
RP43_0157B04.b1.715	147	568	715	chr7	22594080	22594501	+	0.994186
RP43_0148I15.b1.723	149	619	723	chr7	22594080	**22594550**	+	0.997085
RP43_0038F15.g1.639	1	415	639	chr7	22663136	**22662722**	-	1
RP43_0148I15.g1.627	435	528	627	chr7	22726500	22726407	-	1
RP43_0023F10.b1.654	62	374	654	chr7	22774567	22774255	-	0.977612
CH255_140k16TJ.860	754	827	860	chr7	36220767	36220840	+	1
CH255_266e22TJ.743	3	56	743	chr7	36309706	**36309759**	+	1
RP43_0020H17.g1.666	199	548	666	chr7	36309987	36310336	+	0.979695
RP43_119p2TJ.764	667	764	764	chr7	36310772	36310869	+	1

RP43_127m1TV.322	1	321	322	chr7	36330513	36330833	+	0.985185
RP43_0021K18.g1.641	196	334	641	chr7	36346059	36346197	+	1
RP43_98d17TV.803	54	213	803	chr7	36353597	36353756	+	1
CH255_266e22TV.770	284	770	770	chr7	36506565	36506079	-	0.990826
RP43_122d3TJ.889	692	889	889	chr7	39447828	39448025	+	1
RP43_0145B02.g1.609	258	607	609	chr7	39583406	39583755	+	0.994048
RP43_122d3TV.526	180	526	526	chr7	39627957	39627611	-	0.996528
RP43_0039B14.g1.631	10	74	631	chr7	39640701	39640765	+	1
RP43_127b16TJ.756	1	128	756	chr7	39670765	39670638	-	1
RP43_0145B02.b1.682	121	307	682	chr7	39708197	39708011	-	0.97861
RP43_0086O05.g1.625	2	304	625	chr7	39714246	39713944	-	1
CH255_202k13TV.579	166	579	579	chr7	39874303	39874716	+	1
RP41_0088E12.g1.723	467	581	723	chr7	39945430	39945544	+	1
CH255_202k13TJ.566	419	563	566	chr7	40092429	40092285	-	1
RP41_0088E12.b1.746	335	447	746	chr7	40105977	40105865	-	0.983607
CH255_248m2TJ.838	5	125	838	chr7	42507290	42507410	+	1
CH255_44a12TV.694	2	428	694	chr7	42610164	42610590	+	0.98806
CH255_44a12TJ.284	1	284	284	chr7	42794445	42794162	-	1
CH255_206g21TV.637	478	637	637	chr7	42804915	42804756	-	0.987179
RP43_0062I09.b1.731	45	632	731	chr7	48787250	48787837	+	0.984513
RP43_0170L05.b1.686	416	680	686	chr7	48795358	48795622	+	1
RP43_0033P19.b1.723	476	544	723	chr7	48820058	48820126	+	1
RP43_0171O10.g1.615	51	571	615	chr7	48850375	48850895	+	1
RP43_0171M24.g1.621	486	561	621	chr7	48850812	48850887	+	1
RP43_0019H06.b1.733	417	576	733	chr7	48935895	48935736	-	0.985075
RP43_0166P23.g1.624	6	498	624	chr7	48940075	48939583	-	0.995122
RP43_0062I09.g1.642	470	532	642	chr7	48946636	48946574	-	1
RP43_0033P19.g1.634	48	495	634	chr7	48978856	48978409	-	0.993127
RP43_0171M24.b1.702	140	519	702	chr7	49040521	49040142	-	0.97479
CH255_128k13TJ.853	3	853	853	chr7	54234046	54234896	+	0.978008
CH255_266e2TV.542	1	542	542	chr7	54372322	54372863	+	0.973783
CH255_128k13TV.529	91	526	529	chr7	54453499	54453064	-	0.990148
CH255_266e2TJ.386	16	200	386	chr7	54610439	54610255	-	0.975904
RP43_0170B11.g1.628	97	539	628	chr7	55915790	55916232	+	0.971311
RP43_0039B14.b1.732	59	422	732	chr7	55928624	55928987	+	0.991124
RP43_0170B11.b1.730	492	647	730	chr7	56217713	56217558	-	1
RP43_0152H23.g1.594	9	277	594	chr7	56220505	56220237	-	0.983936
RP43_0066J07.g1.608	1	259	608	chr7	56275965	56275707	-	0.995935
RP41_0062J01.b1.770	427	572	770	chr7	56503437	56503582	+	0.965909
CH255_136m8TJ.419	303	411	419	chr7	57373644	57373536	-	1
RP41_40p12TV.851	161	231	851	chr7	57464951	57465021	+	0.971831
RP41_10j22TJ.650	368	444	650	chr7	62724763	62724687	-	0.987013
CH255_258c2TJ.778	178	275	778	chr7	63582747	63582844	+	1
CH255_258c2TV.746	5	721	746	chr7	63851115	63850399	-	0.989489
RP43_0027C06.b1.705	114	345	705	chr7	64678508	64678277	-	0.974138
RP41_42b3TJ.737	180	235	737	chr7	67331579	67331524	-	0.981132
RP41_44d9TJ.580	336	488	580	chr7	68837930	68838082	+	0.954248
RP41_44d9TV.874	12	524	874	chr7	69011410	69010898	-	0.982869
RP41_13k1TV.832	277	661	832	chr7	69119045	69118661	-	0.981333
RP41_0067P17.b1.757	74	162	757	chr7	96214136	96214224	+	0.963415
RP43_133c17TV.872	39	159	872	chr7	97145323	97145443	+	1
CH250_6i21TJ.377	115	332	377	chr7	98814065	98814282	+	0.9801

CH250_83m4TJ.811	287	429	811	chr7	98905127	98905269	+	0.937063
CH250_146h14TJ.860	397	648	860	chr7	98971050	**98971301**	+	0.964143
CH250_83m4TV.850	22	145	850	chr7	98998975	**98998852**	-	0.946809
CH250_71l15TJ.796	1	52	796	chr7	99002377	**99002428**	+	0.875
CH250_146h14TV.875	451	504	875	chr7	99030005	**99029952**	-	0.944444
CH250_71l15TV.845	714	845	845	chr7	99041034	99040903	-	0.949367
RP41_0090A10.b1.774	442	598	774	chr7	129997149	129996993	-	1
RP41_0064M14.g1.688	379	482	688	chr7	132537037	**132537140**	+	1
RP41_0064M14.b1.776	78	246	776	chr7	132724926	**132724758**	-	0.969697
CH255_102d11TJ.720	33	95	720	chr7	133650073	133650135	+	0.968254
RP43_0006K06.g1.653	81	170	653	chr7	136964659	136964748	+	1
CH250_156n24TJ.824	203	281	824	chr7	136964670	136964748	+	0.961538
CH250_75e3TV.885	685	848	885	chr7	136996075	136996238	+	0.967742
RP43_0090K24.b1.696	93	185	696	chr7	137057887	**137057979**	+	0.988235
RP43_0006K06.b1.679	319	518	679	chr7	137158939	**137158740**	-	0.987952
RP43_0090K24.g1.631	50	419	631	chr7	137225586	137225217	-	0.979104
RP41_0059J12.g1.740	451	592	740	chr7	147472455	**147472596**	+	0.975
RP41_0059J12.b1.770	384	609	770	chr7	147632236	**147632011**	-	0.948454
RP41_18e23TJ.849	470	687	849	chr7	148921764	148921547	-	0.990741
RP41_0066G14.g1.729	75	500	729	chr7	148928150	148927725	-	0.966759
CH250_148g1TV.845	1	336	845	chr7	148938537	148938202	-	0.965035
CH250_175o11TJ.759	428	632	759	chr7	151140128	151140332	+	0.950495
CH255_127i6TV.765	114	186	765	chr7	153146254	153146182	-	0.955224
CH255_240d24TJ.630	503	630	630	chr8	2010244	**2010371**	+	1
CH255_240d24TV.390	305	375	390	chr8	2192498	**2192428**	-	1
RP43_116p21TJ.816	702	816	816	chr8	7149738	7149852	+	1
CH255_122k5TJ.450	1	165	450	chr8	8695689	**8695853**	+	0.98374
CH255_122k5TV.343	8	227	343	chr8	8893034	**8892815**	-	0.968421
RP43_0078F03.g1.634	91	244	634	chr8	12264885	12264732	-	0.968
RP43_0145P18.g1.623	397	510	623	chr8	26768290	**26768403**	+	1
RP43_0145P18.b1.701	41	538	701	chr8	26912157	**26911660**	-	0.990544
CH255_224i22TV.649	1	636	649	chr8	38405642	**38406277**	+	0.995789
CH255_224i22TJ.582	450	582	582	chr8	38638965	**38638833**	-	1
RP41_0074G24.g1.709	129	341	709	chr8	40794667	40794879	+	0.942857
RP41_0076D18.g1.784	336	441	784	chr8	41011291	41011186	-	0.94
RP41_0077D18.g1.758	336	441	758	chr8	41011291	41011186	-	0.941176
CH255_128o14TJ.818	1	421	818	chr8	41542650	41542230	-	0.989446
RP41_47n17TJ.842	749	807	842	chr8	42541627	42541685	+	1
RP41_1n14TJ.766	1	375	766	chr8	42608767	42609141	+	0.974138
RP41_0096G13.b1.751	227	306	751	chr8	42613728	**42613807**	+	0.9875
RP41_47n17TV.861	1	107	861	chr8	42710122	**42710016**	-	0.988095
RP41_1n14TV.600	23	124	600	chr8	42763499	42763398	-	0.9625
RP41_34c11TJ.866	578	715	866	chr8	42935906	42936043	+	0.971014
CH250_163e4TV.889	585	732	889	chr8	42935906	42936053	+	0.966216
CH250_91j11TJ.711	1	69	711	chr8	49023491	**49023559**	+	1
CH250_91j11TV.877	249	305	877	chr8	49164540	**49164484**	-	0.982456
RP41_0081F21.g1.764	70	132	764	chr8	52111951	52111889	-	0.980392
RP41_5d12TJ.569	19	179	569	chr8	52171240	52171080	-	0.934783
RP43_0034L19.b1.724	57	341	724	chr8	54469559	54469843	+	0.98513
RP43_121k9TV.593	148	593	593	chr8	54472114	54472559	+	0.979661
RP43_101e21TJ.793	302	604	793	chr8	54527199	54527501	+	0.996516
RP43_0016O08.g1.649	253	435	649	chr8	54607196	54607378	+	0.985915

RP43_0040G02.b1.740	56	511	740	chr8	54636114	**54636569**	+	0.991781
RP43_101e21TV.604	3	604	604	chr8	54679252	**54678651**	-	0.993724
RP43_0040G02.g1.638	83	502	638	chr8	54777403	54776984	-	0.990712
RP43_0016O08.b1.696	81	557	696	chr8	54778046	54777570	-	0.994667
CH255_118b4TJ.748	685	748	748	chr8	55983269	**55983332**	+	1
CH255_118b4TV.771	676	771	771	chr8	56163263	**56163168**	-	1
CH255_46g17TJ.722	637	722	722	chr8	56430979	56431064	+	1
CH255_234l5TV.691	474	666	691	chr8	56518282	56518474	+	0.992126
CH250_170c19TJ.793	292	554	793	chr8	56519371	56519633	+	0.931559
CH250_21i17TJ.857	290	552	857	chr8	56519371	56519633	+	0.945274
CH250_174b17TJ.679	606	669	679	chr8	56829563	56829500	-	1
CH255_214m7TJ.653	338	653	653	chr8	59283950	59284265	+	0.990291
CH255_206b14TJ.656	314	656	656	chr8	59283950	59284292	+	0.990566
CH255_206b3TJ.645	79	244	645	chr8	59376996	59377161	+	0.993548
CH255_30a20TV.265	1	265	265	chr8	59378378	59378642	+	0.975962
CH255_256n9TV.828	4	86	828	chr8	59413340	59413422	+	1
CH255_240k11TJ.729	481	581	729	chr8	59450017	**59450117**	+	1
CH255_214m7TV.842	486	842	842	chr8	59476197	**59475841**	-	1
CH255_206b14TV.575	432	575	575	chr8	59476197	59476054	-	1
CH255_270a1TV.181	1	181	181	chr8	59504882	59504702	-	0.964789
CH255_30a20TJ.308	227	308	308	chr8	59565396	59565315	-	1
CH255_256n9TJ.829	1	298	829	chr8	59620406	59620109	-	0.992857
CH250_TV.829	385	436	829	chr8	71101361	71101310	-	0.972222
CH255_48i18TV.742	2	108	742	chr8	82876080	**82876186**	+	0.988636
CH255_205m6TJ.828	643	828	828	chr8	83076938	**83076753**	-	0.983607
CH255_48i18TJ.795	249	521	795	chr8	83082593	83082321	-	0.992674
CH250_19n14TJ.873	798	871	873	chr8	95072915	95072842	-	0.970588
RP41_8c10TJ.705	101	245	705	chr8	100169674	**100169818**	+	0.976562
RP41_0077O02.g1.753	77	471	753	chr8	100386392	**100385998**	-	0.93617
RP41_0060C18.g1.746	407	604	746	chr8	100402159	100401962	-	0.930233
CH250_1n2TV.682	1	169	682	chr8	114748140	114748308	+	0.953642
CH250_18l14TV.665	2	239	665	chr8	114881280	**114881517**	+	0.955556
CH250_1n2TJ.878	754	806	878	chr8	114933038	**114932986**	-	1
CH250_18l14TJ.752	167	509	752	chr8	115093000	115092658	-	0.967552
RP41_0064C10.g1.716	261	401	716	chr8	125023263	125023123	-	0.964539
RP43_0012M17.g1.642	409	465	642	chr8	130976702	**130976758**	+	1
RP43_0012M17.b1.727	467	608	727	chr8	131132594	**131132453**	-	1
RP41_0071B10.b1.756	64	205	756	chr8	132432535	132432394	-	0.952
CH255_126j4TV.198	1	198	198	chr8	133745124	133744927	-	0.977273
RP41_42g16TJ.769	192	275	769	chr8	146282822	146282905	+	0.916667
CH255_113f17TJ.753	589	753	753	chr9	14915133	14915297	+	0.991525
CH255_205a5TV.356	87	261	356	chr9	15032530	15032704	+	0.990909
CH255_205a5TJ.704	13	187	704	chr9	15257795	15257621	-	0.988304
RP41_0070F13.g1.698	94	531	698	chr9	15263927	15264364	+	0.94081
CH255_18g11TV.265	3	87	265	chr9	15285450	15285366	-	1
CH255_130k3TJ.818	5	692	818	chr9	17136875	17137562	+	0.989097
CH255_193n24TV.380	175	276	380	chr9	17146579	17146680	+	1
CH255_138b11TJ.739	26	739	739	chr9	17207472	17208185	+	0.986799
CH255_138i7TJ.827	1	797	827	chr9	17207473	**17208269**	+	0.989348
CH255_104k10TV.793	3	344	793	chr9	17305702	**17305361**	-	0.987302
CH255_130k3TV.462	1	194	462	chr9	17367157	17366964	-	0.98773
RP41_34n21TJ.867	761	855	867	chr9	25895934	**25896028**	+	1

RP41_34n21TV.867	467	551	867	chr9	26091240	**26091156**	-	0.964286
RP41_18n20TV.901	532	633	901	chr9	33566935	33567036	+	0.969388
CH255_108f7TJ.212	44	100	212	chr9	43862971	43863027	+	1
RP43_107f14TJ.729	1	80	729	chr9	44260180	44260259	+	1
RP43_0048G11.b1.778	80	350	778	chr9	44328056	44328326	+	0.992481
CH255_125o10TJ.628	20	155	628	chr9	62592268	62592403	+	0.962963
CH250_27m2TV.817	27	83	817	chr9	62753562	62753618	+	1
RP43_0048G11.g1.647	1	536	647	chr9	63363104	63362569	-	0.987654
RP43_0016N01.g1.620	183	382	620	chr9	65606309	65606110	-	1
RP43_119e10TV.222	122	196	222	chr9	65614125	65614051	-	1
CH255_144f13TV.320	102	320	320	chr9	70731667	70731885	+	0.993007
CH255_218k17TJ.727	11	594	727	chr9	70732356	70732939	+	0.983425
CH255_133f22TJ.854	710	795	854	chr9	70764595	**70764680**	+	0.981132
CH255_144f13TJ.644	541	643	644	chr9	70900867	**70900765**	-	1
CH255_133f22TV.623	6	99	623	chr9	70948668	70948575	-	1
CH255_218k17TV.664	460	592	664	chr9	70949562	70949430	-	0.987654
RP41_23n12TV.823	132	289	823	chr9	81155388	81155545	+	0.954545
RP41_0087O13.b1.742	77	143	742	chr9	81192966	**81193032**	+	1
RP41_23n12TJ.735	304	452	735	chr9	81335024	**81334876**	-	1
RP41_0087O13.g1.722	289	351	722	chr9	81381204	81381142	-	1
RP41_10h8TV.855	389	494	855	chr9	81438322	81438217	-	0.942308
RP41_18d7TJ.837	433	696	837	chr9	88209117	**88209380**	+	0.942966
CH250_150f13TJ.843	1	84	843	chr9	88246399	**88246482**	+	0.961538
RP41_18d7TV.767	587	767	767	chr9	88419834	**88419654**	-	0.966292
CH250_170p12TJ.842	681	782	842	chr9	88525961	**88525860**	-	0.988506
RP41_32f7TV.883	576	627	883	chr9	95016489	95016540	+	0.980769
CH255_217b23TJ.765	181	237	765	chr9	95398615	95398559	-	1
RP41_2f19TJ.839	89	177	839	chr9	114851347	**114851435**	+	0.962963
RP41_2f19TV.867	351	461	867	chr9	115002456	**115002346**	-	0.972973
RP41_0069C08.b1.742	128	266	742	chr9	117171916	**117172054**	+	0.977099
RP41_0074O18.g1.672	223	371	672	chr9	117340909	**117340761**	-	0.929688
RP41_0069C08.g1.726	80	222	726	chr9	117341053	117340911	-	0.940741
CH250_37p15TJ.569	226	289	569	chr9	123316937	123317000	+	1
RP41_0159F09.b1.751	329	441	751	chr9	125570296	**125570408**	+	0.944444
RP41_0159F09.g1.737	62	334	737	chr9	125756345	**125756073**	-	0.950758
RP41_0081J20.g1.748	268	336	748	chr9	132145437	132145369	-	1
RP41_0081O20.g1.750	153	214	750	chr9	135031911	135031972	+	0.983871
RP43_132g17TJ.604	5	292	604	chrUn_rar	1136673	1136386	-	0.974074
RP43_126n22TV.909	19	136	909	chrUn_rar	1146443	1146326	-	0.965909
RP43_0048F07.b1.754	110	607	754	chrUn_rar	1148823	1148326	-	0.983146
RP43_129h4TJ.607	74	607	607	chrUn_rar	1187347	1186814	-	0.978858
RP43_0002M16.b1.703	125	508	703	chrUn_rar	1214090	1213707	-	0.979839
RP43_0038H05.g1.613	304	436	613	chrUn_rar	1798815	1798947	+	1
RP41_0095J08.b1.762	64	450	762	chrX	5803536	5803150	-	0.972125
RP43_124n9TV.478	410	463	478	chrX	6625809	6625756	-	1
RP43_0010O12.g1.642	452	515	642	chrX	8227172	8227109	-	1
RP43_0088K21.g1.637	466	536	637	chrX	20166467	20166397	-	1
RP41_0063O23.g1.729	193	293	729	chrX	23248134	23248234	+	0.950495
RP43_105e11TJ.796	1	796	796	chrX	33338580	**33339375**	+	0.993506
RP43_0021M15.b1.738	73	160	738	chrX	33340953	**33340866**	-	0.987179
RP43_0076G03.b1.668	74	455	668	chrX	33405803	33405422	-	0.99697
RP43_105e11TV.552	44	213	552	chrX	33506435	33506266	-	0.993631

CH250_163a22TV.879	310	423	879	chrX	37250069	37249956	-	0.990476
RP43_110e1TJ.838	6	759	838	chrX	41772990	41772237	-	0.998454
RP41_22i2TJ.834	1	190	834	chrX	48356152	**48356341**	+	0.986667
RP41_22i2TV.847	304	445	847	chrX	48511760	**48511619**	-	0.964789
CH255_254p15TV.656	1	85	656	chrX	71430161	71430245	+	1
CH255_256k18TV.788	4	87	788	chrX	71430162	71430245	+	1
RP41_0092E04.b1.772	240	560	772	chrX	82369872	82369552	-	0.919048
CH255_32k21TJ.150	1	150	150	chrX	82763748	82763897	+	0.973684
CH255_208o3TV.545	4	366	545	chrX	82798015	82798377	+	0.980315
CH255_42a8TJ.599	5	375	599	chrX	83025968	83025598	-	0.97541
RP41_0073F18.b1.719	64	525	719	chrX	92814526	92814987	+	0.944828
RP41_0073I22.b1.719	62	484	719	chrX	105760150	**105760572**	+	0.96134
RP41_7j6TJ.825	11	455	825	chrX	105883682	**105883238**	-	0.955504
RP41_0073I22.g1.686	96	308	686	chrX	105946666	105946454	-	0.975248
CH255_26e16TV.124	4	124	124	chrX	106982796	106982916	+	1
CH255_26e16TJ.488	3	157	488	chrX	107250095	107249941	-	0.986207
CH255_139d15TJ.828	47	828	828	chrX	107385093	107384312	-	0.993017
RP41_0056P04.g1.710	490	571	710	chrX	110577932	110578013	+	1
CH255_204l8TV.610	4	195	610	chrX	111987903	**111988094**	+	0.983957
CH255_204l8TJ.765	5	765	765	chrX	112176707	**112175947**	-	0.994334
RP43_0150G15.b1.700	420	526	700	chrX	118690417	118690523	+	1
RP43_0002M11.b1.713	65	382	713	chrX	118870259	**118870576**	+	0.996815
RP43_0150G15.g1.640	1	296	640	chrX	118920615	**118920320**	-	0.989796
CH255_11m22TJ.298	2	290	298	chrX	136937005	136937293	+	0.985981
RP43_0031J22.b1.745	61	643	745	chrX	136998721	136998139	-	0.995181
CH255_208k5TJ.633	1	355	633	chrX	137331677	137331323	-	0.993127
CH255_250k3TJ.798	17	417	798	chrX	141065424	141065024	-	0.973333
CH255_202p22TJ.830	2	830	830	chrX	142404244	142403416	-	0.980583
CH255_218m23TJ.568	217	315	568	chrX_rand	499536	499438	-	1
CH255_144d20TJ.488	430	488	488	chrX_rand	3000967	3001025	+	1
RP43_119d23TJ.222	1	222	222	chrY	2473239	2473460	+	1
RP43_0164F08.b1.714	420	588	714	chrY	2500817	2500985	+	0.970588
RP43_0164F08.g1.619	7	493	619	chrY	6407537	6407051	-	0.990025
CH255_44a15TV.924	1	371	924	chrY	6521375	6521745	+	0.985423
RP41_0056J10.b1.748	54	203	748	chrY	13336250	13336399	+	0.952055
CH255_254i8TJ.363	61	125	363	chrY	13383348	13383284	-	0.982143
RP43_98i4TV.703	253	323	703	chrY	13492069	13492139	+	1
CH255_205h24TJ.681	318	419	681	chrY	13544028	**13544129**	+	0.939394
RP43_0040H16.g1.631	88	456	631	chrY	13585498	13585866	+	0.986111
RP43_98i4TJ.863	469	681	863	chrY	13625083	13624871	-	0.990291
CH255_254i8TV.626	154	248	626	chrY	13718331	**13718237**	-	0.968085
RP43_0040H16.b1.699	217	317	699	chrY	13760530	13760430	-	0.990099
RP41_0056J10.g1.721	47	380	721	chrY	13881283	13880950	-	0.964744
RP41_18b7TV.876	490	698	876	chrY	14587156	14587364	+	0.949495
RP43_0164C02.g1.617	399	511	617	chrY	15490078	15489966	-	1
RP43_124n9TJ.460	7	295	460	chrY	16409394	**16409682**	+	0.995633
RP43_0040J05.b1.705	57	301	705	chrY	16601036	**16600792**	-	0.973684
RP43_0164C02.b1.703	68	548	703	chrY	16989389	16988909	-	0.982278
CH255_217i4TJ.752	536	752	752	chrY	17329701	17329917	+	0.978261
CH255_236n10TV.564	1	151	564	chrY	17345958	**17346108**	+	0.991736
CH255_125o18TV.116	6	116	116	chrY	17427557	**17427447**	-	1
CH255_200k22TJ.589	5	137	589	chrY	17444320	17444188	-	0.972222

RP41_0155H16.b1.758	99	192	758	chrY	18528498	18528591	+	0.914894
CH255_262b2TV.689	632	689	689	chrY	18688535	18688592	+	1
RP41_0049M08.g1.728	154	246	728	chrY	18970544	18970452	-	0.903226
RP41_0155G22.b1.757	464	535	757	chrY	19045950	**19046021**	+	0.952381
RP41_0155H16.g1.760	431	628	760	chrY	19148443	**19148246**	-	0.923077
RP43_134o11TJ.865	574	865	865	chrY	19992584	**19992875**	+	0.984694
RP43_134o11TV.640	393	460	640	chrY	20154502	**20154435**	-	0.970588
RP41_0054D06.g1.762	83	218	762	chrY	20539044	20539179	+	0.942149
RP43_129j10TV.834	259	319	834	chrY	49924679	49924739	+	0.983607
CH255_204j16TJ.283	216	283	283	chrY	49924679	49924746	+	1

1467 BACs which hybridized with PTERV1 *gag* and *env* probes were end-sequenced and mapped against the human genome assembly by quality sequence alignment Insertion sites were refined based on the placement of 2 or more BACs from a species to the same location and highlighted by black border. Different species are highlighted by different colors(Red GGO,Green PTR, Light blue MMU, Blue PAN).
