# Supplementary material for: Lineage-Specific Expansions of Retroviral Insertions within the Genomes of African Great Apes but Not Humans and Orangutans
Source: PLoS Biol. 2005 Mar 1;3(4):e110. doi: 10.1371/journal.pbio.0030110 (PMC1054887; doi:10.1371/journal.pbio.0030110)
Supplement: Supplementary file 10 [file pbio.0030110.st003.pdf]

Table S3

Retroviral map intervals that potentially overlap between species*.

BACID	Chrom	begin	end		% Identity
	Human Coordinates (Build34 assembly)				
*RP41_31a20TJ.836	chr1	119530260	**119530321**	+	95.2%
*CH250_168c7TJ.825	chr1	119664263	**119664347**	+	96.5%
*RP41_31a20TV.880	chr1	119690678	**119690520**	-	98.1%
RP41_0065D07.g1.705	chr1	119703847	119703734	-	94.7%
*CH250_168c7TV.892	chr1	119808674	**119808161**	-	97.3%
*RP41_0082H03.g1.753	chr10	63369779	**63369930**	+	96.1%
*CH250_82f19TV.838	chr10	63402978	**63403544**	+	94.5%
*CH250_61n13TV.711	chr10	63514427	**63514236**	-	95.3%
*RP41_0082H03.b1.725	chr10	63523433	**63523327**	-	94.4%
*RP43_0002J22.g1.634	chr12	31775357	**31775588**	+	98.7%
*CH250_21m16TJ.537	chr12	31793783	**31793876**	+	94.7%
*RP43_0088A08.b1.682	chr12	31889282	**31889015**	-	96.6%
*CH250_92e23TV.880	chr12	31987590	**31987363**	-	94.3%
*CH250_172c10TJ.846	chr13	95686016	**95686249**	+	97.0%
*RP41_0050D04.g1.751	chr13	95732167	95732440	+	94.5%
*RP41_0068D04.b1.774	chr13	95754130	**95754255**	+	93.7%
*CH250_23a7TJ.466	chr13	95821229	**95821075**	-	96.1%
CH250_172c10TV.888	chr13	95843921	95843847	-	97.3%
*RP41_0050D04.b1.776	chr13	95888828	**95888775**	-	94.4%
*RP43_0161K06.g1.620	chr14	18946695	**18947124**	+	97.0%
*RP41_10g5TJ.750	chr14	19098959	**19099313**	+	94.1%
*RP41_0072B21.g1.699	chr14	19110956	**19110864**	-	94.6%
*RP43_0161K06.b1.717	chr14	19116461	**19115950**	-	98.4%
*CH255_12i11TV.399	chr18	49704814	**49705208**	+	97.7%
*RP41_0061O18.b1.788	chr18	49789665	**49789840**	+	96.0%
*CH255_12i11TJ.302	chr18	49884393	**49884123**	-	98.2%
*RP41_18k21TJ.852	chr18	49965079	**49965026**	-	96.3%

Summary of potential orthologous PTERV1 insertion sites by species

	Chimp	Gorilla	Baboon	Macaque
Chimp	X	X	X	X
Gorilla	1	X	X	X
Baboon	1	2	X	X
Macaque	3	0	5	X

*CH250_148j3TV.691	chr2	4209530	**4209765**	+	91.9%
*RP41_0161O24.g1.688	chr2	4247027	**4247105**	+	94.9%
*CH250_148j3TJ.728	chr2	4367524	**4367292**	-	94.4%
*RP41_0053H13.g1.693	chr2	4378320	**4377828**	-	94.3%
*CH250_12d8TJ.810	chr2	142195969	**142196106**	+	97.8%
*RP43_0151J13.b1.721	chr2	142199448	**142199733**	+	97.9%
*CH250_86e17TJ.850	chr2	142337324	**142337220**	-	100.0%
CH250_63j16TJ.736	chr2	142348857	142348765	-	96.8%
CH250_9j16TV.534	chr2	142348857	142348765	-	96.8%
*RP43_0070B15.g1.633	chr2	142355976	**142355468**	-	97.6%
*CH250_92j13TJ.838	chr2	143662193	**143662258**	+	98.5%
*RP43_114h16TJ.777	chr2	143684971	**143685693**	+	99.3%
*CH250_172e2TV.874	chr2	143762549	**143762472**	-	100.0%
CH250_92j13TV.809	chr2	143811041	143810959	-	95.2%
*RP43_114h16TV.760	chr2	143880427	**143880367**	-	96.7%
*CH255_202k13TV.579	chr7	39874303	**39874716**	+	100.0%
*RP41_0088E12.g1.723	chr7	39945430	**39945544**	+	91.3%
*CH255_202k13TJ.566	chr7	40092429	**40092285**	-	99.3%
*RP41_0088E12.b1.746	chr7	40105977	**40105865**	-	98.2%
*RP41_18d7TJ.837	chr9	88209117	**88209380**	+	94.3%
*CH250_150f13TJ.843	chr9	88246399	**88246482**	+	96.4%
*RP41_18d7TV.767	chr9	88419834	**88419654**	-	95.6%
*CH250_170p12TJ.842	chr9	88525961	**88525860**	-	98.0%
*CH255_205h24TJ.681	chrY	13544028	**13544129**	+	94.1%
*RP43_0040H16.g1.631	chrY	13585498	**13585866**	+	98.6%
*RP43_98i4TJ.863	chrY	13625083	**13624871**	-	99.1%
*CH255_254i8TV.626	chrY	13718331	**13718237**	-	96.8%

*Each BAC was T7 and SP6 end-sequenced. Sequences were mapped to the human genome assembly (orientation is indicated +/-)
