# Supplementary material for: Lineage-Specific Expansions of Retroviral Insertions within the Genomes of African Great Apes but Not Humans and Orangutans
Source: PLoS Biol. 2005 Mar 1;3(4):e110. doi: 10.1371/journal.pbio.0030110 (PMC1054887; doi:10.1371/journal.pbio.0030110)
Supplement: Supplementary file 11 [file pbio.0030110.st004.pdf]

Table S4 A. Genetic distance (Kimura) for *gag -en* v portions				
	PTR	GGO	MMU	PAN
PTR	0.028 +/- 0.003			
GGO	0.038 +/- 0.003	0.041+/- 0.003		
MMU	0.092 +/- 0.006	0.096+/- 0.006	0.103 +/- 0.007	
PAN	0.097+/-0.007	0.101+/- 0.007	0.106+/- 0.007	0.090+/- 0.006

*Portions of the gag and env genes (X=823 bp) were resequenced for n PTERV1 elements from common chimpanzee (PTR), gorilla (GGO), rhesus macaque (MMU) and the olive baboon (PAN). The number of substitutions between and within groups is compared (Kimura 2-parameter model).

**Overall substitution among all elements was 0.073 +/-0.004

Table S4 B. Genetic distance (Kimura) for LTR portions.				
	PTR	GGO	MMU	PAN
PTR	0.031 +/- 0.006			
GGO	0.039 +/- 0.006	0.037+/- 0.006		
MMU	0.140 +/- 0.015	0.143 +/- 0.015	0.142+/- 0.016	
PAN	0.250 +/- 0.023	0.245 +/- 0.023	0.181+/- 0.018	0.242 +/- 0.020

*The number of substitutions (K2M) for LTR (295 bp) sequence was compared between and within species.

**Overall substitution among all elements was 0.157+/-0.013
