# Supplementary material for: Lineage-Specific Expansions of Retroviral Insertions within the Genomes of African Great Apes but Not Humans and Orangutans
Source: PLoS Biol. 2005 Mar 1;3(4):e110. doi: 10.1371/journal.pbio.0030110 (PMC1054887; doi:10.1371/journal.pbio.0030110)
Supplement: Supplementary file 12 [file pbio.0030110.st005.pdf]

Table S5 : Distribution of LTR mismatches for each species (observed) compared with a Poisson distribution (expected).

	Chimp					Gorilla					Baboon					Macaque				
	mismatch	P(X)	observed	expected	X^2	mismatch	P(X)	observed	expected	X^2	mismatch	P(X)	observed	expected	X^2	mismatch	P(X)	observed	expected	X^2
	0	0.033	3	0.894	4.957	0	0.055	4	1.651	3.344	0	0.208	6	4.363	0.614	0	0.209	6	4.808	0.296
	1	0.113	3	3.048	0.001	1	0.160	6	4.787	0.307	1	0.326	5	6.856	0.502	1	0.327	7	7.526	0.037
	2	0.192	3	5.192	0.926	2	0.231	5	6.941	0.543	2	0.257	5	5.387	0.028	2	0.256	5	5.890	0.134
	3	0.218	6	5.897	0.002	3	0.224	5	6.710	0.436	>=3	0.204	5	4.278	0.122	>=3	0.190	5	4.373	0.090
	4	0.186	3	5.024	0.815	4	0.162	4	4.865	0.154										
	5	0.127	5	3.424	0.726	>=5	0.165	6	4.955	0.220										
	>=6	0.122	4	3.294	0.151															
Mean	3.41					2.90					1.57					1.57				
SD	2.21				df=5	2.31				df=4	1.43				df=2	1.50				df=2
Sum			27	27	7.577			30	30	5			21	21	1			23	23	0.556
P(CHITEST)					>0.1					>0.2					>0.9					>0.7

A Poisson distribution was computed based on the mean number of mismatch for each sepecies. A X^2 test was performed between observed and epected value.
