# Supplementary material for: Lineage-Specific Expansions of Retroviral Insertions within the Genomes of African Great Apes but Not Humans and Orangutans
Source: PLoS Biol. 2005 Mar 1;3(4):e110. doi: 10.1371/journal.pbio.0030110 (PMC1054887; doi:10.1371/journal.pbio.0030110)
Supplement: Supplementary file 13 [file pbio.0030110.st006.pdf]

Table S6 Mapping of solo LTR and full length PTERV1 elements in human genome (Build34)

Chromosor	Begin	End	Map Interval	Class
chr1	54731794	54732703	909	Solo LTR
chr1	111119344	111121314	1970	Solo LTR
chr1	147822764	147824602	1838	Solo LTR
chr1	235744256	235746249	1993	Solo LTR
chr2	79983496	79985117	1621	Solo LTR
chr2	142276933	142278773	1840	Solo LTR
chr2	147493981	147495817	1836	Solo LTR
chr3	50530464	50532305	1841	Solo LTR
chr3	58385331	58387309	1978	Solo LTR
chr3	59005921	59007769	1848	Solo LTR
chr3	71863426	71865395	1969	Solo LTR
chr3	76001258	76003104	1846	Solo LTR
chr3	131090914	131091886	972	Solo LTR
chr3	131092402	131092875	473	Solo LTR
chr4	32142797	32144545	1748	Solo LTR
chr4	33936015	33937967	1952	Solo LTR
chr4	68950775	68951775	1000	Solo LTR
chr4	80752308	80754173	1865	Solo LTR
chr4	93012169	93014016	1847	Solo LTR
chr4	114310965	114312946	1981	Solo LTR
chr4	161088248	161089257	1009	Solo LTR
chr4	161115289	161117147	1858	Solo LTR
chr5	23280766	23281768	1002	Solo LTR
chr5	76372398	76374236	1838	Solo LTR
chr5	94941557	94942551	994	Solo LTR
chr5	108038073	108039920	1847	Solo LTR
chr6	77268274	77270259	1985	Solo LTR
chr7	22641392	22642204	812	Solo LTR
chr7	39493688	39495534	1846	Solo LTR
chr7	54299299	54338420	39121	Solo LTR
chr7	55575949	55576910	961	Solo LTR
chr7	56168597	56169228	631	Solo LTR
chr7	77818044	77819881	1837	Solo LTR
chr7	83072736	83074591	1855	Solo LTR
chr8	8083952	8084093	141	Solo LTR
chr8	8084339	8085913	1574	Solo LTR
chr8	11922537	11924372	1835	Solo LTR
chr8	33536566	33538419	1853	Solo LTR
chr8	103140826	103142668	1842	Solo LTR
chr8	140003428	140005287	1859	Solo LTR
chr9	2755619	2757582	1963	Solo LTR
chr10	34241444	34243276	1832	Solo LTR
chr10	65261618	65263587	1969	Solo LTR
chr10	82124726	82126493	1767	Solo LTR
chr10	131895667	131896517	850	Solo LTR
chr11	5605466	5607326	1860	Solo LTR
chr11	39982191	39983029	838	Solo LTR
chr11	40015647	40016641	994	Solo LTR
chr11	60034604	60036400	1796	Solo LTR

chr11	73069490	73070427	937	Solo LTR
chr11	88982422	88983276	854	Solo LTR
chr12	4697481	4699442	1961	Solo LTR
chr12	14302828	14304794	1966	Solo LTR
chr12	24260383	24261357	974	Solo LTR
chr12	37773613	37775464	1851	Solo LTR
chr12	58001769	58002753	984	Solo LTR
chr12	84423969	84424816	847	Solo LTR
chr12	99964540	99966373	1833	Solo LTR
chr12	126858199	126860056	1857	Solo LTR
chr13	44049710	44051713	2003	Solo LTR
chr14	26303548	26304397	849	Solo LTR
chr14	42255367	42257213	1846	Solo LTR
chr14	43826174	43828022	1848	Solo LTR
chr14	85474775	85476653	1878	Solo LTR
chr15	51831796	51833760	1964	Solo LTR
chr15	59815366	59817306	1940	Solo LTR
chr16	8194026	8195871	1845	Solo LTR
chr17	19525105	19526807	1702	Solo LTR
chr17	27118580	27120431	1851	Solo LTR
chr17	35844360	35846211	1851	Solo LTR
chr18	12486411	12487289	878	Solo LTR
chr18	27641857	27642789	932	Solo LTR
chr18	47143822	47145666	1844	Solo LTR
chr18	66269942	66271910	1968	Solo LTR
chr19	23698157	23699995	1838	Solo LTR
chr19	58130470	58132305	1835	Solo LTR
chr20	46690227	46692019	1792	Solo LTR
chr20	58818500	58820345	1845	Solo LTR
chr22	47660230	47662073	1843	Solo LTR
chrX	6876493	6878342	1849	Solo LTR
chrX	39894638	39895717	1079	Solo LTR
chrX	80366936	80367931	995	Solo LTR
chrX	88211317	88213089	1772	Solo LTR
chrX	92842195	92844158	1963	Solo LTR
chrX	136109951	136110961	1010	Solo LTR
chrY	2349016	2349851	835	Solo LTR
chrY	14203508	14205409	1901	Solo LTR
chrY	16554654	16554754	100	Solo LTR
chrY	17907216	17909046	1830	Solo LTR
chrY	20120263	20121104	841	Solo LTR
chr1	54730942	54731794	852	Full length PtERV1
chr1	66309175	66310827	1652	Full length PtERV1
chr1	111203341	111205052	1711	Full length PtERV1
chr1	113881516	113882476	960	Full length PtERV1
chr1	180520473	180522296	1823	Full length PtERV1
chr2	31489958	31491925	1967	Full length PtERV1
chr2	33873483	33875289	1806	Full length PtERV1
chr2	65482582	65483578	996	Full length PtERV1
chr2	70623549	70625353	1804	Full length PtERV1
chr2	83478811	83480794	1983	Full length PtERV1
chr2	84173674	84175510	1836	Full length PtERV1

chr2	143808621	143810603	1982	Full length PtERV1
chr2	155924592	155925566	974	Full length PtERV1
chr2	209266156	209267519	1363	Full length PtERV1
chr2	211432626	211433614	988	Full length PtERV1
chr2	215899808	215901433	1625	Full length PtERV1
chr3	39323801	39325663	1862	Full length PtERV1
chr3	44767517	44768526	1009	Full length PtERV1
chr3	78341533	78343503	1970	Full length PtERV1
chr3	87697533	87699378	1845	Full length PtERV1
chr3	88169218	88170218	1000	Full length PtERV1
chr3	95502275	95504169	1894	Full length PtERV1
chr3	131091886	131092402	516	Full length PtERV1
chr3	163792615	163794459	1844	Full length PtERV1
chr4	47267059	47269060	2001	Full length PtERV1
chr4	47677557	47679397	1840	Full length PtERV1
chr4	72970115	72971117	1002	Full length PtERV1
chr4	74268840	74270854	2014	Full length PtERV1
chr4	108796279	108797266	987	Full length PtERV1
chr4	117350389	117353847	3458	Full length PtERV1
chr4	161093604	161095602	1998	Full length PtERV1
chr5	18846348	18848229	1881	Full length PtERV1
chr5	42950470	42952440	1970	Full length PtERV1
chr5	57143465	57145445	1980	Full length PtERV1
chr5	59024597	59026441	1844	Full length PtERV1
chr5	74663683	74665646	1963	Full length PtERV1
chr5	76307225	76308210	985	Full length PtERV1
chr5	94940710	94941557	847	Full length PtERV1
chr5	156207051	156208072	1021	Full length PtERV1
chr6	38443224	38444105	881	Full length PtERV1
chr6	147748534	147750506	1972	Full length PtERV1
chr6	154182232	154187051	4819	Full length PtERV1
chr6	167556086	167557067	981	Full length PtERV1
chr6	167567228	167568075	847	Full length PtERV1
chr7	10980236	10982204	1968	Full length PtERV1
chr7	36413748	36415591	1843	Full length PtERV1
chr7	39586669	39588495	1826	Full length PtERV1
chr7	48878172	48880142	1970	Full length PtERV1
chr7	55576910	55577841	931	Full length PtERV1
chr7	55717796	55719670	1874	Full length PtERV1
chr7	61072410	61073374	964	Full length PtERV1
chr7	137131794	137132793	999	Full length PtERV1
chr8	54656576	54657528	952	Full length PtERV1
chr8	98043553	98044393	840	Full length PtERV1
chr10	43132513	43134470	1957	Full length PtERV1
chr10	61323212	61325181	1969	Full length PtERV1
chr10	85544318	85545325	1007	Full length PtERV1
chr10	104627660	104628659	999	Full length PtERV1
chr10	104629183	104629470	287	Full length PtERV1
chr10	131909535	131910378	843	Full length PtERV1
chr11	40046726	40047729	1003	Full length PtERV1
chr11	71079518	71080523	1005	Full length PtERV1
chr11	77004432	77005278	846	Full length PtERV1

chr11	94074278	94076242	1964	Full length PtERV1
chr12	5517662	5519505	1843	Full length PtERV1
chr12	8541730	8543699	1969	Full length PtERV1
chr12	14760304	14761275	971	Full length PtERV1
chr12	31852232	31854191	1959	Full length PtERV1
chr12	32069663	32071500	1837	Full length PtERV1
chr12	36736203	36738157	1954	Full length PtERV1
chr12	37316703	37318684	1981	Full length PtERV1
chr12	40434608	40435608	1000	Full length PtERV1
chr12	58000796	58001769	973	Full length PtERV1
chr12	70892142	70900305	8163	Full length PtERV1
chr12	72504616	72505535	919	Full length PtERV1
chr12	75171893	75172866	973	Full length PtERV1
chr12	84422975	84423969	994	Full length PtERV1
chr13	22203180	22205145	1965	Full length PtERV1
chr14	19093151	19094110	959	Full length PtERV1
chr14	57058514	57060450	1936	Full length PtERV1
chr16	10391638	10393602	1964	Full length PtERV1
chr16	10566579	10567579	1000	Full length PtERV1
chr16	31596805	31597779	974	Full length PtERV1
chr16	60004977	60005950	973	Full length PtERV1
chr18	30487910	30488905	995	Full length PtERV1
chr18	31683744	31685710	1966	Full length PtERV1
chr19	32975282	32977211	1929	Full length PtERV1
chr19	42709688	42711515	1827	Full length PtERV1
chr19	48358042	48359033	991	Full length PtERV1
chr20	20200851	20202695	1844	Full length PtERV1
chr20	33438422	33438945	523	Full length PtERV1
chr21	14172059	14172381	322	Full length PtERV1
chr22	15459767	15459870	103	Full length PtERV1
chr22	15470551	15471526	975	Full length PtERV1
chrX	41695832	41696588	756	Full length PtERV1
chrX	64432874	64433846	972	Full length PtERV1
chrX	136956475	136957449	974	Full length PtERV1
chrY	6357189	6357273	84	Full length PtERV1
chrY	6628195	6630071	1876	Full length PtERV1
chrY	13584728	13585720	992	Full length PtERV1
chrY	13656784	13657782	998	Full length PtERV1
chrY	14169506	14170285	779	Full length PtERV1
chrY	16502113	16504048	1935	Full length PtERV1
chrY	16553656	16554654	998	Full length PtERV1
chrY	16830021	16830881	860	Full length PtERV1
chrY	20121104	20122098	994	Full length PtERV1
chrY	20361982	20362957	975	Full length PtERV1

Using LTR and PtERV(LTR sequence exclued) Megablast against Chimpanzee assembley (2003). Any loci hited by LTR and PtERV (both of them are within 10Kb to each other) are considered as Full-length, alternativley only LTR hit loci are defined as Solo LTR.The the chimpanzee coordinate were transfered to corespondent human assembly (build34) coordinates by the program liftover (see the Methods for details).
