# Supplementary material for: Lineage-Specific Expansions of Retroviral Insertions within the Genomes of African Great Apes but Not Humans and Orangutans
Source: PLoS Biol. 2005 Mar 1;3(4):e110. doi: 10.1371/journal.pbio.0030110 (PMC1054887; doi:10.1371/journal.pbio.0030110)
Supplement: Supplementary file 14 [file pbio.0030110.st007.pdf]

Table S7 PTERV1 distribution in chimpanzee genome

	Full-Length*	Solo LTR**
Genic	14	8
Non-genic	93	82
Total	107	90

*Full-length elements are those which extend beyond the LTR into gag and/or env portion. Because of the repetitive nature of these elements, Arachne failed to provide continuous sequence these elements.

**Solo LTR are those which consist solely of LTR sequence.
