# Supplementary material for: Lineage-Specific Expansions of Retroviral Insertions within the Genomes of African Great Apes but Not Humans and Orangutans
Source: PLoS Biol. 2005 Mar 1;3(4):e110. doi: 10.1371/journal.pbio.0030110 (PMC1054887; doi:10.1371/journal.pbio.0030110)
Supplement: Supplementary file 15 [file pbio.0030110.st008.pdf]

Table S8 The keys (numbers) and their corresponding clones for Figure 4.

Key	Clone
1	Gorilla 10g22
2	Gorilla 115h12
3	Gorilla 126l9
4	Gorilla 138m5
5	Gorilla 24l1
6	Gorilla 19p24
7	Gorilla 144o12
8	Gorilla 214f22
9	Gorilla 211n3
10	Gorilla 44a12
11	Gorilla 254i8
12	Gorilla 138p14
13	Gorilla 138p14b
14	Gorilla 204c16
15	Gorilla 204c16
16	Gorilla 218g10
17	Gorilla 130k3
18	Gorilla 199e9
19	Gorilla 230l15
20	Gorilla 266e22
21	Gorilla 12k13
22	Chimpanzee 16i6
23	Gorilla 270g5
24	Gorilla 254p6
25	Gorilla 30a20
26	Chimpanzee 94i7
27	Chimpanzee 14e15
28	Chimpanzee 86j13
29	Chimpanzee 41h9
30	Chimpanzee 40h16
31	Chimpanzee 25e22
32	no sample
33	Chimpanzee 17o20
34	Chimpanzee 70g8
35	Chimpanzee 68b15
36	Chimpanzee 90k24
37	Chimpanzee 31j22
38	Chimpanzee 16o7
39	Chimpanzee 13o3
40	Chimpanzee 13o6
41	Chimpanzee 15b12
42	Chimpanzee 62i9,33p19
43	Chimpanzee 16o8
44	Chimpanzee 34j17, 28b10
45	Chimpanzee 68m12, 88a8
46	Chimpanzee 94c19
47	Chimpanzee 1d2
48	Chimpanzee 76g32, 1m15
49	Chimpanzee 92j15

50	Chimpanzee 31p4
51	Chimpanzee 76l3
52	Chimpanzee 94a1
53	Chimpanzee 34k3
54	Chimpanzee 27n19
55	Chimpanzee 20h17
56	Chimpanzee 68f21
57	Gorilla 42d19
58	Chimpanzee 44a24
59	Gorilla 28o3
60	Chimpanzee 125n22
61	Chimpanzee 92g8
62	Chimpanzee 126n22
63	Chimpanzee 70b15
64	Macaque 30k8
65	Macaque 45a22
66	Baboon 54d6
67	Baboon 56k19
68	Macaque 5a4
69	Macaque 47d15
70	Macaque 35j16
71	Macaque 61l7
72	Macaque 41a24
73	Baboon 51o18
74	Baboon 61h16
75	Baboon 58l4
76	Baboon 63o23
77	Baboon 67p7
78	Macaque 175n18
79	Macaque 165n13
80	Baboon 65k2
81	Macaque 80c22
82	Baboon 50d4
83	Baboon 61o18
84	Baboon 60b6
85	Baboon 55d7
86	Baboon 63l15
87	Baboon 65k8
88	Baboon 51m3
89	Baboon 59h10
90	Baboon 52j10
91	Baboon 65d7
92	Baboon 70n15
93	Baboon 207
94	Macaque 18l14
95	Macaque 147l22
96	Macaque 82l23
97	Macaque 82l23
98	Macaque 83m4
99	Macaque 93j17
